# Wavy Graphene Nanoribbons Containing Periodic Eight‐Membered Rings for Light‐Emitting Electrochemical Cells

**DOI:** 10.1002/anie.202415670

**Published:** 2024-10-31

**Authors:** Sebastian Obermann, Xin Zhou, L. Andrés Guerrero‐León, Gianluca Serra, Steffen Böckmann, Yubin Fu, Evgenia Dmitrieva, Jin‐Jiang Zhang, Fupin Liu, Alexey A. Popov, Andrea Lucotti, Michael Ryan Hansen, Matteo Tommasini, Yungui Li, Paul W. M. Blom, Ji Ma, Xinliang Feng

**Affiliations:** ^1^ Center for Advancing Electronics Dresden (cfaed) & Faculty of Chemistry and Food Chemistry Technische Universität Dresden Mommsenstrasse 4 01069 Dresden Germany; ^2^ Max-Planck-Institute for Polymer Research Ackermannweg 10 55128 Mainz Germany; ^3^ Department of Chemistry Materials Chemical Engineering Politecnico di Milano Piazza Leonardo da Vinci 32 20133 Milano Italy; ^4^ Institute of Physical Chemistry University of Münster 48149 Münster Germany; ^5^ Leibniz Institute for Solid State and Materials Research Helmholtzstr. 20 01069 Dresden Germany; ^6^ College of Materials Science and Opto-Electronic Technology & Center of Materials Science and Optoelectronics Engineering University of Chinese Academy of Science 100049 Beijing P. R. China; ^7^ Max-Planck-Institute of Microstructure Physics Weinberg 2 06120 Halle Germany

**Keywords:** precision synthesis, graphene nanoribbon, wavy geometry, non-hexagonal rings, organic light emitting cells

## Abstract

Precision graphene nanoribbons (GNRs) offer distinctive physicochemical properties that are highly dependent on their geometric topologies, thereby holding great potential for applications in carbon‐based optoelectronics and spintronics. While the edge structure and width control has been a popular strategy for engineering the optoelectronic properties of GNRs, non‐hexagonal‐ring‐containing GNRs remain underexplored due to synthetic challenges, despite offering an equally high potential for tailored properties. Herein, we report the synthesis of a wavy GNR (**wGNR**) by embedding periodic eight‐membered rings into its carbon skeleton, which is achieved by the A_2_B_2_‐type Diels–Alder polymerization between dibenzocyclooctadiyne (**6**) and dicyclopenta[*e*,*l*]pyrene‐5,11‐dione derivative (**8**), followed by a selective Scholl reaction of the obtained ladder‐type polymer (**LTP**) precursor. The obtained **wGNR**, with a length of up to 30 nm, has been thoroughly characterized by solid‐state NMR, FT‐IR, Raman, and UV/Vis spectroscopy with the support of DFT calculations. The non‐planar geometry of **wGNR** efficiently prevents the inter‐ribbon π–π aggregation, leading to photoluminescence in solution. Consequently, the **wGNR** can function as an emissive layer for organic light‐emitting electrochemical cells (OLECs), offering a proof‐of‐concept exploration in implementing luminescent GNRs into optoelectronic devices. The fast‐responding OLECs employing **wGNR** will pave the way for advancements in OLEC technology and other optoelectronic devices.

## Graphical abstract

A wavy graphene nanoribbon (**wGNR**) that incorporates periodic eight‐membered rings into its backbone has been synthesized through bottom‐up solution synthesis. This distinctive non‐planar geometry gives **wGNR** excellent dispersibility in solution and unlocks its emissive features, making it an emissive layer for organic light‐emitting electrochemical cells.

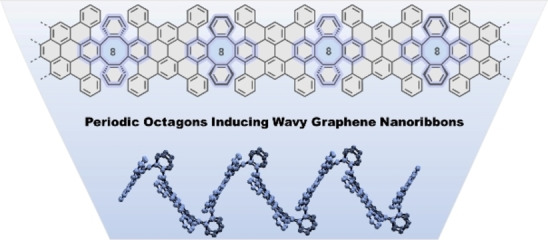

## Introduction

Structurally well‐defined graphene nanoribbons (GNRs) have been the center of substantial research efforts for more than a decade,[^1^, ^2^, ^3^, ^4^, ^5^] and their unique opto‐electronic properties foreshadow appealing applications in nanoelectronics and spintronics.[^6^, ^7^, ^8^, ^9^, ^10^, ^11^] It is well known that the physicochemical properties of GNRs are highly dictated by their edge structure and width.[^12^, ^13^, ^14^, ^15^] Bottom‐up synthetic strategies provide a powerful method for precise control over their edge topology, resulting in tunable band gaps and transport properties as well as improved liquid phase processability, demonstrated by numerous works over the past decade.[^16^, ^17^, ^18^, ^19^] Aside from altering the size and edge topology in solely hexagonal carbon lattices, the introduction of non‐hexagonal rings in graphene nanostructures offers an additional avenue to modify their topologies and physicochemical properties.[^20^, ^21^, ^22^] For instance, the incorporation of pentagons results in a positive Gaussian curvature, whereas the implementation of heptagons and octagons gives a negative Gaussian curvature, which has been well demonstrated in nanographene chemistry.[^23^, ^24^, ^25^] Recently, various non‐benzenoid nanographenes containing non‐hexagonal rings have been synthesized in solution, exhibiting diverse redox chemistry, enhanced solubility, tunable optical properties, and energy gap modulation.[^26^, ^27^, ^28^, ^29^, ^30^, ^31^] However, the extension to non‐benzenoid GNRs is currently limited to on‐surface synthesis, achieving ring sizes from pentagons to octagons.[^32^, ^33^, ^34^, ^35^]

Despite the preliminary success of on‐surface synthesized GNRs with non‐hexagonal rings, this approach is plagued by side reactions such as isomerization, skeletal rearrangement, and low efficiency.[^36^, ^37^] For example, a pentagon‐embedded GNR was synthesized recently,^[33]^ relying on the methyl group fusion to form the five‐membered rings, but these methyl groups were often cleaved off or migrated to other positions, resulting in hexagon formation and ribbon backbone rearrangements. Additionally, attempts to obtain purely non‐hexagonal‐ring‐based GNRs through cyclodehydrogenation or fusion of polyazulenes yielded either oligomers or poorly defined ribbon structures after annealing.[^35^, ^37^] In addition to synthetic challenges, these graphene nanostructures tend to adopt a near‐planar geometry when adsorbed on metal surfaces, potentially masking the characteristic features of the non‐hexagonal rings. Conversely, the solution‐mediated synthesis of expanded non‐hexagonal‐ring embedded GNRs presents significant challenges, primarily due to the lack of suitable building blocks and efficient synthetic methods. To fully explore the properties and potential applications of non‐benzenoid GNRs, the development of a wet‐chemical synthetic strategy is imperative.

**Figure 1:**
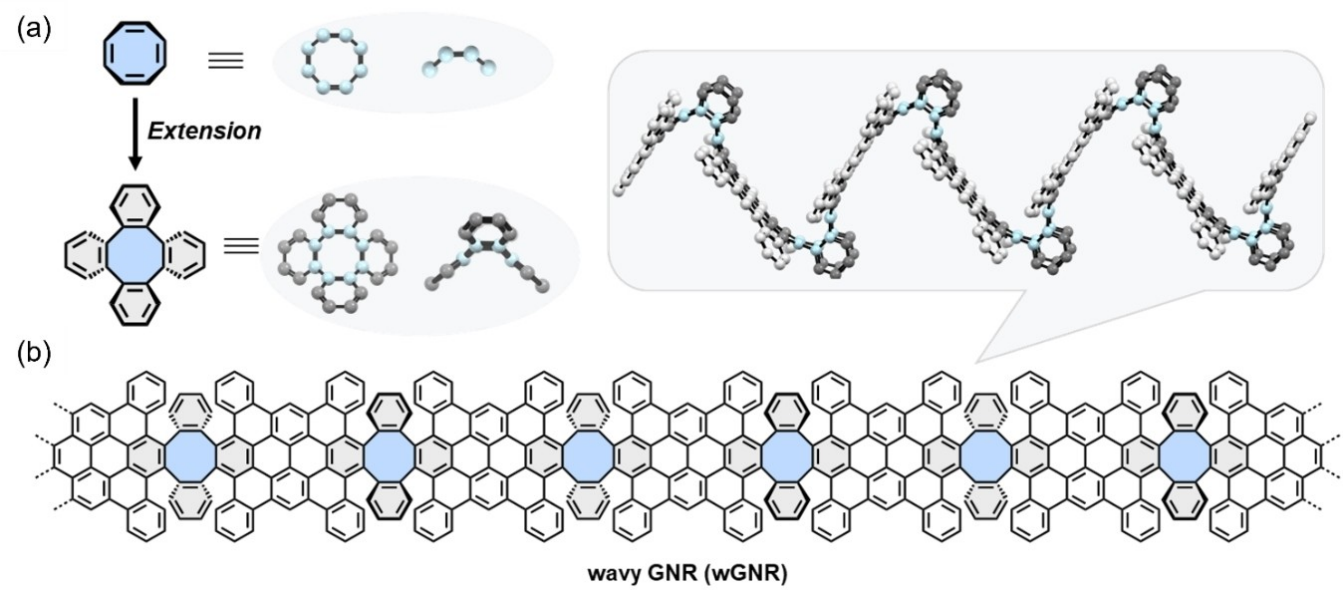
Design principle of the **wGNR** backbone. (a) Structure and geometry (top view DFT‐simulated on a HSEh1PBE/6‐31G(d) level of theory, and side view) of cyclooctatetraene and its extended structure of the tetraphenylene motif; (b) structure and presumed geometry (side view) of octagon‐embedded **wGNR** containing tetraphenylene units; the substituents are omitted for clarity.

Herein, we present the first wave‐shaped non‐benzenoid graphene nanoribbon (**wGNR**) that incorporates periodic eight‐membered rings into its backbone through bottom‐up solution‐mediated synthesis. Our design principle relies on extending the smallest sp^2^‐hybridized octagonal system, namely cyclooctatetraene (Figure 1a), by adding four phenyl rings towards a tetraphenylene motif. This extension creates a double concave configuration in the resultant π‐system.[^38^, ^39^] Due to this distinctive structural feature, the one‐dimensional extended **wGNR** based on the tetraphenylene subunits could adopt a wave‐like geometry along the ribbon backbone axis (Figure 1b). Experimentally, the **wGNR** is successfully derived from its corresponding ladder‐type polymer (**LTP**) precursor through a process involving A_2_B_2_‐Diels Alder cycloaddition polymerization of the octagon‐bearing diyne (**6**) and the dicyclopenta[*e*,*l*]pyrene‐5,11‐dione derivative (**8**) followed by a selective Scholl reaction. The obtained **wGNR** reaches a length of up to 30 nm according to gel permeation chromatography (GPC) of its corresponding **LTP** precursor. It is important to note that **wGNR** exhibits excellent dispersibility (10 mg mL^−1^ in chloroform) and emissive features (PLQY=1 %), which are not commonly observed in solution‐synthesized GNRs.[^4^, ^11^, ^40^] The chemical structure and optical properties of **wGNR** are comprehensively examined through solid‐state NMR, FT‐IR, Raman, and UV/Vis spectroscopy, complemented by DFT calculations. Furthermore, the excellent dispersibility of **wGNR** and its emissive features in the visible range enable the development of the first GNR‐based organic light‐emitting electrochemical cell, where the **wGNR** is successfully utilized as the emitter material, displaying a short turn‐on time (<10 s) and a peak luminance of 120 cd m^−2^.

## Results and Discussion

### Synthesis and Characterization of Model Compound 1

**Figure 2:**
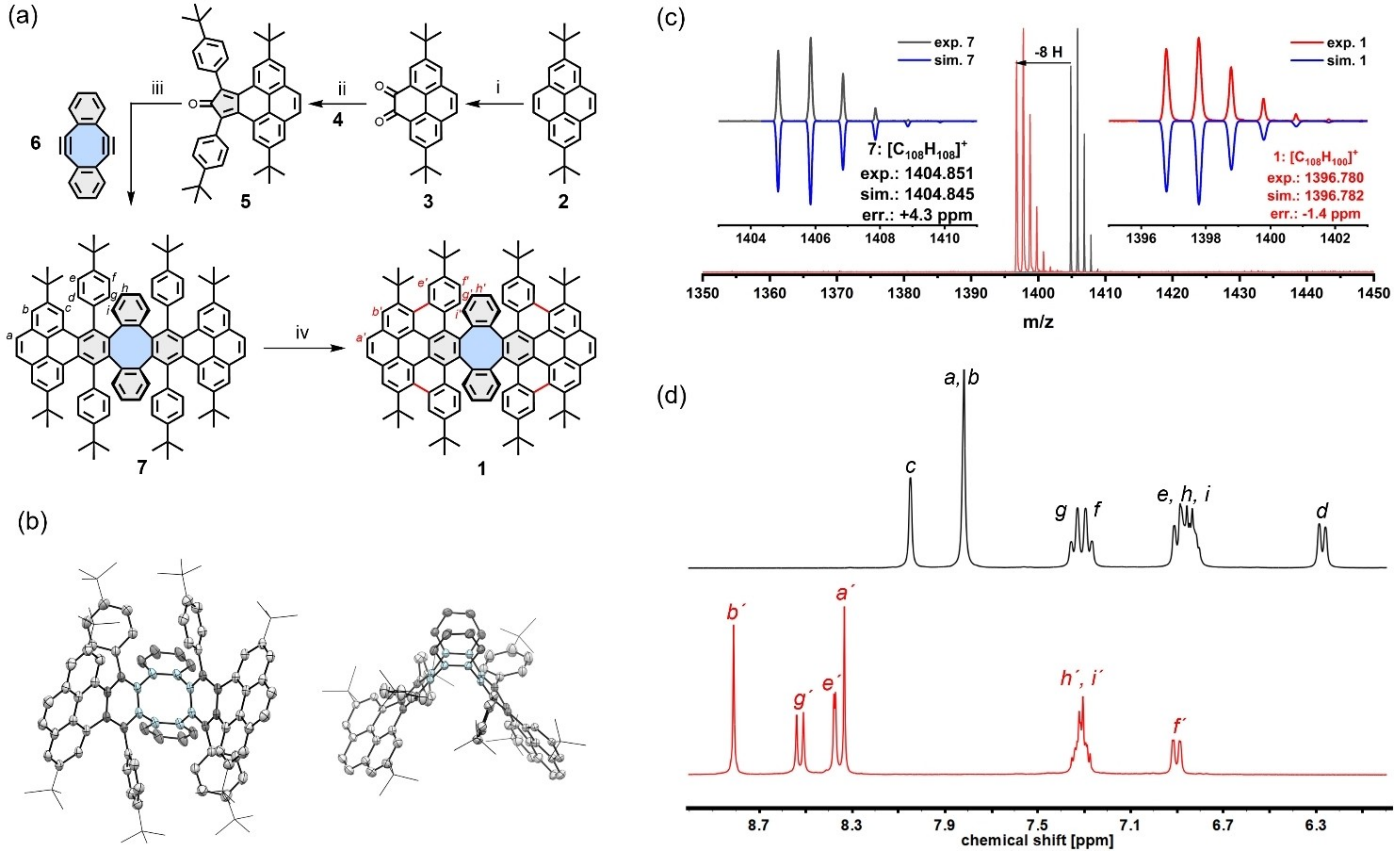
Synthesis and analysis of model compound **1**. (a) Synthesis of **1**; reagents and conditions: i) RuCl_3_, NaIO_4_, MeCN, H_2_O, CH_2_Cl_2_, rt, 90 min, 47 %; ii) KOH, 1,3‐bis(4‐(*tert‐*butyl)phenyl)propan‐2‐one (**4**), EtOH, 80 °C, 60 min, 61 %; iii) PhMe, 110 °C, overnight, 91 %; iv) DDQ (6 eq.), CH_2_Cl_2_/triflic acid (10 : 1), −40 °C, 20 min, 75 %; (b) crystal structure of compound **7** (top and side view); (c) MALDI‐TOF MS of compounds **7** and **1**; (d) aromatic region of the ^1^H NMR spectra of **7** (top) and **1** (bottom).

To examine the efficiency of the Scholl reaction on the precursor **LTP** and to unveil the non‐planar geometry of the proposed **wGNR**, model compound **1** was synthesized, as depicted in Figure 2a. The synthesis started from 2,7‐di‐*tert*‐butylpyrene, which was oxidized to the diketone by the presence of RuCl_3_ and NaIO_4_, affording compound **3** with a yield of 47 %. Subsequently, Aldol reaction with diphenylacetone **4** (see Supporting Information chapter 1.2) and KOH in EtOH gave cyclopentadienone **5** in 61 % yield. Meanwhile, the diyne building block **6** was also synthesized starting from the commercially available benzenesulfinate and 2‐bromomethylbenzonitrile in three steps (see Supporting Information chapter 1.2). Compounds **5** and **6** were then used to obtain the model compound precursor **7** in a Diels–Alder cycloaddition reaction with an excellent yield of 91 %, in which its chemical structure has been clearly revealed by single‐crystal X‐ray diffraction analysis (Figure 2b). With compound **7** in hand, we examined the selective Scholl reaction, crucial for obtaining the butterfly‐like compound **1** with intact tetraphenylene unit. As monitored by matrix‐assisted laser desorption/ionization‐time‐of‐flight (MALDI‐TOF) mass spectrometry, the reaction gave compound **1** with eight protons lost as compared to the precursor **7** (Figure 2c). The chemical structure of compound **1** was then proven by NMR spectroscopy and compared to that of **7** (Figure 2d). A full NMR investigation of **1** was possible thanks to the excellent solubility resulting from its butterfly‐like shape. The protons of compound **1** showed a low‐field shift due to the aromatization, and their total number of protons in the aromatic region was reduced from 36 to 28 signals. All protons were successfully assigned by 2D NMR experiments (see Supporting Information chapter 2). As the cyclooctatetraene unit is known for its redox chemistry,[^29^, ^30^, ^41^] cyclic voltammetry (CV) analysis coupled with spectroelectrochemical (SEC) analysis was carried out to investigate the redox properties of model compound **1** and its oxidized states (SI chapter 3). The CV exhibits two stepwise oxidations with the corresponding reversible reductions. The oxidation events appear at half‐wave potentials of 0.59 and 0.71 V *vs*. Fc/Fc^+^. The SEC data unambiguously confirms the formation of the radical cation during the first oxidation and provides its characteristic UV/Vis‐NIR and EPR features. The reduction processes occur at high negative potentials (at −2.48 and −2.54 V) indicating that compound **1** is difficult to be reduced.

### Crystallographic Analysis of Model Compound 1

**Figure 3:**
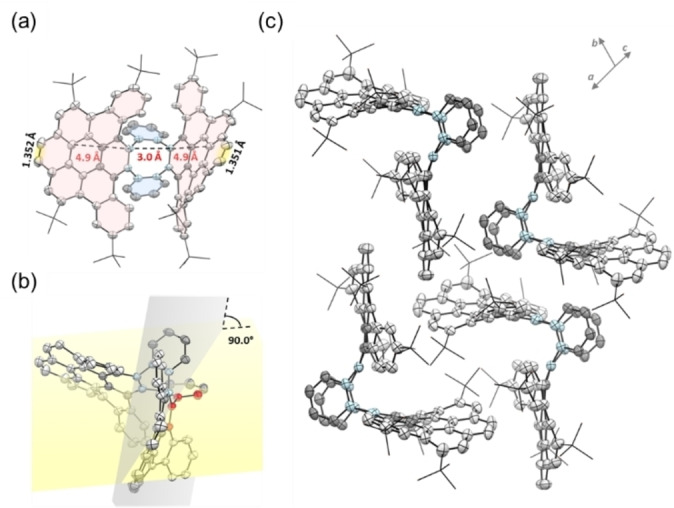
Crystal structure of model compound **1** (ORTEP drawn at the 50 % probability level). (a) Front view; (b) torsion angle along the carbon atoms marked in red; (c) crystal packing of **1**.

Suitable crystals for X‐ray crystallographic analysis were obtained for **1** (Figure 3), which was grown by slow diffusion of methanol in its CH_2_Cl_2_ solution.^[42]^ Single‐crystal X‐ray diffraction (SCRD) analysis of **1** revealed a highly distorted, butterfly‐like structure that belongs to the triclinic space group $P\bar{1}$ . As already observed by mass spectrometry and NMR spectroscopy, eight protons were removed in the cyclization of **7**, leading to the formation of two dibenzocoronene subunits (highlighted in pink in Figure 3a) in **1**. Due to the presence of the tetraphenylene motif within compound **1**, the dibenzocoronene discs face out of the observation plane, while the two benzene rings (highlighted in blue) on the eight‐membered ring face into the observation plane (Figure 3a). The carbon‐carbon bonds in the K‐regions of compound **1** exhibit distances of 1.35 Å, matching with the K‐region bond length of pyrene reported in the literature, indicating double bond character.^[42]^ The torsion angle between the dibenzocoronene moieties and the bent phenyl rings reaches up to 90° (Figure 3b), showcasing the large strain induced by the eight‐membered ring. In contrast, previously reported fully embedded eight‐membered rings do not exhibit such strong distortion and, therefore, are incapable of achieving the wavy conformation for graphene nanostructures that is possible with the presence of highly distorted tetraphenylene.[^24^, ^43^] It′s noteworthy that the considerable distance between the phenyl rings and dibenzocoronene discs in **1** poses a significant obstacle for subsequent cyclization, thus this selective Scholl reaction succeeds in leaving these positions untouched, which is essential for attaining the final butterfly‐shaped geometry of **1** while maintaining the tetraphenylene motif. In the crystal packing, the π‐surfaces of the dibenzocoronene discs are slightly slipped and face towards each other (Figure 3c), resulting in π–π‐stacks along two axes simultaneously.

### Synthesis of wGNR

**Scheme 1:**
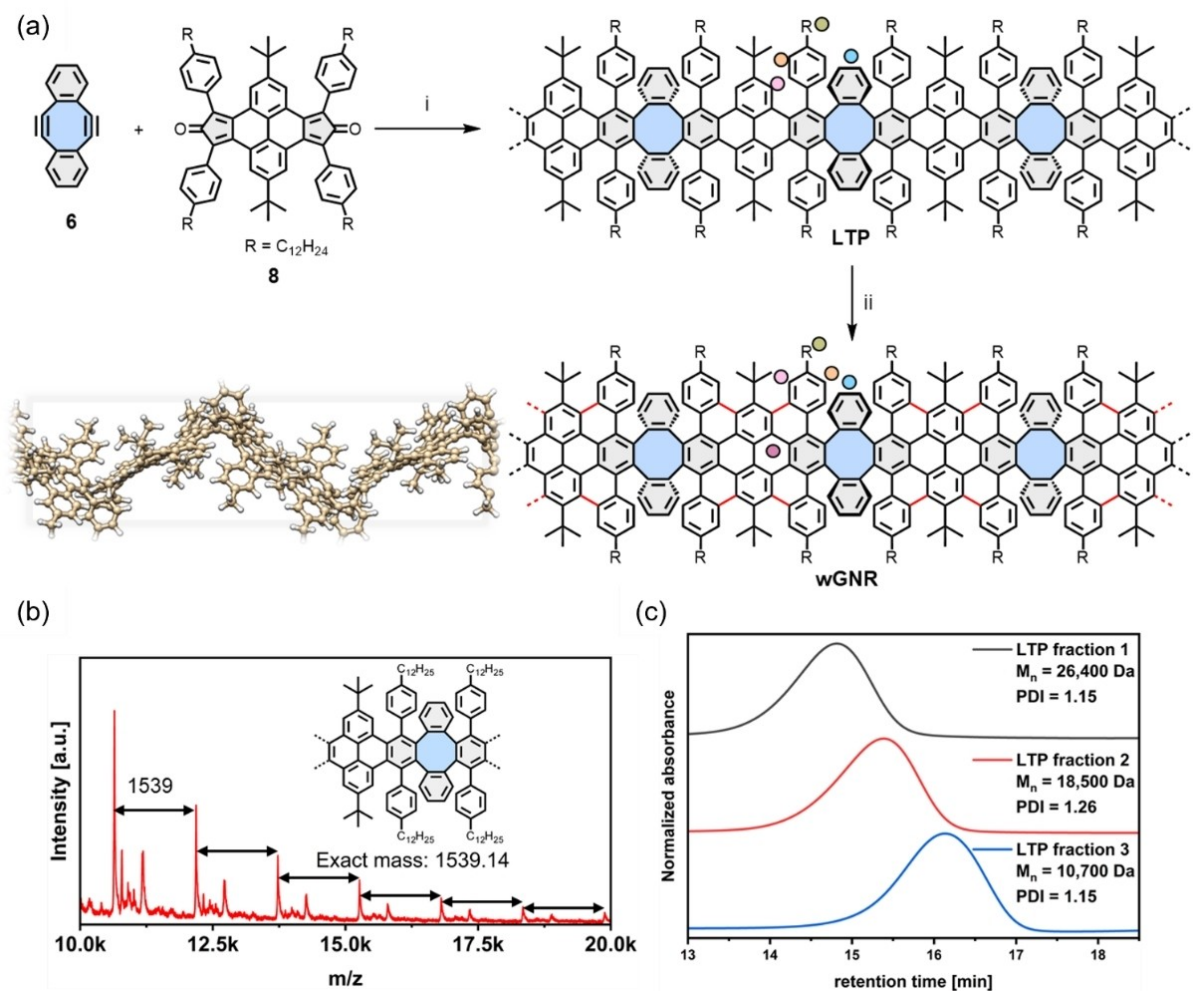
Synthesis of **wGNR** and analysis of **LTP**. (a) Synthesis of **wGNR**; reagents and conditions: (i) diphenyl ether, 265 °C, 48 h, 91 %; (ii) CH_2_Cl_2_/triflic acid (10 : 1), DDQ (1.5 eq./bond), −40 °C to rt, 3 days, 90 %; colored dots indicate vibrations marked in Figure 4a, inset is the DFT‐optimized geometry of the **wGNR** with substituents, where the dodecyl chains are replaced by methyl groups in the calculation to keep the computational burden under control; (b) linear mode MALDI‐TOF mass spectrum of **LTP**, showing periodic signals spaced by the repetition unit mass; (c) analytical GPC traces of the fractionated **LTP** (eluent: chloroform, flow rate=1.0 mL min^−1^, detection at 254 nm).

Following the confirmation of the butterfly structure **1**, the synthesis is then extended to **wGNR** by using A_2_B_2_‐type Diels–Alder polymerization of diyne **6** and cyclopentadienone **8** (for synthesis see Supporting Information chapter 1.2), where the long dodecyl chains are equipped on the ribbon periphery to assist its solubility (Scheme 1a). After heating **6** and **8** at 265 °C in diphenyl ether for two days, linear mode MALDI‐TOF‐MS of the crude **LTP** material revealed periodic signals up to *m*/*z* ca. 20 000, separated by the mass of the repetition unit of 1539 g mol^−1^ (Scheme 1b). When increasing the laser power, signals reaching up to *m*/*z* ca. 40 000 can be observed, at the cost of mass resolution (SI Figure S1). The crude **LTP** was then diluted with THF, filtrated over silica, and precipitated by the addition of methanol. After that, **LTP** was subsequently divided into several fractions by recycling GPC, which were further analyzed by analytical GPC against polystyrene standards (Scheme 1c). For the heaviest fraction (ca. 10 wt %), our analysis reveals an averaged molar mass of *M*_n_ ca. 26 400 Da and a narrow polydispersity index of 1.15. According to the size measurement from the single crystal of **7**, the longest chains of **LTP** can be estimated as 30 nm in length (*M*_z_ ca. 36 700 Da).

After obtaining and confirming the structure of **LTP**, the synthesis of **wGNR** was performed under similar conditions as for model compound **1**, using triflic acid/DDQ at temperatures ranging from −40 °C to room temperature over three days. The resulting **wGNR** possesses a wavy structure characterized by periodic octagons in its backbone, as demonstrated by the butterfly‐shaped model compound **1** and DFT calculations of **wGNR** under periodic boundary conditions (Inset in Scheme 1a, Supporting Information Chapter 5). The unique configuration of **wGNR** imparts a dispersibility of ca. 10 mg mL^−1^ in chloroform, surpassing the current state‐of‐the‐art liquid phase dispersibility achieved by other reported GNRs, which typically achieve 1–5 mg mL^−1^.[^6^, ^12^, ^44^, ^45^]

### Structural Characterizations of wGNR

The **LTP** precursor and **wGNR** were then characterized by solid‐state ^1^H and ^13^C{^1^H} MAS NMR (^1^H decoupled ^13^C magic angle spinning NMR) at a high spinning speed of 62.5 kHz (SI Chapter 11), Fourier‐transform infrared spectra (FT‐IR) spectroscopy, Raman, as well as UV/Vis spectroscopy.

**Figure 4:**
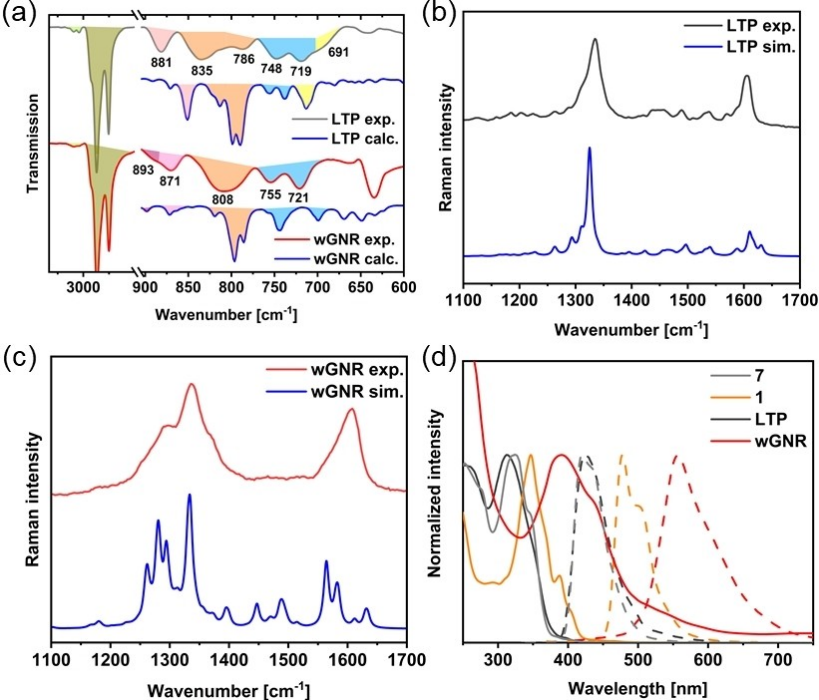
Spectroscopic data for **LTP** and **wGNR**. (a) Experimental and simulated (HSEh1PBE/6‐31G(d)) FT‐IR spectra of **LTP** and **wGNR**; (b) experimental and simulated Raman spectra of **LTP**; (c) experimental and simulated Raman spectra of **wGNR**; (d) UV/Vis (solid) and PL (dashed) of compounds **7**, **1**, **LTP** and **wGNR** in chloroform.

FT‐IR spectra were collected for compounds **7**, **1**, **LTP** and **wGNR** (Figure 4a). To assist with the assignment of the vibrational modes, DFT calculations on an HSEh1PBE/6‐31G(d) level of theory were carried out and used for comparison. Assignment tables for **7**, **1**, **LTP**, and **wGNR** can be found in the Supporting Information Chapter 6. For both **LTP** and **wGNR**, the DFT simulations closely resemble the obtained spectra. In the case of **LTP**, the SOLO mode of the pyrene moieties (CH‐wagging of an aromatic proton without adjacent protons, pink)^[46]^ can be found at 881 cm^−1^, the DUO modes (orange) at 835 cm^−1^. **wGNR** also closely resembles its simulation, showing changes in the vibrational spectrum when compared to its polymer precursor. First, the CH‐stretching modes around 3000–3100 cm^−1^ (light green) were diminished due to cyclodehydrogenation, showing only small signals in the case of **wGNR**. As proof of the graphitization process from **LTP**, a scaffold vibration of the graphitic part of **wGNR** can be assigned to the peak at 893 cm^−1^ (dark pink). As H^c^ and H^d^ (see Figure 2) are eliminated during the Scholl reaction, a new SOLO mode appears at 871 cm^−1^ (pink) for **wGNR**. Various DUO‐modes can be found around 808 cm^−1^ (orange). Vibrations related to the benzene ring adjacent to the octagon (QUATRO) can be assigned to the peaks at 748 cm^−1^ and 719 cm^−1^ (blue), respectively. A more detailed list of vibrations and their assignments can be found in the Supporting Information Chapter 6.

The experimental Raman spectra of **LTP** and **wGNR** have been compared with results from periodic‐boundary‐conditions DFT calculations run at the B3LYP/6‐31G(d,p) level using the CRYSTAL17 code.^[47]^ Details about Raman experiments and the setup of the calculation are provided in the Supporting Information Chapters 1 and 5. The observed Raman spectra exhibited an excellent match against their simulations (Figures 4b, c). The typical D and G bands are found for **LTP** at 1334 cm^−1^ and 1606 cm^−1^, respectively. In the case of **wGNR**, a line broadening, which is also predicted by DFT, is observed. By comparison with the DFT result, the D band is assigned to the shoulder at 1297 cm^−1^. The main peak observed at 1335 cm^−1^ is assigned to CC scaffold stretching, and the G band consists of several vibrational modes around 1608 cm^−1^. A detailed assignment of the Raman spectra of **LTP** and **wGNR** can be found in the Supporting Information Chapter 5.

The absorption and emission spectra of compounds **7**, **1**, **LTP**, and **wGNR** in chloroform are illustrated in Figure 4d. Due to their weak conjugation and similar structure, **7** and **LTP** each display only a single high energy absorption at 324 nm and 313 nm, and similar emission at 420 nm and 425 nm, respectively. Compared to **7**, model compound **1** exhibits three red‐shifted absorption bands at 348 nm, 388 nm, and 403 nm with an emission maximum at 477 nm and a shoulder at 503 nm. In comparison, **wGNR** displays the largest red shift in absorption (391 nm, 414 nm) and emission (558 nm) of the investigated compounds due to the extended π‐conjugation. Moreover, the band structure calculation estimates **wGNR** to possess a band gap of 2.18 eV, matching perfectly with the value of 2.16 eV derived from a tauc‐plot (SI Figure S2).

### wGNR in Organic Light‐Emitting Cells (OLECs)

**Figure 5:**
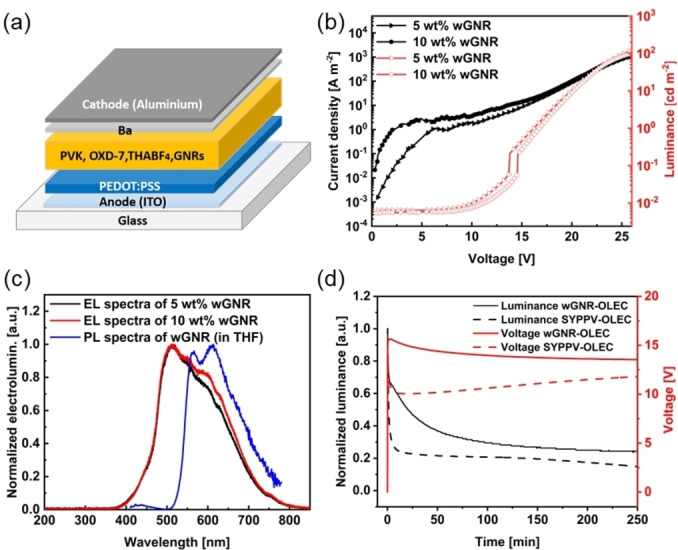
GOLEC fabrication with **wGNR** as emissive layer. (a) Schematic device architecture for the GOLECs; (b) current density and luminance against voltage for GOLECs with various weight fractions of **wGNR**; (c) EL spectra for GOLECs with 5 wt % and 10 wt % **wGNR** and PL spectra of **wGNR** in solution with THF as solvent; (d) luminance and voltage against time for GOLECs with 5 wt % **wGNR** with a current density at 100 A m^−2^.

Due to its unique topological structure containing 8‐membered rings, which yields high dispersibility and emissive features, **wGNR** emerges as a promising emissive material in opto‐electronic devices. Compared to small molecular emitters, better photostability has been reported for nanographenes or nanoribbons in light‐emitting devices.^[48]^ Moreover, OLECs based on thick active layers with conductive salts make them more fault‐tolerable compared to OLEDs.^[49]^ Here, we demonstrate the first GNR‐based organic light‐emitting electrochemical cells (GOLEC). GOLEC devices with the following structure were constructed: ITO (indium tin oxide)/PEDOT: PSS (45 nm)/PVK (polyvinylcarbazole): OXD‐7 (1,3‐bis[2‐(4‐tert‐butylphenyl)‐1,3,4‐oxadiazo‐5‐yl]benzene): THABF_4_ (tetrahexylammonium tetrafluoroborate): **wGNR** (100 nm) / Ba (10 nm)/Al (100 nm), as shown in Figure 5a. The energy diagram for the GOLEC devices is shown in Supporting Information Figure S56. In this device configuration, the ITO and PEDOT: PSS layer works as the anode, while the barium/aluminum layer works as the cathode. The THABF_4_ serves as the electrolyte to create electrochemical driving conditions with an efficient and balanced p‐/n‐doping capability, making efficient charge injection/transport for holes and electrons possible. The PVK and OXD‐7 hosts have two functions here: fixing the **wGNR** to reduce aggregation and therefore giving a smooth surface, and enhancing the charge carrier injection into the emissive layer. The morphology of the emissive layers with or without the emissive GNRs has been investigated by atomic force microscopy (AFM, Supporting Information Figure S55).

Compared with the film with only PVK: OXD‐7 host, the roughness of the film containing **wGNR** is almost identical, with RMS roughness as low as 0.6 nm. It demonstrates that a small fraction of **wGNR** in the polymer hosts has minor impacts on the film morphology, and therefore smooth interfaces are maintained. The current density‐voltage‐luminance characteristics are plotted in Figure 5b for GOLECs with two different **wGNR** concentrations. For comparison, the performance of reference devices without **wGNR** is presented in Supporting Information Figure S57. A maximum luminance of about 120 cd m^−2^ can be achieved. When increasing the weight fraction of **wGNR** from 5 wt % to 10 wt %, there is only a minor difference in the current density and luminance. To confirm the device reproducibility, we compared five devices constructed with 5 wt % **wGNR** (for current density‐voltage‐luminance characteristics, see Supporting Information Figure S58). The nearly identical current density and luminance behavior under different driving voltages demonstrate good reproducibility of GOLECs based on **wGNR**. The normalized EL spectra of the GOLECs with **wGNR** and PL spectrum in THF solution are shown in Figure 5c. When increasing the weight ratio of the **wGNR**, the shape of the EL spectra slightly changes, specifically increasing the shoulder peak at ca. 600 nm, which corresponds to the photoluminescence peak of **wGNR** in THF. The difference between the EL and PL spectra might result from the cavity resonance effect.^[50]^

The EL spectra of GOLECs under different biases are presented in Supporting Information Figure S59. The EL intensity increases significantly when enhancing the driving voltage, but the EL spectral lineshapes are almost identical with negligible voltage dependence. Nevertheless, compared to the EL spectrum of the reference devices without **wGNR**, there is a clear difference, as shown in Supporting Information Figure S57b.

For GOLECs, under an external bias, ions are redistributed in the emissive layer. Anions and cations migrate to anode and cathode respectively, forming electric double layers (EDLs).[^51^, ^52^] The formed EDLs decrease the width of the potential barriers and enhance the tunneling injection of electrons and holes, leading to an increase in current density. These injected carriers then transport through the active layer and recombine, with the recombined excitons contributing to the electroluminescence. This process can be detected by operating the device under constant current, with the time to obtain luminescence as a measure of the device response speed. Figure 5d shows the luminance intensity and voltage versus time with a constant current density of 100 A m^−2^. There is a rapid increase of electroluminescence right after the current injection, showing a short turn‐on time of less than 10 seconds. The fast turn‐on and then moderate decrease of voltage indicates a fast redistribution of ions and the formation of EDLs.^[53]^ The turn‐on time of GOLECs developed in this work is comparable to OLECs based on the classic super yellow poly(*p*‐phenylene vinylene) (SYPPV) polymer, as shown in Figure 5d.

It is worth noting that the GOLECs can still maintain 25 % of the maximum electroluminescence after the continuous operation of 4 hours. Compared to OLECs based on SYPPV, the operation lifetime is much longer, as shown in Figure 5d. Furthermore, it is noted that OLECs based on **wGNR** exhibit superior operational stability compared to devices based on thermally activated delayed fluorescence emitters.^[54]^ Such fast responding OLECs with remarkable stabilities can be treated as a starting point for future device development based on **wGNR**, considering their good solubility, the feasibility of chemical structure tailoring, and the unique optical properties demonstrated in this work.

## Conclusion

In summary, we have successfully demonstrated the first solution synthesis of a wavy GNR (**wGNR**) with periodic octagons in its backbone. The **wGNR** was obtained by selective cyclodehydrogenation of its well‐designed ladder‐type polymer (**LTP**) precursor and exhibited a unique wave‐shaped geometry, as further demonstrated by the single‐crystal structure of model compound **1**. This distinctive non‐planar geometry gives **wGNR** excellent dispersibility in common organic solvents such as chloroform (ca. 10 mg mL^−1^), and unlocks emissive features that are usually suppressed by strong π–π interactions in solution‐synthesized GNRs. The resultant **wGNR** has been thoroughly investigated by solid‐state NMR, UV/Vis, Raman, and FT‐IR spectroscopy as well as by DFT simulations to compare to the experimentally obtained spectra. Utilizing the emissive characteristic of **wGNR**, GNR‐based organic light‐emitting cells have been fabricated and investigated for the first time, with fast response and short turn‐on times (<10 s). These devices exhibited a peak luminance of 120 cd m^−2^ with high stability (4 hours with 25 % of the initial peak electroluminescence). This advancement marks a significant starting point for utilizing GNRs in optoelectronic devices, paving the way for the development of tunable multi‐tasking materials for light‐emitting devices. By further tuning and functionalizing the backbone of non‐benzenoid GNRs, it becomes possible to precisely adjust charge transport, stability, and photoluminescence, thus expanding the potential for GNR‐based materials in various semiconductor devices.

## Supporting Information

The authors have cited additional references within the Supporting Information.[^55^, ^56^, ^57^, ^58^, ^59^, ^60^, ^61^, ^62^, ^63^, ^64^, ^65^, ^66^]

## Conflict of Interests

The authors declare no conflict of interest.

1

## Supporting information

As a service to our authors and readers, this journal provides supporting information supplied by the authors. Such materials are peer reviewed and may be re‐organized for online delivery, but are not copy‐edited or typeset. Technical support issues arising from supporting information (other than missing files) should be addressed to the authors.

Supporting Information

## Data Availability

The data that support the findings of this study are available from the corresponding author upon reasonable request.
