# Supplementary material for: Wavy Graphene Nanoribbons Containing Periodic Eight‐Membered Rings for Light‐Emitting Electrochemical Cells
Source: Angew Chem Int Ed Engl. 2024 Oct 31;63(50):e202415670. doi: 10.1002/anie.202415670 (PMC11609969; doi:10.1002/anie.202415670)
Supplement: Supplementary file 1 — Supporting Information [file ANIE-63-e202415670-s001.pdf]

Angewandte Chemie
Eine Zeitschrift der Gesellschaft Deutscher Chemiker

Supporting Information

Wavy Graphene Nanoribbons Containing Periodic Eight-Membered Rings for Light-Emitting Electrochemical Cells

S. Obermann, X. Zhou, L. A. Guerrero-León, G. Serra, S. Böckmann, Y. Fu, E. Dmitrieva, J.-J. Zhang, F. Liu, A. A. Popov, A. Lucotti, M. R. Hansen, M. Tommasini, Y. Li, P. W. M. Blom, J. Ma*, X. Feng**

Supporting Information

Wavy Graphene Nanoribbons Containing Periodic Eight-Membered Rings for Light-Emitting Electrochemical Cells

Sebastian Obermann,#[a] Xin Zhou,#[b] L. Andrés Guerrero-León,[a] Gianluca Serra,[c] Steffen Böckmann,[d] Yubin Fu,[a] Evgenia Dmitrieva,[e] Jin-Jiang Zhang,[g] Fupin Liu,[e] Alexey A. Popov,[e] Andrea Lucotti,[c] Michael Ryan Hansen,[d] Matteo Tommasini,[c] Yungui Li,*[b] Paul W. M. Blom,[b] Ji Ma,*[a,f] Xinliang Feng*[a, g]

[a] Center for Advancing Electronics Dresden (cfaed) & Faculty of Chemistry and Food Chemistry, Technische Universität Dresden, Mommsenstrasse 4, 01069 Dresden, Germany

[b] Max-Planck-Institute for Polymer Research, Ackermannweg 10, 55128 Mainz, Germany

[c] Department of Chemistry, Materials, Chemical Engineering, Politecnico di Milano, Piazza Leonardo da Vinci 32, 20133 Milano, Italy

[d] Institute of Physical Chemistry, University of Münster, 48149 Münster, Germany

[e] Leibniz Institute for Solid State and Materials Research, Helmholtzstr. 20, 01069 Dresden, Germany

[f] College of Materials Science and Opto-Electronic Technology & Center of Materials Science and Optoelectronics Engineering, University of Chinese Academy of Science, 100049 Beijing, P. R. China

[g] Max-Planck-Institute of Microstructure Physics, Weinberg 2, 06120 Halle, Germany

# These authors contributed equally to the work

Table of Contents

1 Experimental section S4

1.1 General methods and materials S4

1.2 Synthetic procedures and characterization data S7

1.2.1 2-((phenylsulfonyl)methyl)benzonitrile (S1) S7

1.2.2 2-((phenylsulfonyl)methyl)benzaldehyde (S2) S8

1.2.3 Acetylene building block 6 .... S9

1.2.4 2,7-di-*tert*-butylpyrene (2) S10

1.2.5 2,7-di-*tert*-butylpyrene-4,5-dione (3) S11

1.2.6 1,3-bis(4-(*tert*-butyl)phenyl)propan-2-one (4) S12

1.2.7 Cyclopentadienone 5 .... S13

1.2.8 Model compound precursor 7 .... S14

1.2.9 Model compound 1 .... S15

1.2.10 1,3-bis(4-bromophenyl)propan-2-one S4 .... S16

1.2.11 1,3-bis(4-dodecylphenyl)propan-2-one S5 .... S17

1.2.12 2,7-di-*tert*-butylpyrene-4,5,9,10-tetraone (S6) S19

1.2.13 Cyclopentadienone 8 .... S20

1.2.14 Ladder type Polymer LTP S21

1.2.15 wGNR S22

2 NMR Spectra S23

2.1 2-((phenylsulfonyl)methyl)benzonitrile (S1) S23

2.2 2-((phenylsulfonyl)methyl)benzaldehyde (S2) S24

2.3 Acetylene building block 6 .... S25

2.4 2,7-di-*tert*-butylpyrene (2) S26

2.5 2,7-di-*tert*-butylpyrene-4,5-dione (3) S27

2.6 1,3-bis(4-(*tert*-butyl)phenyl)propan-2-one (4) S28

2.7 Cyclopentadienone 5 .... S29

2.8 Model compound precursor 7 .... S30

2.9 Model compound 1 .... S33

2.10 1,3-bis(4-bromophenyl)propan-2-one S4 .... S36

2.11 1,3-bis(4-dodecylphenyl)propan-2-one S5 .... S37

2.12 2,7-di-*tert*-butylpyrene-4,5,9,10-tetraone (S6) S38

2.13 Cyclopentadienone 8 .... S39

2.14 LTP and wGNR S40

3 CV/SEC of compound 1 .... S41

4 Analytical Gel Permeation Chromatography (GPC) of LTP S43

5 Raman spectroscopy of LTP and wGNR S45

6 FT-IR spectroscopy of 7, 1, LTP and wGNR S52

8 X-Ray crystallographic analysis S56

8.1 Compound 7 .... S56

8.2 Compound 1 .... S58

9 DFT Calculation Details S60

9.1 Simulation of the infrared spectra S60

9.2 Band gap and Uv-Vis calculation S61

10 GOLEC fabrication S62

11 Solid state NMR S65

12 References S70

1 Experimental section

1.1 General methods and materials

All the reagents were obtained from Sigma Aldrich, TCI, abcr, Alfa Aesar, Strem, Fluorochem, and chemPUR. All these chemicals were used as received without further purification. All reactions dealing with air- or moisture-sensitive compounds were carried out in a dry reaction vessel under an argon (Ar) atmosphere using standard vacuum-line and Schlenk techniques. Anhydrous dichloromethane, THF, and toluene were obtained from an MBRAUN MB-SPS-5 solvent purification system.

Thin layer chromatography (TLC) was performed on silica-coated aluminum sheets with a fluorescence indicator (TLC silica gel 60 F254, purchased from Merck KGaA).

Column chromatography was performed on silica (SiO_2, particle size 0.063 - 0.200 mm, purchased from VWR).

Nuclear magnetic resonance (NMR) spectra were recorded on Bruker AVANCE-II 300 and Bruker Avance III HD 300 spectrometers (300 MHz and 75.5 MHz for ^{1}H and ^{13}C respectively) and on a Bruker DRX 500 (500 MHz and 125.7 MHz for ^{1}H and ^{13}C respectively) using a 5 mm ^{1}H/^{13}C gradient probe at room temperature. CD_2Cl_2 (δ (^{13}C) = 53.84 ppm) or $CDCl_3$ (δ (^{13}C) = 77.16 ppm) were used as solvent, lock and internal standard for ^{13}C measurements. The ^{1}H-spectra were referenced to internal TMS standards at 0.00 ppm. If not present, the solvent residual signals of DCM (δ (^{1}H) = 5.32 ppm) and $CHCl_3$ (δ (^{1}H) = 7.26 ppm) were used for referencing. The 2D NMR spectra were recorded using the standard pulse sequences of the Bruker software package (TOPSPIN 3.6). The sample temperature was controlled by the Bruker variable temperature accessory BVT-3000.

The mass spectrometry analysis was performed on a Bruker Autoflex Speed MALDI-TOF MS (Bruker Daltonics, Bremen, Germany) using DCTB (*trans*-2-[3-(4-*tert*-butylphenyl)-2-methyl-2-propenylidene]malononitrile) as matrix.

High-Resolution Atmospheric Pressure Chemical Ionization (APCI) mass spectra were recorded with Agilent 6538 Ultra High Definition (UHD) Accurate-Mass Q-TOF LC/MC system, using the positive mode.

UV-Visible spectra were measured on an Agilent Cary 5000 UV-Vis-NIR spectrophotometer by using 10 mm optical-path quartz cell at room temperature.

Photoluminescence spectra were measured on PerkinElmer fluorescence spectrometer LS 55.

Recycling gel permeation chromatography (rGPC) was carried out on a Japan Analytical Industry JAI-HPLC LC 9110 II Next equipped with a JAIGEL-2HH and a JAIGEL-1HH column. Chloroform was used as the eluent in a flow rate of 5 mL/min.

Relative molar masses were determined by gel permeation chromatography (GPC) with an Agilent Technologies 1260 Infinity LC system equipped with two Resipore columns and RI and UV–Vis detection. Chloroform was used as eluent with a flow rate of 1 mL min^{-1}. The measurements were carried out at 40 ºC. The molar masses were calculated relative to polystyrene standards with low dispersity.

The FT-Raman spectrum of **LTP** reported in Figure 4b was recorded using a Nicolet NXR9650 FT-Raman (Thermo-Nicolet) equipped with an InGaAs detector and a Nd-YVO4 laser providing a 1064 nm excitation line. The FT-Raman spectrum was acquired in backscattering geometry with a laser spot diameter of approximately 50 μm, a resolution of 4 cm^{-1}, and a power at the sample of approximately 2 W. The sample was stable under this illumination condition due to the relatively large laser spot size and the absence of electronic absorption at this wavelength. Due to the high fluorescence observed in the Raman spectrum of **wGNR** with the 1064 nm excitation line, we decided to use the 405 nm excitation line for this sample. The micro-Raman spectra of **wGNR** were obtained with the LABRAM HR800 instrument (Horiba Jobyn-Yvon) using a 50X objective (0.75 NA) and the 405 nm excitation provided by a solid-state laser produced by Integrated Optics. The Raman spectrum of **wGNR** reported in Figure 4c was collected in backscattering geometry with a laser spot diameter of approximately 1 μm and a power at the sample of about 0.2 mW.

FT-IR spectra were collected using a Tensor II from Bruker with an attenuated total reflection (ATR) unit.

Cyclic voltammetry (CV) was carried out on a PARSTAT4000 potentiostat (Princeton Applied Research, Ametek, Germany) in a three-electrode cell in degassed dry dichloromethane or tetrahydrofuran solution containing 0.1 M of tetra-*n*-butylammonium hexafluorophosphate (n-Bu_4NPF_6) as a supporting electrolyte. A Pt disc, AgCl-coated silver wire, and Pt sheet electrode were used as the working electrode, the reference electrode, and the counter electrode, respectively. All

potentials are given versus ferrocene/ferrocenium (Fc/Fc^+) redox couple as an internal standard. The electrochemical measurements were performed under inert (nitrogen) atmosphere and at ambient temperature.

For EPR measurements, an EMX X-band CW spectrometer (Bruker BioSpin, Germany) at 100 kHz modulation was used. The spectra were recorded in an optical EPR cavity (Bruker, Germany) allowing the connection of two optical waveguides. Thus, the electronic absorption spectra in transmission mode are measured simultaneously with the EPR spectra. For precise determination of *g* values, an NMR teslameter (ER036TM, Bruker Biospin GmbH) was used. For UV–vis–NIR measurements, the spectrometers AvaSpec-2048x14-USB2 with the CCD detector and AvaSpec-NIR256-2.2 with the InGaAs detector (Avantes, The Netherlands) applying the AvaSoft8 software were used. The *in situ* spectroelectrochemical measurements were controlled by a PG 390 potentiostat/galvanostat (HEKA Elektronik GmbH, Germany) equipped with the PotMaster v2x80 software and triggering both the EPR and the UV–vis–NIR spectrometer modules. The experiments were carried out in an EPR flat cell with a three-electrode arrangement consisting of a laminated gold μ-mesh (Goodfellow, UK) as working electrode, an AgCl-coated silver wire as reference electrode, and a platinum wire as counter electrode. The cell assembling was done under an inert (nitrogen) atmosphere. The spectra were collected during the cyclic voltammogram (CV), the latter was recorded at a continuous slow scan rate. Each UV−Vis−NIR spectrum was collected relative to that of the neutral (uncharged) compound.

1.2 Synthetic procedures and characterization data

1.2.1 2-((phenylsulfonyl)methyl)benzonitrile (S1)

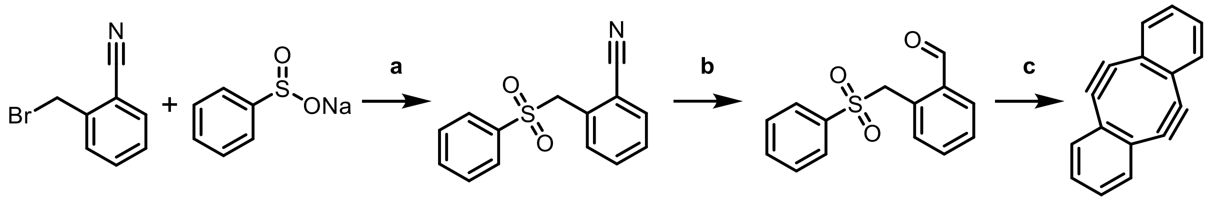

S1 **S2** **6**

Scheme S1. Synthesis of building block **6**. Reagents and conditions: a) DMF, 80 °C, overnight; b) DIBAL-H, DCM, -78 °C, 2 h; c) i) $ClPO(OEt)_2$, LiHMDS, -78 °C to rt, 2 h; ii) LDA, -78 °C, 2 h.

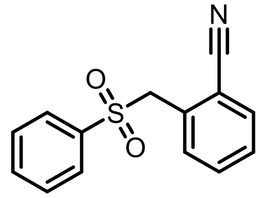

S1

To a 25 mL Schlenk flask, 2-brommethyl-benzonitril (4.00 g, 20.4 mmol) and sodiumbenzenesulfinate (4.9 g, 24.48 mmol) were added. After purging the flask with argon three times, 7 mL of DMF was added. The reaction mixture was allowed to stir at 80 °C overnight. After cooling to room temperature, water was added. The aqueous layer was extracted three times with DCM and dried over $MgSO_4$. The volatiles were evaporated, and the residue was redissolved in a small amount of DCM. 250 mL of iso-hexane was added, and colorless crystals began to grow quickly. After 15 minutes, the mixture was filtrated, and carefully washed with iso-hexane, yielding compound **S1** as colorless needles in a yield of 85% (4.45 g, 17.29 mmol).

^{1}H NMR (300 MHz, CD_2Cl_2) δ 7.78 – 7.57 (m, 5H), 7.57 – 7.43 (m, 4H), 4.56 (s, 2H).

^{13}C NMR (76 MHz, CD_2Cl_2) δ 138.2, 134.7, 133.4, 133.3, 132.5, 132.1, 129.8, 129.7, 129.0, 117.1, 114.9, 60.9.

HR-MS (APCI), m/z: $[M]^+$ calculated for $C_{14}H_{11}NO_2S$ = 257.05105, exp. m/z = 257.05112, error = +0.29 ppm.

1.2.2 2-((phenylsulfonyl)methyl)benzaldehyde (S2)

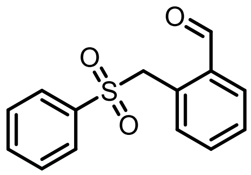

S2

To a flame dried 100 mL Schlenk flask, compound **S1** (1.00 g, 3.89 mmol) was added. After purging the flask with argon three times, 50 mL of dry DCM were added and the solution was cooled to -78 °C with an acetone/dry ice bath. DIBAL-H (1 M in hexane, 8.94 mmol) was added dropwise and the mixture was allowed to stir for 2 hours at -78 °C. The cooling was removed and the reaction mixture was quenched with 20 mL of saturated NH_4Cl-solution. 20 mL of 2 M aqueous HCl were added, and the aqueous layer was extracted three times with DCM, dried over $MgSO_4$ and the volatiles were evaporated. The crude product was filtered over a plug of silica with DCM as the eluent, yielding compound **S2** as a grey solid in 52% yield (525 mg, 2.02 mmol).

^{1}H NMR (300 MHz, CD_2Cl_2) δ 9.80 (s, 1H), 7.73 – 7.64 (m, 1H), 7.61 – 7.45 (m, 5H), 7.44 – 7.33 (m, 2H), 7.30 – 7.19 (m, 1H), 4.90 (s, 2H).

^{13}C NMR (76 MHz, CD_2Cl_2) δ 191.9, 138.4, 134.9, 133.9, 133.9, 133.7, 133.4, 129.5, 129.0, 128.9, 128.5, 57.7.

HR-MS (APCI), m/z: $[M]^+$ calculated for $C_{14}H_{12}O_3S$ = 260.05071, exp. m/z = 260.05063, error = -0.31 ppm.

1.2.3 Acetylene building block 6

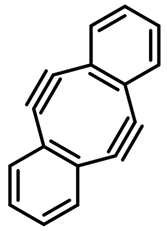

6

To a flame dried 100 mL Schlenk flask, aldehyde **S2** (300 mg, 1.15 mmol) was added, and the flask was purged with argon three times. Dry THF (34 mL) and diethyl phosphorochloridate (0.2 mL, 1.38 mmol) were added. The mixture was then cooled to -78 °C via acetone/dry ice and LiHMDS (1.77 mL, 2.30 mmol) was added dropwise. The cooling was removed after 30 minutes of stirring, and the reaction mixture was stirring at room temperature for another 90 minutes. Meanwhile, a fresh LDA-solution was prepared: to a flame dried 50 mL Schlenk flask, 18 mL of dry THF and diisopropylamine (1.71 mL, 12.10 mmol) were added. The solution was cooled to -78 °C and *n*-buthyllithium (1.6 M in hexane, 7.20 mL, 11.52 mmol) was added dropwise. The mixture was allowed to stir for 90 minutes at -78 °C. After passing the above-mentioned time at room temperature, the reaction mixture was cooled back to -78 °C and the freshly prepared LDA-solution was added dropwise. After stirring the reaction mixture for 2 hours, the reaction was quenched by addition of 10 mL of saturated NH_4Cl-solution. Water and DCM were added, and the aqueous layer was extracted three times with DCM and subsequently dried over $MgSO_4$. Flash column chromatography over silica with 3:1 *iso*-hexane/DCM provided the yellow product **6** in 61% yield (70 mg, 0.35 mmol).

^{1}H NMR (300 MHz, $CDCl_3$) δ 6.93 – 6.78 (m, 4H), 6.75 – 6.59 (m, 4H).

^{13}C NMR (76 MHz, $CDCl_3$) δ 133.3, 129.4, 127.3, 109.7.

ESI-, APCI-, and MALDI-TOF-MS failed to yield the molecule peak for this compound.

The molecule is known to the literature and the NMR spectra match the reported ones.[1,2]

1.2.4 2,7-di-*tert*-butylpyrene (2)

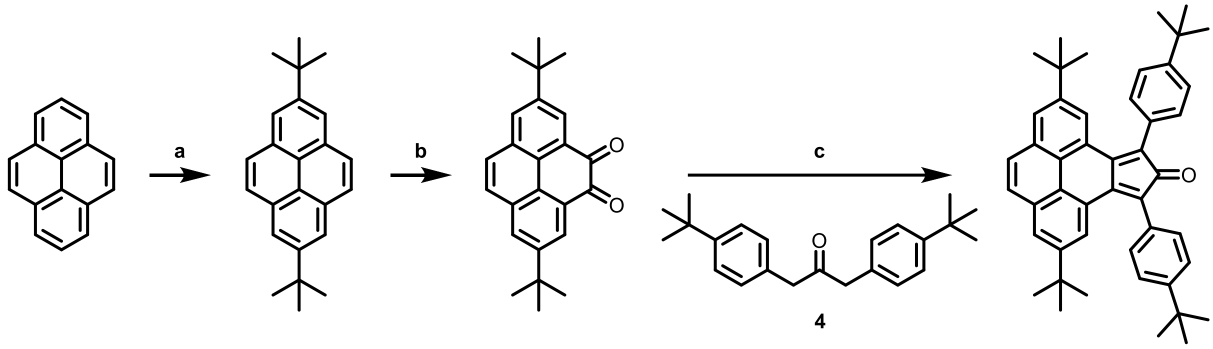

2 3 5

Scheme S2. Synthesis of cyclopentadienone **5**. Reagents and conditions: a) 2-chloro-2-methylpropane, $AlCl_3$, DCM, 0°C, overnight; b) $RuCl_3 \cdot xH_2O$, $NaIO_4$, DCM, MeCN, H_2O, rt, 1.5 h; c) 1,3-bis(4-*tert*-butylphenyl)propanone (**4**), KOH, MeOH, 80 °C, 1 h.

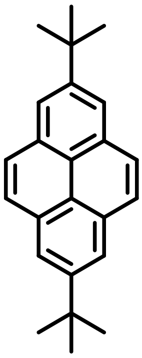

2

In a dry 250 mL round bottom flask, 10 g (49.44 mmol) of pyrene and anhydrous $AlCl_3$ (1.32 g, 9.89 mmol) were dissolved in dry DCM (100 mL). The solution was cooled to 0 °C and a solution of 2,2-dimethylchloropropane (20.60 g, 222.49 mmol) in 15 mL of dry DCM was added dropwise while stirring the reaction mixture. The black slurry was allowed to warm up to room temperature overnight. As the retention indices of starting material, single alkylation and double alkylation products are almost identical, complete conversion of the starting material was checked by NMR. 0.5 mL of reaction mixture were taken, diluted in *iso*-hexane and filtered over silica. After evaporation of the volatiles and redissolvation in deuterated DCM, the ^{1}H-NMR spectrum was checked. After confirming that only the target was present, the reaction mixture was transferred to a separating funnel, water was carefully added and the mixture was washed, with subsequent threefold extraction of the aqueous layer by DCM. The combined organic layers were dried over $MgSO_4$, filtrated, and the volatiles were evaporated. Silica gel column chromatography with pure *iso*-hexane to *iso*-hexane/DCM (9:1) afforded the target **2** in 74% yield (11.5 g, 36.57 mmol).

^{1}H NMR (300 MHz, CD_2Cl_2) δ 8.13 (s, 4H), 7.95 (s, 4H), 1.49 (s, 18H).

^{13}C NMR (76 MHz, CD_2Cl_2) δ 149.2, 131.2, 127.8, 123.1, 122.5, 35.5, 32.1.

HR-MS (APCI), m/z: calculated $[M]^+$ for $C_{24}H_{26}$ = 314.20345, exp. m/z = 314.20267, error = -2.5 ppm.

1.2.5 2,7-di-*tert*-butylpyrene-4,5-dione (3)

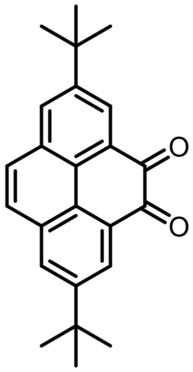

3

A 500 mL round bottom flask was charged with 2,7-di-*tert*-butylpyrene (**2**, 6.78 g, 21.56 mmol) and $RuCl_3 \cdot 3\ H_2O$ (564 mg, 2.16 mmol). DCM (120 mL), MeCN (120 mL) and water (160 mL) were added. To the stirring mixture, $NaIO_4$ (20.75 g, 97.02 mmol) was added in three portions over 10 minutes. After 90 minutes at room temperature, the mixture showed complete conversion of the starting material by TLC. Water and DCM were added, the layers were separated, and the aqueous layer was extracted three times with DCM. The combined organic layers were dried over $MgSO_4$, filtered, and the volatiles were evaporated. Column chromatography with silica gel (DCM/*iso*-hexane 2:1) afforded the title compound **3** in 47% yield (3.51 g, 10.19 mmol).

^{1}H NMR (300 MHz, CD_2Cl_2) δ 8.33 (d, J = 1.8 Hz, 2H), 7.98 (d, J = 1.8 Hz, 2H), 7.59 (s, 2H), 1.38 (s, 18H).

^{13}C NMR (76 MHz, CD_2Cl_2) δ 181.1, 151.4, 132.2, 132.1, 130.1, 128.2, 127.5, 126.6, 35.5, 31.3.

HR-MS (APCI), m/z: calculated $[M]^+$ for $C_{24}H_{24}O_2$ = 344.17763, exp. m/z = 344.17742, error = -0.6 ppm.

1.2.6 1,3-bis(4-(*tert*-butyl)phenyl)propan-2-one (4)

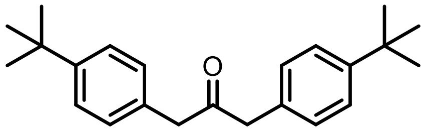

4

In a dry 1 L round bottom flask, *N,N'*-dicyclohexylcarbodiimide (26.83 g, 130.03 mmol) and DMAP (4.13 g, 33.81 mmol) were dissolved in dry DCM (300 mL) under argon atmosphere. 4-*tert*-butylphenylacetic acid (25.0 g, 130.03 mmol) in 400 mL dry DCM were added to this mixture dropwise via dropping funnel. The reaction mixture turned to a yellow/orange color immediately and was allowed to stir overnight at room temperature. Then, the formed solid was filtered, washed with DCM and the solid was discarded. After the volatiles were evaporated, silica gel column chromatography in *iso*-hexane/DCM (10:1 to 2:1) was performed. Final recrystallization from MeOH gave the product **4** in 46% yield (9.7 g, 30.08 mmol).

^{1}H NMR (300 MHz, CD_2Cl_2) δ 7.34 (d, *J* = 8.3 Hz, 4H), 7.08 (d, *J* = 8.3 Hz, 4H), 3.71 (s, 4H), 1.31 (s, 18H).

^{13}C NMR (76 MHz, CD_2Cl_2) δ 206.3, 150.3, 131.7, 129.6, 125.9, 49.0, 34.8, 31.5.

HR-MS (APCI), m/z: calculated $[M]^+$ for $C_{23}H_{30}O$ = 322.22967, exp. m/z = 322.22906, error = -1.87 ppm.

1.2.7 Cyclopentadienone 5

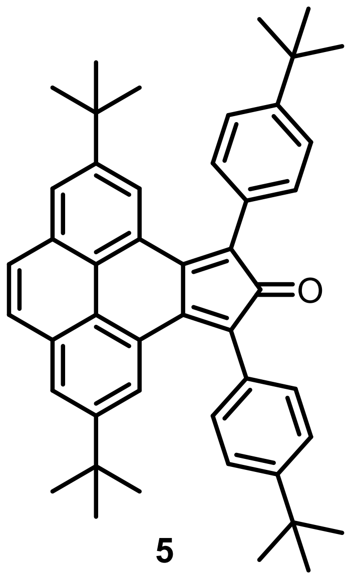

5

A dry 25 mL Schlenk tube was charged with diketone **3** (300.0 mg, 0.87 mmol) and carbonyle **4** (309.0 mg, 0.96 mmol). After purging with argon three times, EtOH (8 mL) was added. KOH (53.8 mg, 0.96 mmol) were added to the mixture. The flask was sealed and heated at 80 °C for 60 minutes. After cooling to room temperature, the mixture was filtrated and washed with small portions of chilled EtOH until the sticky mass became a powder. The hazelnut brown solid was used without further purification and presented itself in 61% yield (334 mg, 0.53 mmol).

^{1}H NMR (300 MHz, CD_2Cl_2) δ 7.71 (s, 2H), 7.59 (s, 2H), 7.52 (s, 2H), 7.46 (d, *J* = 8.2 Hz, 4H), 7.27 (d, *J* = 8.1 Hz, 4H), 1.30 (s, 18H), 1.01 (s, 18H).

^{13}C NMR (76 MHz, CD_2Cl_2) δ 201.2, 151.1, 149.7, 148.4, 132.0, 130.5, 129.5, 127.1, 127.0, 126.7, 126.5, 125.9, 124.7, 123.6, 34.6, 34.5, 31.0, 30.6.

HR-MS (MALDI-TOF), m/z: calculated $[M]^+$ for $C_{47}H_{50}O$ = 630.3856, exp. m/z = 630.384, error = -2.5 ppm.

1.2.8 Model compound precursor 7

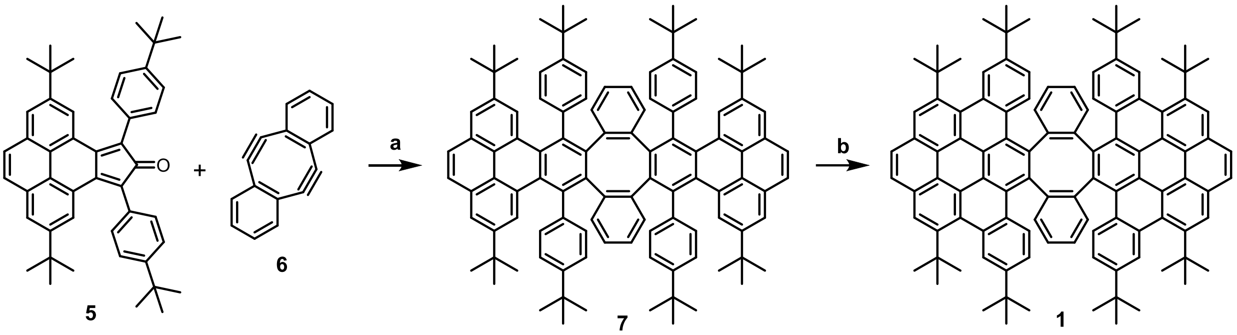

5 6 7 1

Scheme S3. Synthesis of model compound **1** from building blocks **5** and **6**. Reagents and conditions: a) PhMe, 110 °C, overnight; b) DDQ, DCM, TFA, -40 °C, 30 min.

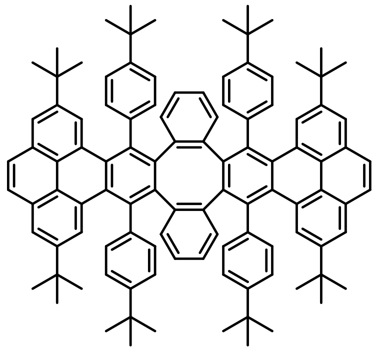

7

A dry 10 mL Schlenk tube was charged with acetylene building block **6** (15 mg, 75 µmol) and cyclopentadienone **5** (118 mg, 187 µmol). After purging with argon for three times, dry and degassed toluene (1 mL) was added, and the mixture was refluxed overnight. After confirming the complete consumption of the acetylene by TLC, the mixture was directly adsorbed on silica. Column chromatography with *iso*-hexane/DCM (5:1) afforded the target as a beige solid in 91% yield (96 mg, 68 µmol).

^{1}H NMR (300 MHz, CD_2Cl_2) δ 8.05 (s, 4H), 7.82 (s, 8H), 7.34 (d, *J* = 7.9 Hz, 4H), 7.28 (d, *J* = 8.1 Hz, 4H) 6.96 – 6.77 (m, 12H), 6.27 (d, J = 7.5 Hz, 4H), 1.33 (s, 36H), 1.12 (s, 36H).

^{13}C NMR (76 MHz, CD_2Cl_2) δ 149.1, 147.3, 141.6, 141.6, 140.6, 136.3, 133.6, 133.6, 132.8, 132.1, 130.4, 130.0, 127.3, 127.2, 126.0, 125.2, 125.1, 123.5, 121.8, 35.3, 34.8, 32.0, 31.9.

HR-MS (MALDI-TOF), m/z: calculated $[M]^+$ for $C_{108}H_{108}$ = 1404.845, exp. m/z = 1404.851, error = +4.3 ppm.

1.2.9 Model compound 1

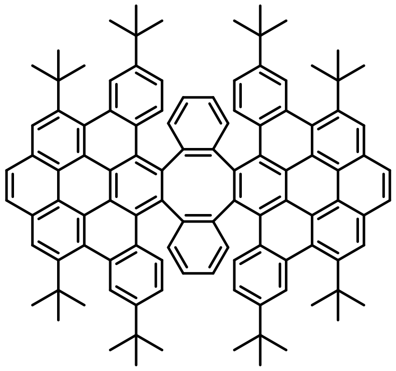

1

A dry 50 mL Schlenk flask was charged with precursor **7** (20.0 mg, 14.2 µmol) and DDQ (19.4 mg, 85 µmol). Dry and degassed DCM (20 mL) was added, and the mixture was cooled to -40 °C by a dry ice/MeCN mixture. Then, triflic acid (2 mL) was added dropwise to the reaction mixture. After 20 minutes, NEt_3 (3 mL) was added carefully to quench the reaction. Water was added and the layers were separated. The aqueous layer was extracted three times with DCM. The organic layers were combined and dried over $MgSO_4$, filtered and the volatiles were evaporated. Silica gel column chromatography with *iso*-hexane/DCM (5:1) as the eluent gave compound **1** as a yellow solid in 75% yield (15 mg, 11 µmol).

^{1}H NMR (300 MHz, CD_2Cl_2) δ 8.82 (s, 4H), 8.51 (d, *J* = 8.8 Hz, 4H), 8.37 (d, *J* = 2.1 Hz, 4H), 8.35 (s, 4H), 7.37 – 7.24 (m, *J* = 6.5, 4.0 Hz, 8H), 6.89 (dd, *J* = 8.8, 1.9 Hz, 4H), 1.68 (s, 36H), 1.40 (s, 36H).

^{13}C NMR (76 MHz, CD_2Cl_2) δ 145.9, 144.6, 144.6, 139.3, 133.8, 132.3, 130.5, 130.2, 128.6, 128.5, 128.1, 127.3, 127.2, 127.2, 126.7, 125.3, 124.3, 123.4, 120.9, 39.0, 35.3, 35.0, 31.8.

HR-MS (MALDI-TOF), m/z: calculated $[M]^+$ for $C_{108}H_{100}$ = 1396.782, exp. m/z = 1396.780, error = -1.4 ppm.

1.2.10 1,3-bis(4-bromophenyl)propan-2-one S4

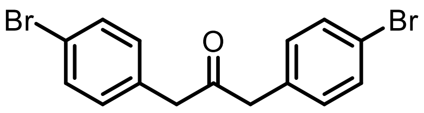

S4

A dry 500 mL three neck round bottom flask was charged DMAP (3.69 g, 30.23 mmol) and DCC (23.99 g, 116.25 mmol). Dry DCM (150 mL) was used to dissolve the solids. 4-bromophenylacetic acid (25.0 g, 116.25 mmol) was placed in a dropping funnel on top of the three-neck flask and the starting material was dissolved in 100 mL dry DCM. The mixture was added dropwise to the three-neck round bottom flask and stirred overnight at room temperature. Water was added and the layers were separated. The aqueous layer was washed three times with DCM, the organic layers were combined, dried over $MgSO_4$ and all volatiles were evaporated. The yellow orange residue was washed with hexane until colorless. No further purification was required according to the NMR spectra, and the product was obtained in 60% yield (12.8 g, 34.78 mmol).

^{1}H NMR (300 MHz, CD_2Cl_2) δ 7.57 – 7.43 (m, 4H), 7.15 – 7.02 (m, 4H), 3.75 (s, 4H).

^{13}C NMR (76 MHz, CD_2Cl_2) δ 204.0, 133.1, 131.6, 131.4, 120.9, 48.4.

HR-MS (APCI), m/z: calculated $[M]^+$ for $C_{15}H_{12}Br_2O$ = 365.92549, exp. m/z = 365.9241, error = -3.79 ppm.

1.2.11 1,3-bis(4-dodecylphenyl)propan-2-one S5

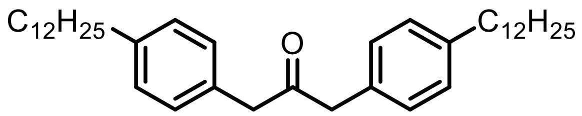

S5

A 250 mL Schlenk flask was charged with compound **S4** (12.8 g, 34.78 mmol), *p*-TsOH (0.6 g, 3.48 mmol) and the flask was flushed with argon three times. Then, ethylene glycol (19.45 mL, 347.8 mmol) and toluene (100 mL) were added, and the mixture was refluxed overnight. After cooling to room temperature, dichloromethane and water were added and the aqueous layer was extracted three times with dichloromethane. The organic layers were dried over $MgSO_4$, and all solvents were evaporated. Filtration over silica with isohexane/dichloromethane (1:1) as the eluent gave the colorless acetal, which was directly used for the next step.

A dried 500 mL two neck round bottle flask was charged with Mg turnings (9.91 g, 407.65 mmol) and dry THF (180 mL) was added after flushing with argon three times. A grain of iodine was added to activate the magnesium. After approx. 5 minutes, the reaction had ceased, and dodecyl bromide (29 g, 116 mmol) was added dropwise at room temperature under stirring. The mixture was stirred at 75 °C overnight, and another dried 500 mL round bottle flask was charged with the acetal of compound **S4** (12.0 g, 29.12 mmol), Pd(dppf)Cl_2 (1.43 g, 1.75 mmol), and dry THF (180 mL). While still warm, the Grignard solution was cannulated to the flask containing the starting material and catalyst, and the mixture was heated at 75 °C overnight. After cooling to room temperature, water and dichloromethane were added, the organic layer was extracted three times with dichloromethane and the combined organic layers were dried over $MgSO_4$. Silica gel column chromatography (isohexane/DCM 2:1) gave the acetal of compound **S5** with slight impurities in overstoichiometric yield (17.51 g, 112%). The ^{1}H-NMR spectrum was checked, then the ketone was deprotected.

^{1}H NMR (300 MHz, $CDCl_3$) δ 7.17 (d, J = 8.0 Hz, 4H), 7.07 (d, J = 8.1 Hz, 4H), 3.46 (s, 4H), 2.89 (s, 4H), 2.62 – 2.48 (m, 4H), 1.65 – 1.55 (m, 4H), 1.37 – 1.19 (m, J = 10.7, 3.8 Hz, 34H), 0.88 (t, J = 6.7 Hz, 8H).

A 1 L round bottle flask was charged with the crude product (16.0 g, 29.91 mmol), and flushed with argon three times. Then, THF (375 mL), conc. HCl (375 mL) and TFA (40 mL) were added, and the mixture was stirred at 50 °C for six hours. After cooling to room temperature, K_2CO_3 was added until the gas formation ceased and the THF was evaporated *in vacuo.* The mixture was extracted three times by dichloromethane,

the combined organic layers were dried over $MgSO_4$ and the solid was filtered. The filtrate was then concentrated and treated with an excess of MeOH and cooled by an ice bath, which resulted in the formation of a colorless precipitate. The precipitate was identified as the targeted compound **S5** (14.5 g, 29.54 mmol, 99% yield).

^{1}H NMR (300 MHz, CD_2Cl_2) δ 7.13 (d, J = 8.0 Hz, 1H), 7.04 (d, J = 8.0 Hz, 1H), 3.69 (s, 1H), 2.62 – 2.53 (m, 1H), 1.68 – 1.55 (m, 1H), 1.38 – 1.17 (m, 9H), 0.88 (t, J = 6.7 Hz, 2H).

^{13}C NMR (76 MHz, CD_2Cl_2) δ 206.3, 142.2, 131.9, 129.8, 129.0, 49.1, 35.9, 32.4, 32.0, 30.1, 30.1, 30.0, 29.9, 29.8, 23.1, 14.3.

HR-MS (APCI), m/z: calculated $[M]^+$ for $C_{39}H_{62}O$ = 546.48007, exp. m/z = 546.47763, error = -4.47 ppm.

1.2.12 2,7-di-*tert*-butylpyrene-4,5,9,10-tetraone (S6)

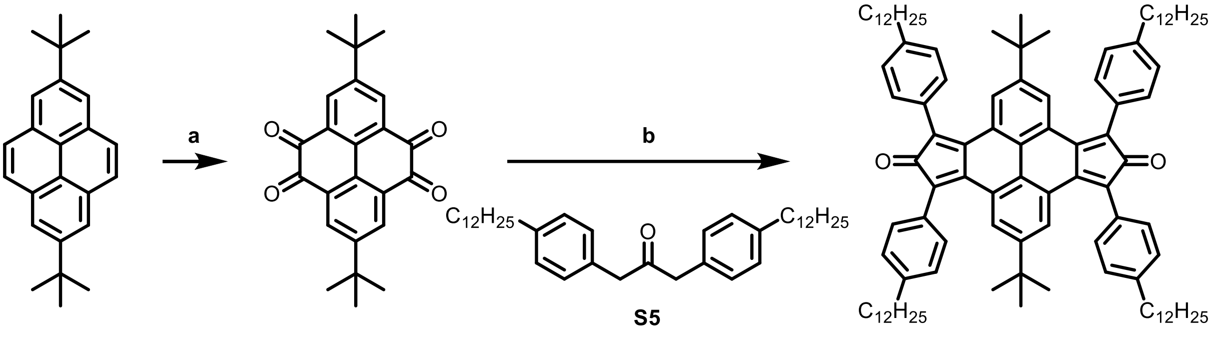

2 **S6** **8**

Scheme S4. Synthesis of cyclopentadiene **8**. Reagents and conditions: a) $RuCl_3$•xH_2O, $NaIO_4$, DCM/MeCN/H_2O, 40 °C, overnight; b) DBU, EtOH, 80 °C, 60 min.

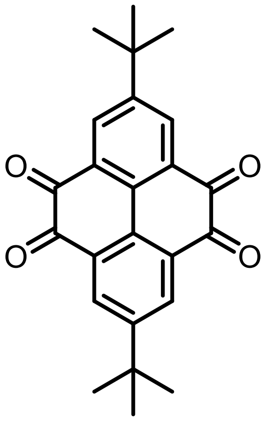

S6

To a 500 mL round bottom flask, 2,7-di-*tert*-butylpyrene (**2**, 3.00 g, 9.54 mmol), $RuCl_3$ • 3 H_2O (299 mg, 1.14 mmol), DCM (60 mL), MeCN (60 mL) and H_2O (75 mL) were added. While stirring, $NaIO_4$ (16.32 g, 76.32 mmol) was added in three portions over 10 minutes. After stirring at 40 °C overnight, the mixture was washed with saturated $Na_2S_2O_3$-solution, extracted three times with DCM and the combined organic layers were dried over $MgSO_4$. After evaporating the volatiles, the residue was adsorbed on silica gel and filtered over silica by DCM as the eluent. The product **S6** was obtained as an orange solid in 31 % yield (1.12 g, 2.99 mmol).

^{1}H NMR (300 MHz, CD_2Cl_2) δ 8.38 (s, 4H), 1.34 (s, 18H).

^{13}C NMR (76 MHz, CD_2Cl_2) δ 178.9, 155.1, 134.0, 132.7, 131.2, 35.7, 30.8.

HR-MS (APCI), m/z: calculated $[M]^+$ for $C_{24}H_{22}O_4$ = 374.15181, exp. m/z = 374.15209, error = +0.75 ppm.

1.2.13 Cyclopentadienone 8

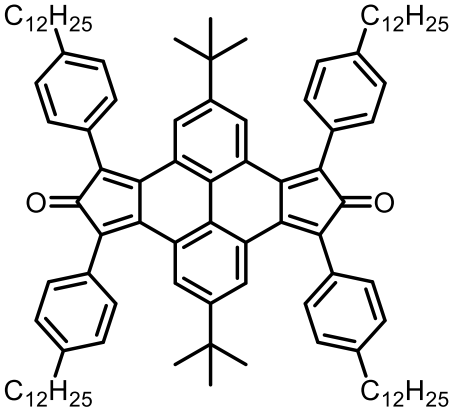

8

To a dry 100 mL Schlenk flask, compound **S6** (250 mg, 0.67 mmol) and compound **S5** (767 mg, 1.4 mmol) were added. After purging with argon three times, EtOH (50 mL) was added. To the mixture, DBU (386 mg, 2.54 mmol) was added dropwise. The mixture was heated to 80 °C for one hour. After cooling to room temperature, the mixture was filtered and washed successively with MeOH and EtOH. The black solid was collected and dried in a vacuum, affording compound **8** in 68% yield (630 mg, 0.45 mmol).

^{1}H NMR (300 MHz, CD_2Cl_2) δ 7.29 (s, 4H), 7.22 – 7.10 (m, *J* = 8.2 Hz, 16H), 2.55 (t, *J* = 7.5 Hz, 8H), 1.53 (s, 8H), 1.19 (s, 66H), 0.79 (t, *J* = 6.3 Hz, 18H), 0.58 (s, 18H).

^{13}C NMR (76 MHz, CD_2Cl_2) δ 200.8, 150.8, 147.4, 143.2, 129.9, 129.6, 128.9, 128.6, 127.5, 123.4, 35.8, 34.0, 31.9, 31.6, 29.7, 29.7, 29.6, 29.6, 29.6, 29.4, 29.3, 22.7, 13.9.

HR-MS (MALDI-TOF), m/z: calculated $[M]^+$ for $C_{102}H_{138}O_2$ = 1395.0691, exp. m/z = 1395.072, error = +2.1 ppm.

1.2.14 Ladder type Polymer LTP

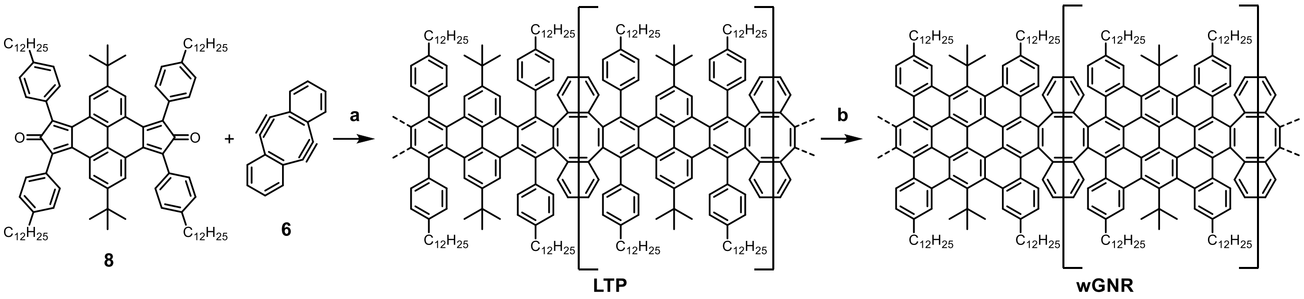

Scheme S5. Synthesis of graphene nanoribbon **wGNR**. Reagents and conditions: a) Ph_2O, 265 °C, 2 d; b) DCM, triflic acid, DDQ, -40 °C to rt, 3 d.

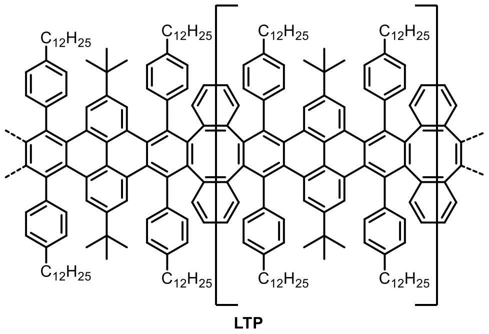

In a typical experiment, a flame dried 10 mL Schlenk tube was charged with compound **6** (10.0 mg, 49.94 µmol) and compound **8** (69.7 mg, 49.94 µmol). 0.25 mL dry and degassed diphenyl ether was added, and the tube was sealed and heated at 260 °C for three days. Upon cooling to room temperature, THF was added, and the mixture was filtered over silica (THF as eluent) and then reduced in volume *in vacuo*. Precipitation in Methanol yielded **LTP** as a brown solid in ~91% yield (70 mg). The polymer mixture was then subjected to recycling GPC and analytical GPC in chloroform (see Chapter 4).

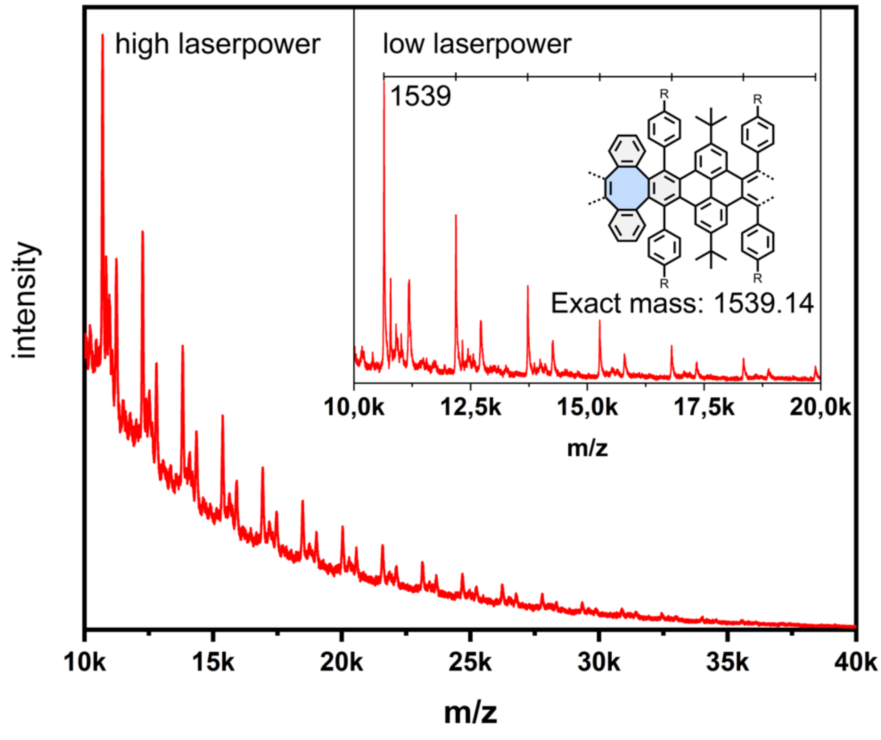

Figure S1. Linear mode MALDI-TOF of **LTP** with low and high laser strength, respectively.

1.2.15 wGNR

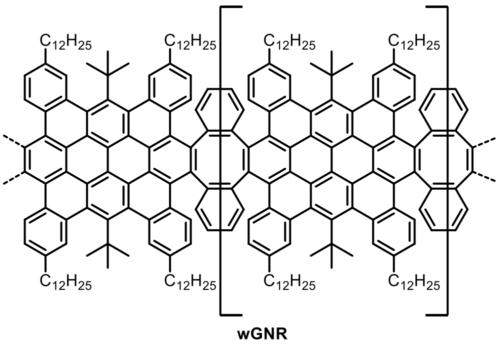

In a typical experiment, a flame-dried 10 mL Schlenk tube was charged with **LTP** (10 mg) and DDQ (8.7 mg, 38.25 µmol). After flushing with argon three times, dry and degassed dichloromethane (10 mL) was added to the mixture, and the reaction vessel was cooled to -40 °C by a MeCN/dry ice mixture. Then, triflic acid (1 mL) was added dropwise while the reaction mixture was slowly purged with argon. The mixture was allowed to warm up to room temperature in the ice bath and the argon purging was stopped after the first 24 hours. After three days, an excess of MeOH was added to the mixture to precipitate a black solid. After filtration and washing with MeOH, **wGNR** was obtained as a brown-black powder in 90% yield (9 mg).

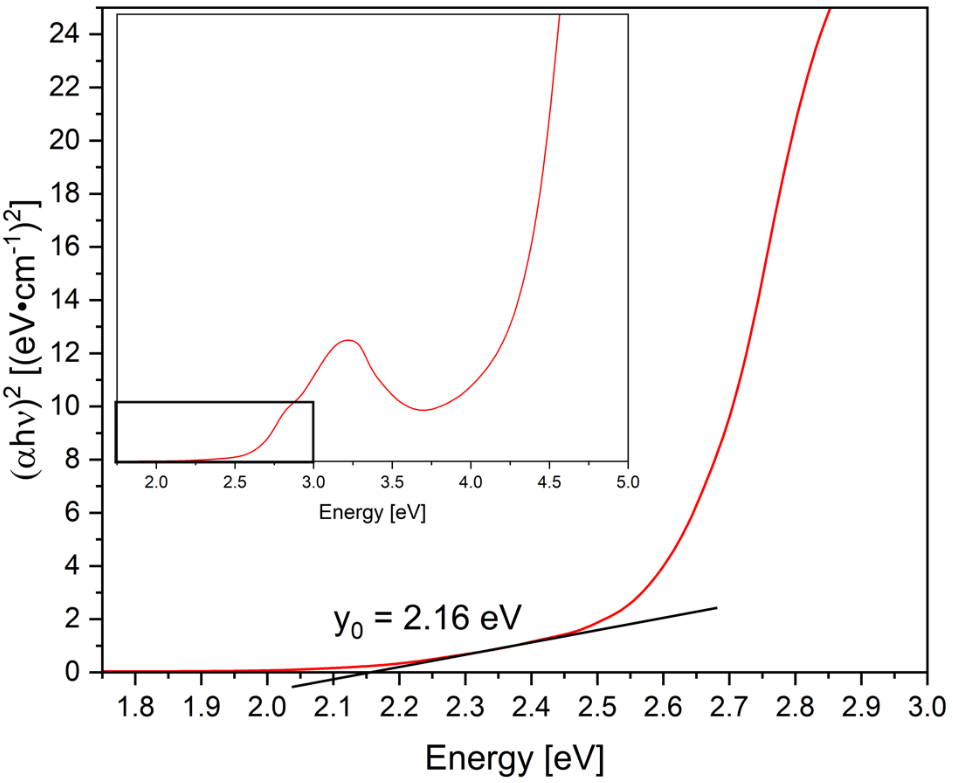

Figure S2. Tauc-Plot derived from the UV-Vis spectra of wGNR, showing an optical bandgap of ~2.16 eV.

2 NMR Spectra

2.1 2-((phenylsulfonyl)methyl)benzonitrile (S1)

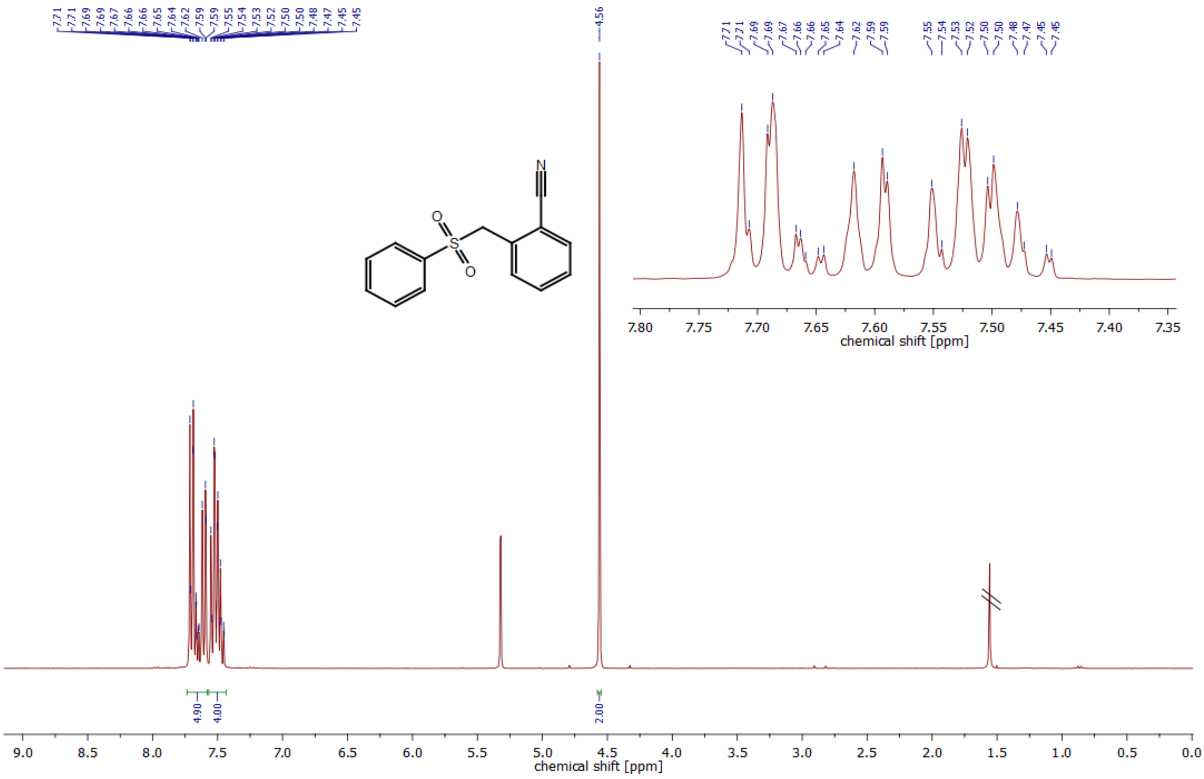

Figure S3. ^{1}H-NMR spectra of 2-((phenylsulfonyl)methyl)benzonitrile (**S1**, 300 MHz, $CDCl_3$). 1.55 ppm: H_2O.

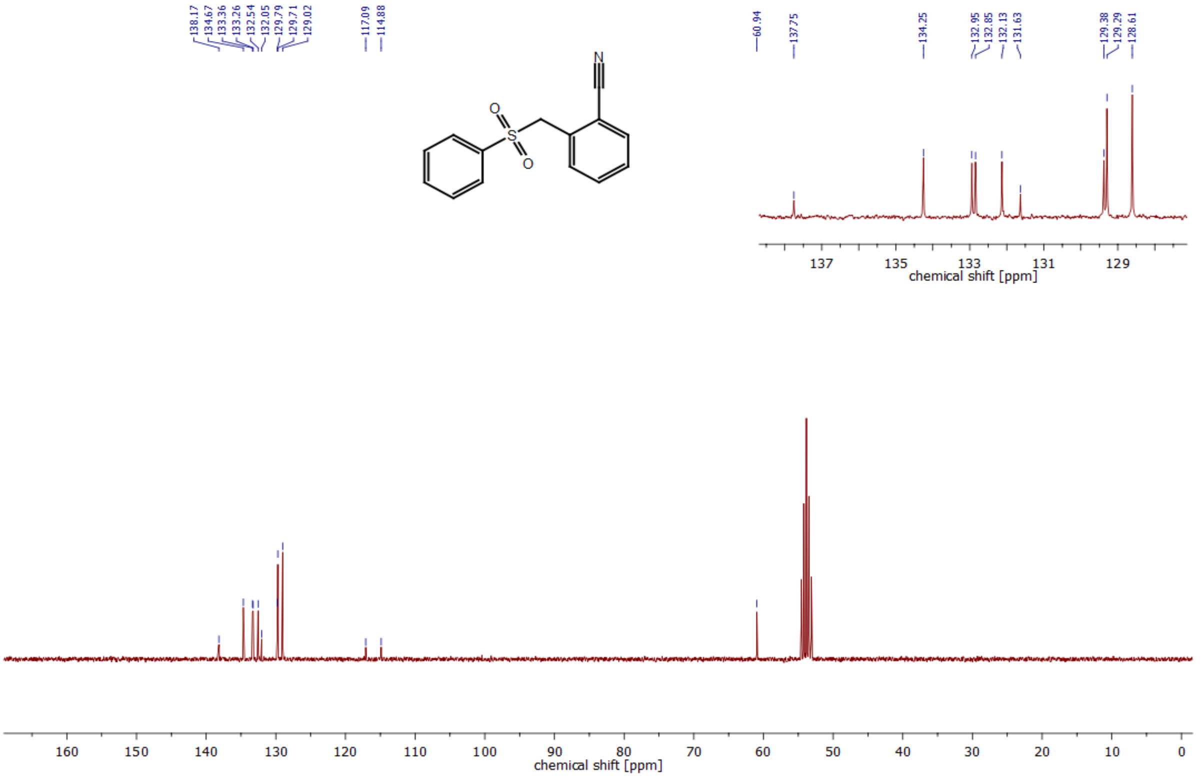

Figure S4. ^{13}C-NMR spectra of 2-((phenylsulfonyl)methyl)benzonitrile (**S1**, 75 MHz, $CDCl_3$).

2.2 2-((phenylsulfonyl)methyl)benzaldehyde (S2)

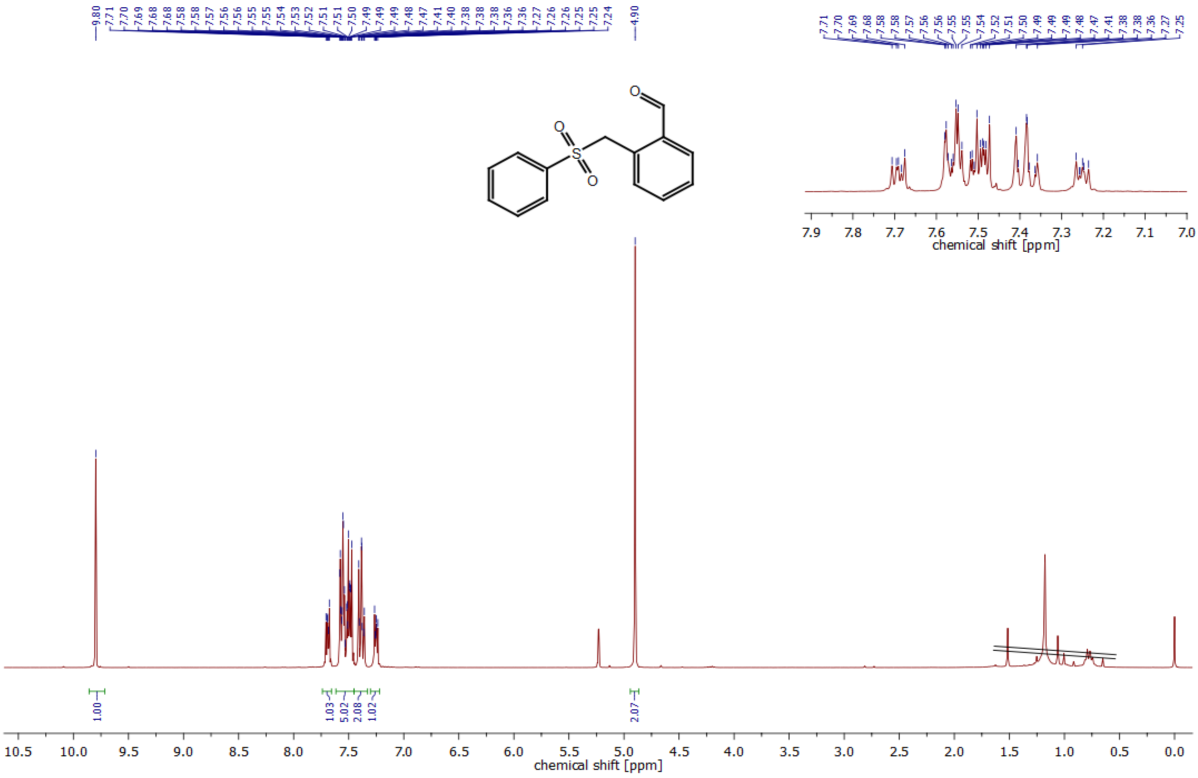

Figure S5. ^{1}H-NMR spectra of 2-((phenylsulfonyl)methyl)benzaldehyde (**S2**, 300 MHz, $CDCl_3$). 1.55 ppm: H_2O, 1.27/0.8 ppm: H-grease.[3]

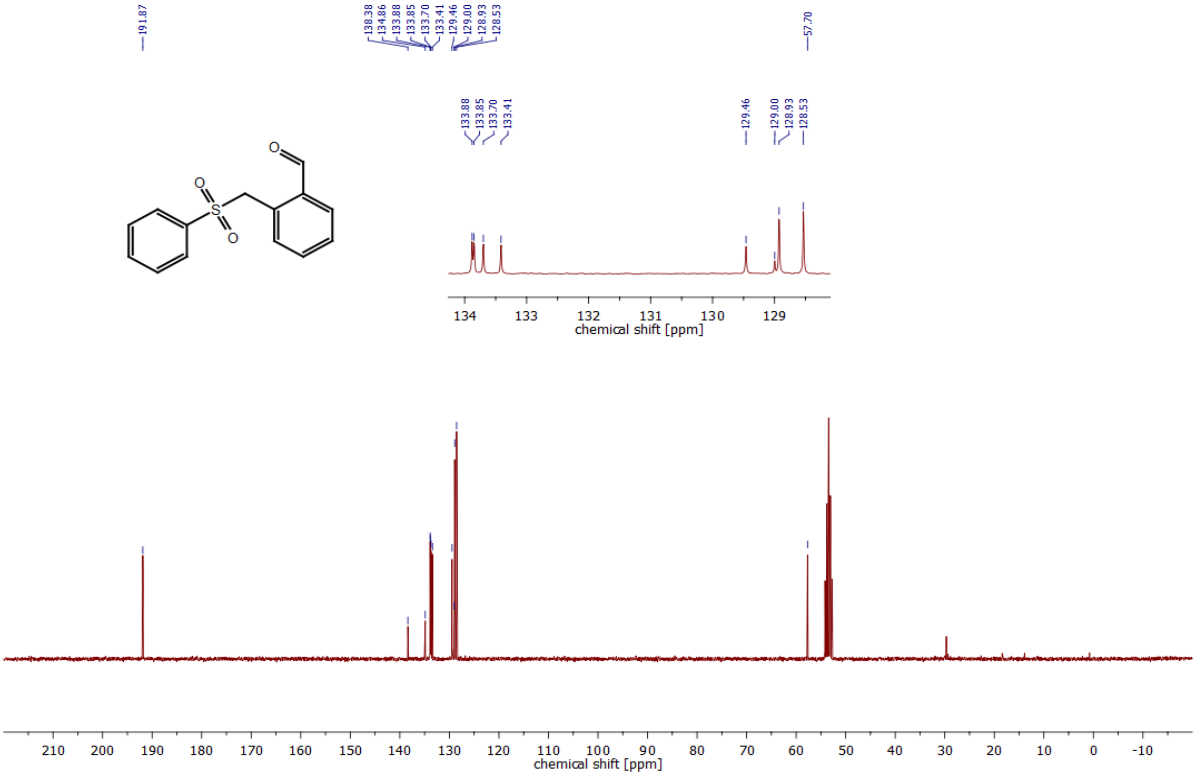

Figure S6. ^{13}C-NMR spectra of 2-((phenylsulfonyl)methyl)benzaldehyde (**S2**, 75 MHz, $CDCl_3$).

2.3 Acetylene building block 6

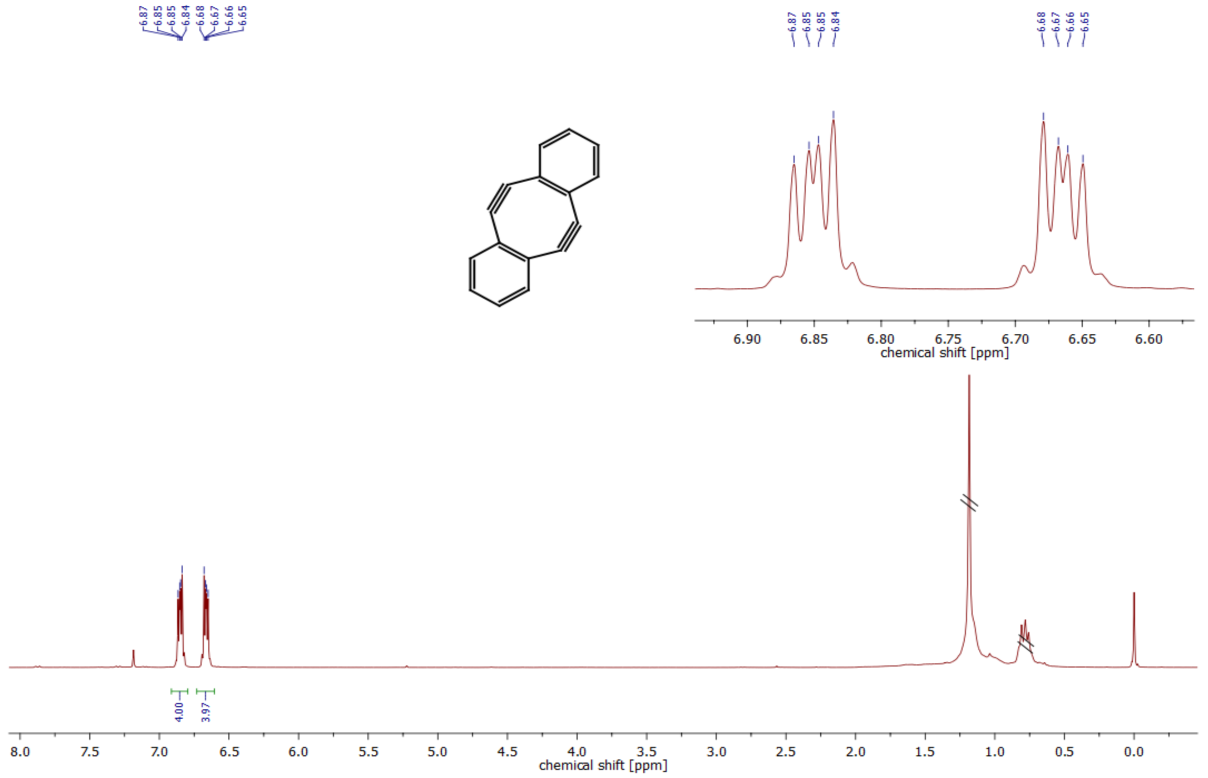

Figure S7. ^{1}H-NMR spectra of diacetylene building block **6** (300 MHz, $CDCl_3$). 1.27/0.8 ppm: H-grease.[3]

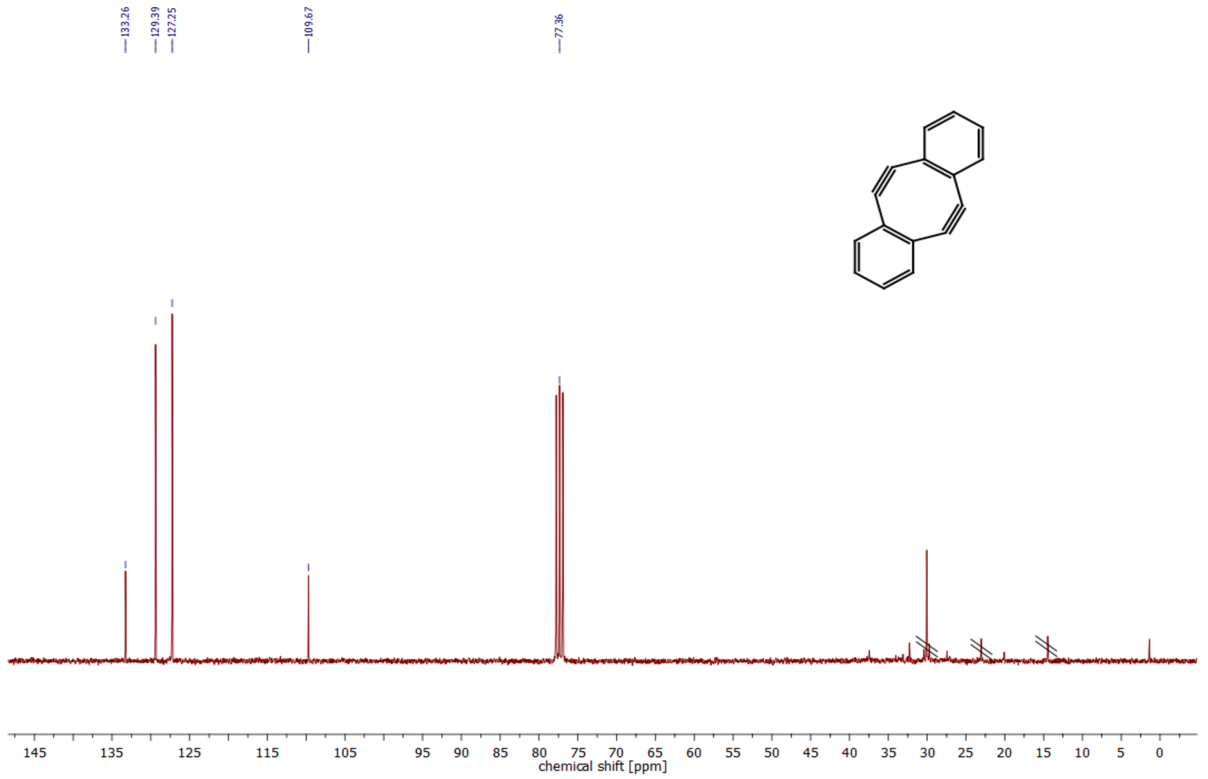

Figure S8. ^{13}C-NMR spectra of diacetylene building block **6** (75 MHz, $CDCl_3$). Aliphatic impurities: H-grease.[3]

2.4 2,7-di-*tert*-butylpyrene (2)

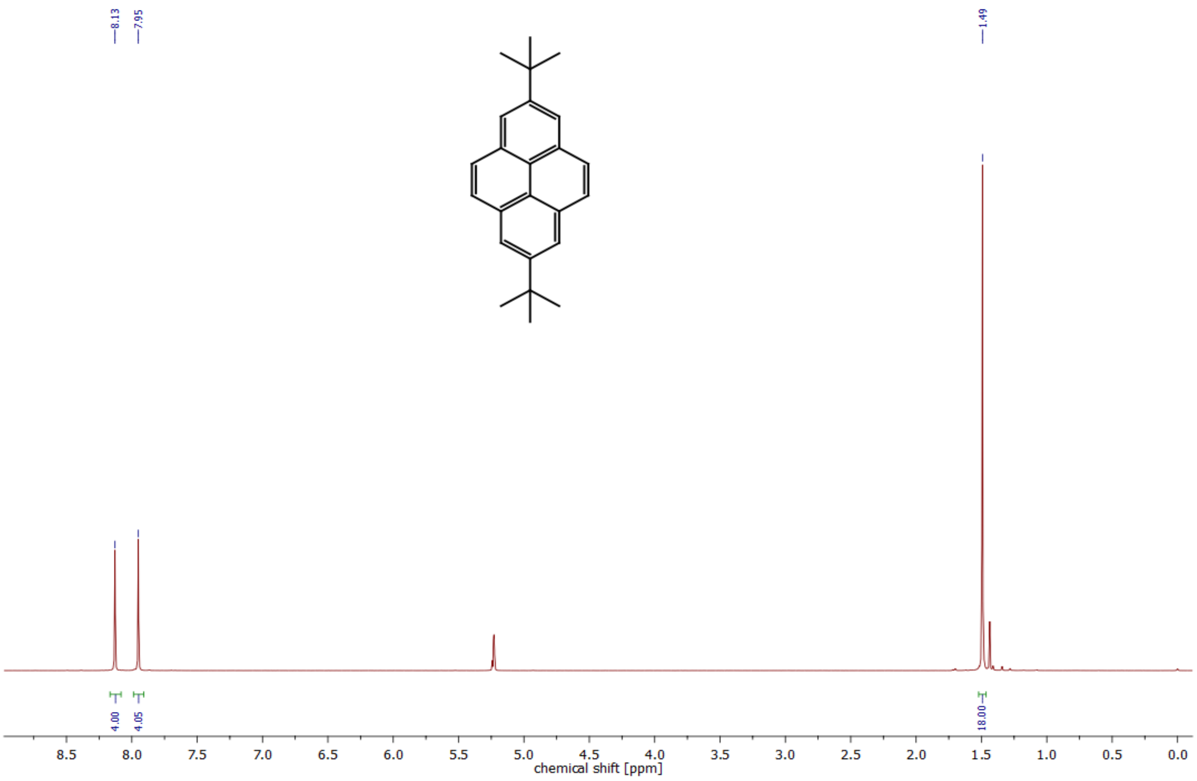

Figure S9. ^{1}H-NMR spectra of 2,7-di*tert*-butylpyrene (**2**, 300 MHz, CD_2Cl_2).

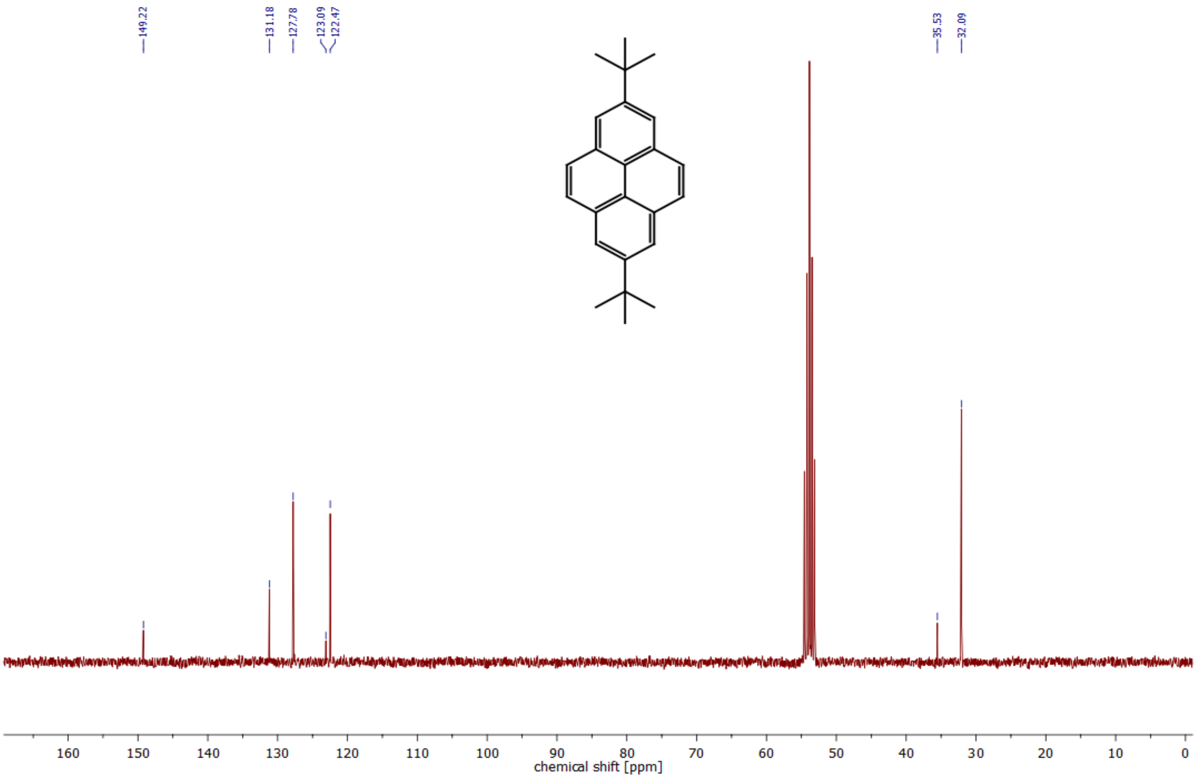

Figure S 10. ^{13}C-NMR spectra of 2,7-di*tert*-butylpyrene (**2,** 75 MHz, CD_2Cl_2).

2.5 2,7-di-*tert*-butylpyrene-4,5-dione (3)

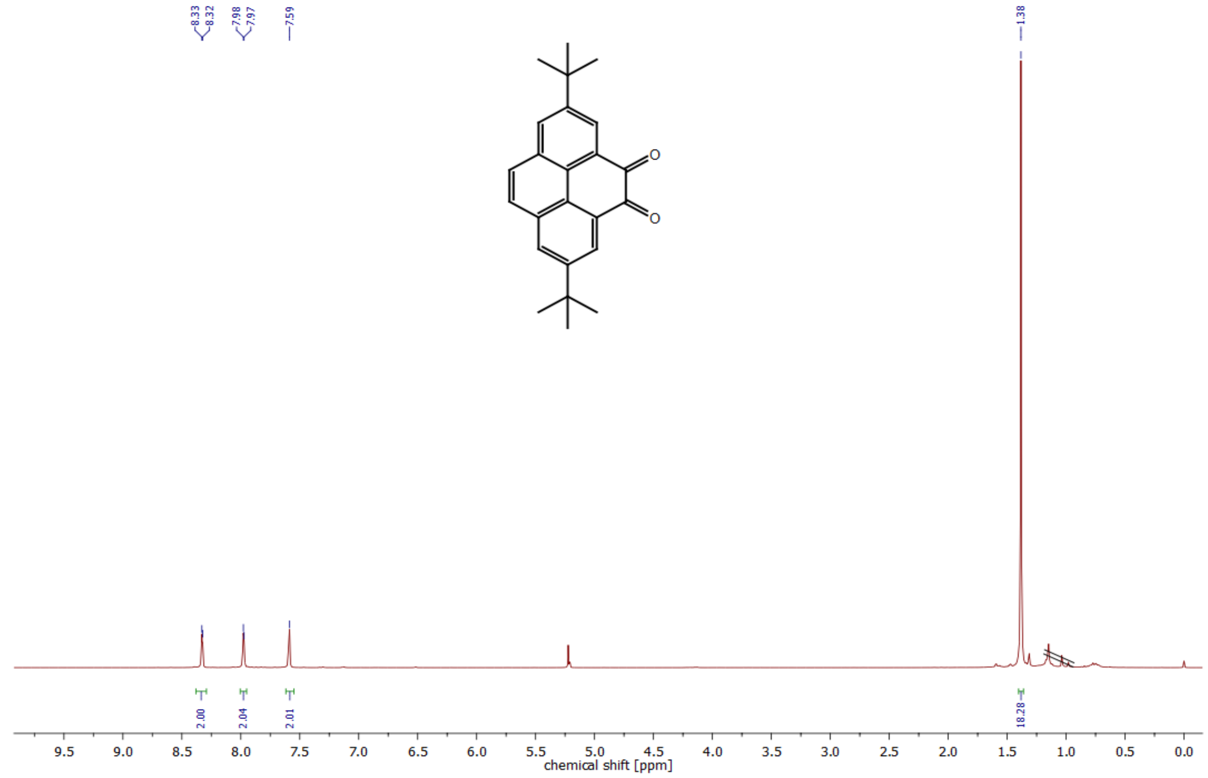

Figure S11. ^{1}H-NMR spectra of compound **3** (300 MHz, CD_2Cl_2).

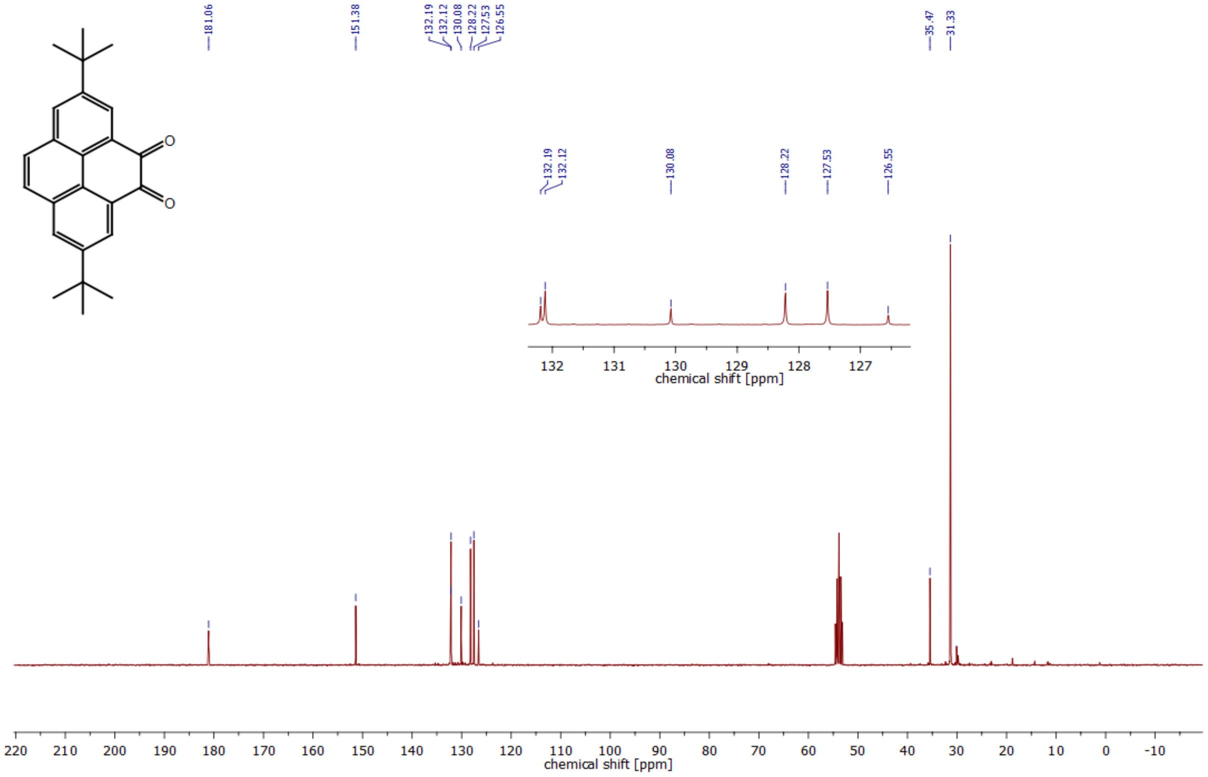

Figure S12. ^{13}C-NMR spectra of 2,7-di*tert*-butylpyrene (**3,** 75 MHz, CD_2Cl_2).

2.6 1,3-bis(4-(*tert*-butyl)phenyl)propan-2-one (4)

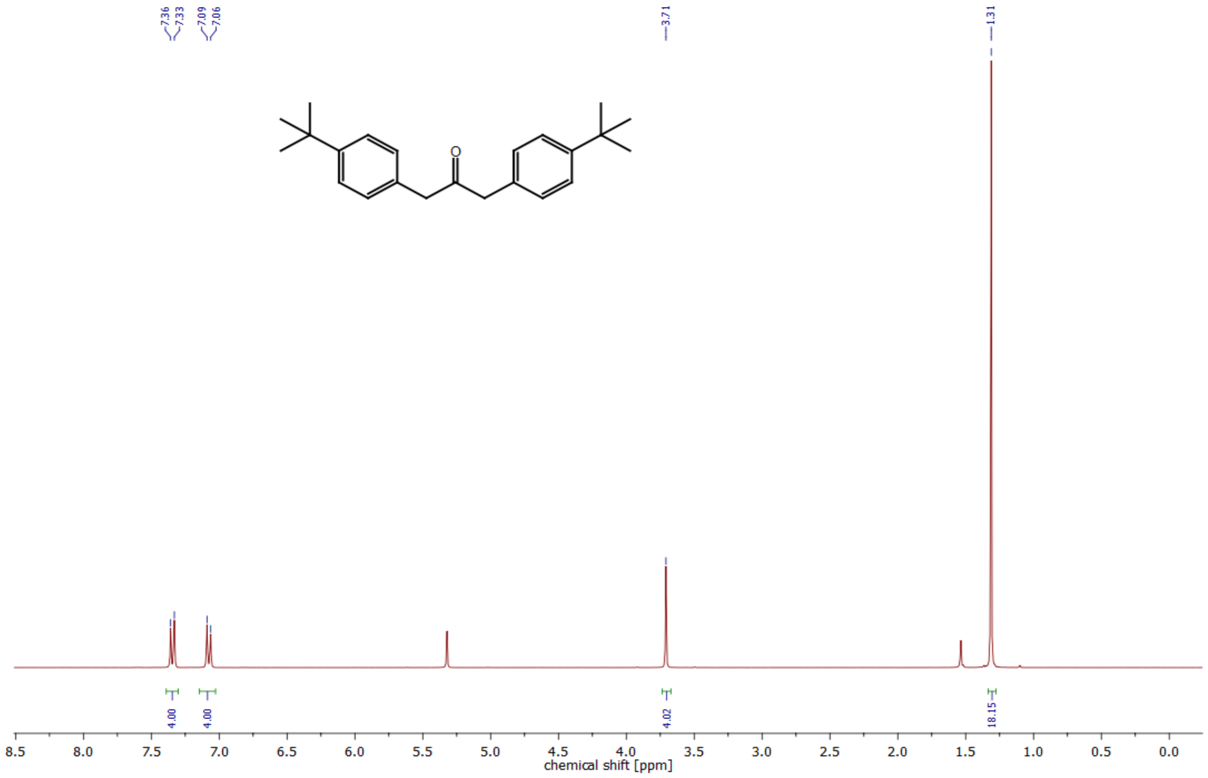

Figure S13. ^{1}H-NMR spectra of compound **4** (300 MHz, CD_2Cl_2). 1.55 ppm: H_2O.

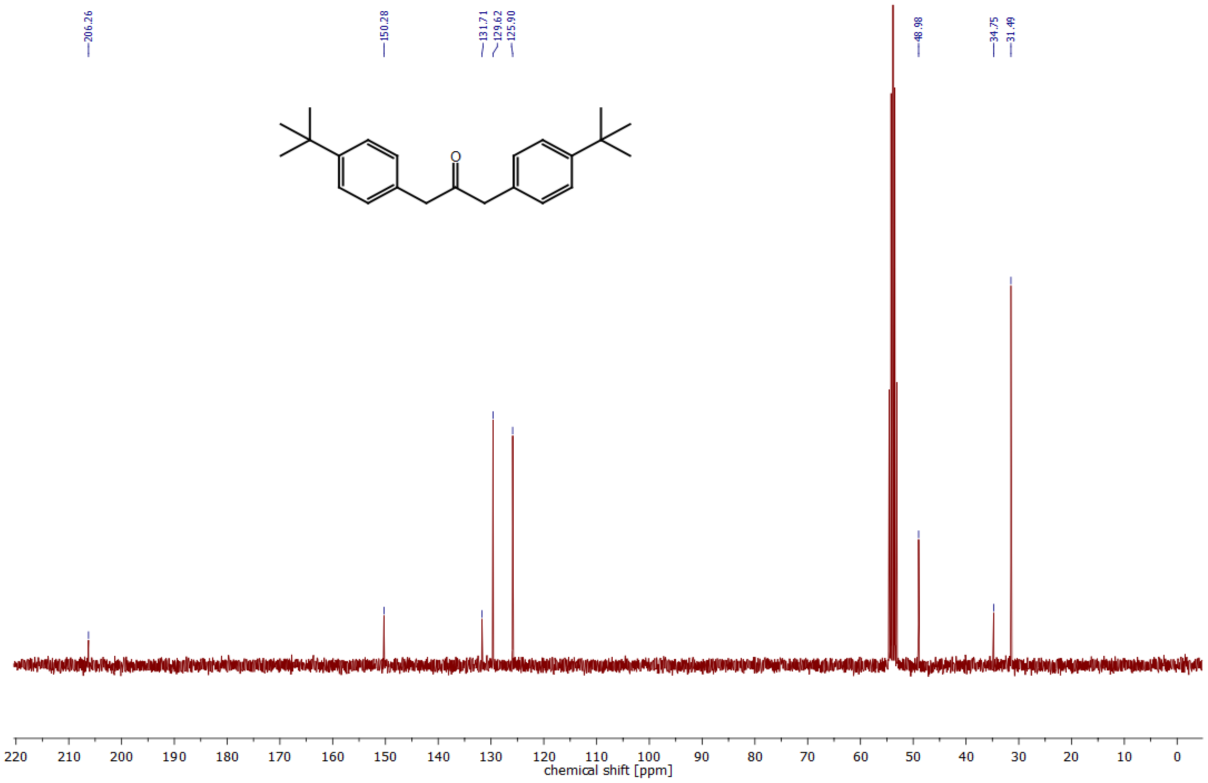

Figure S14. ^{13}C-NMR spectra of compound **4** (75 MHz, CD_2Cl_2).

2.7 Cyclopentadienone 5

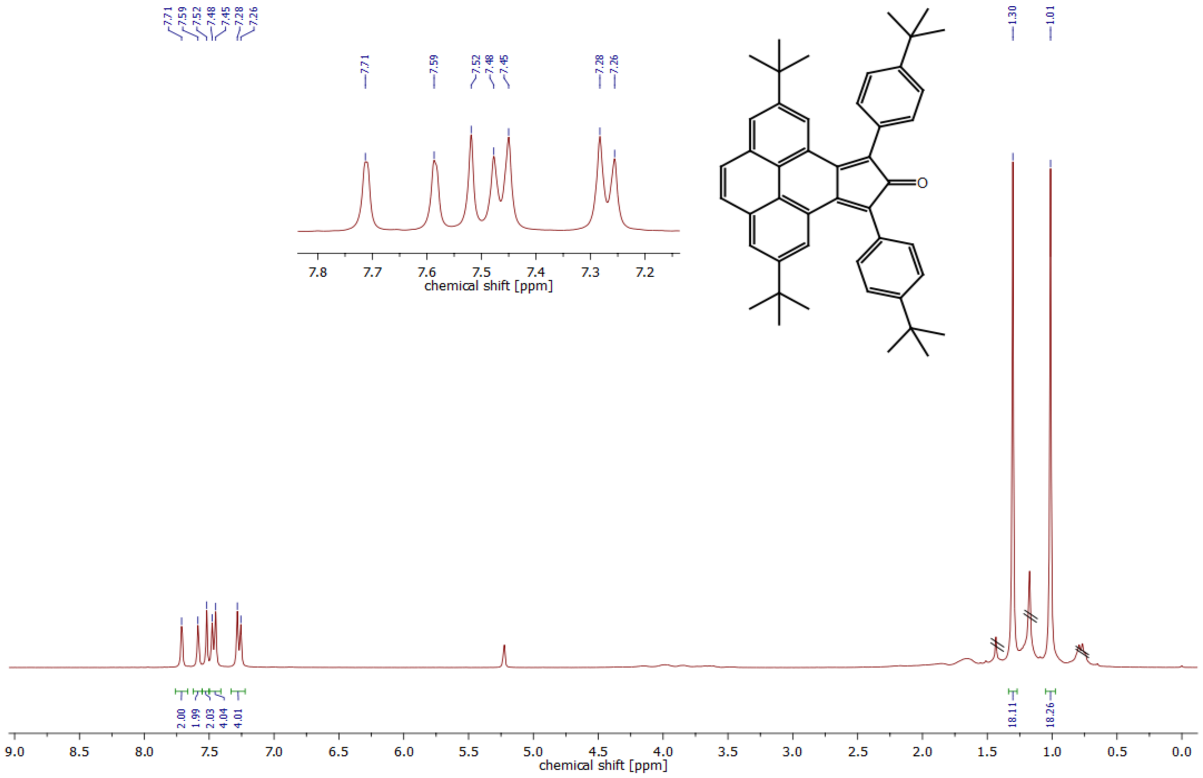

Figure S15. ^{1}H-NMR spectra of compound **5** (300 MHz, CD_2Cl_2). 1.27/0.8 ppm: H-grease.[3]

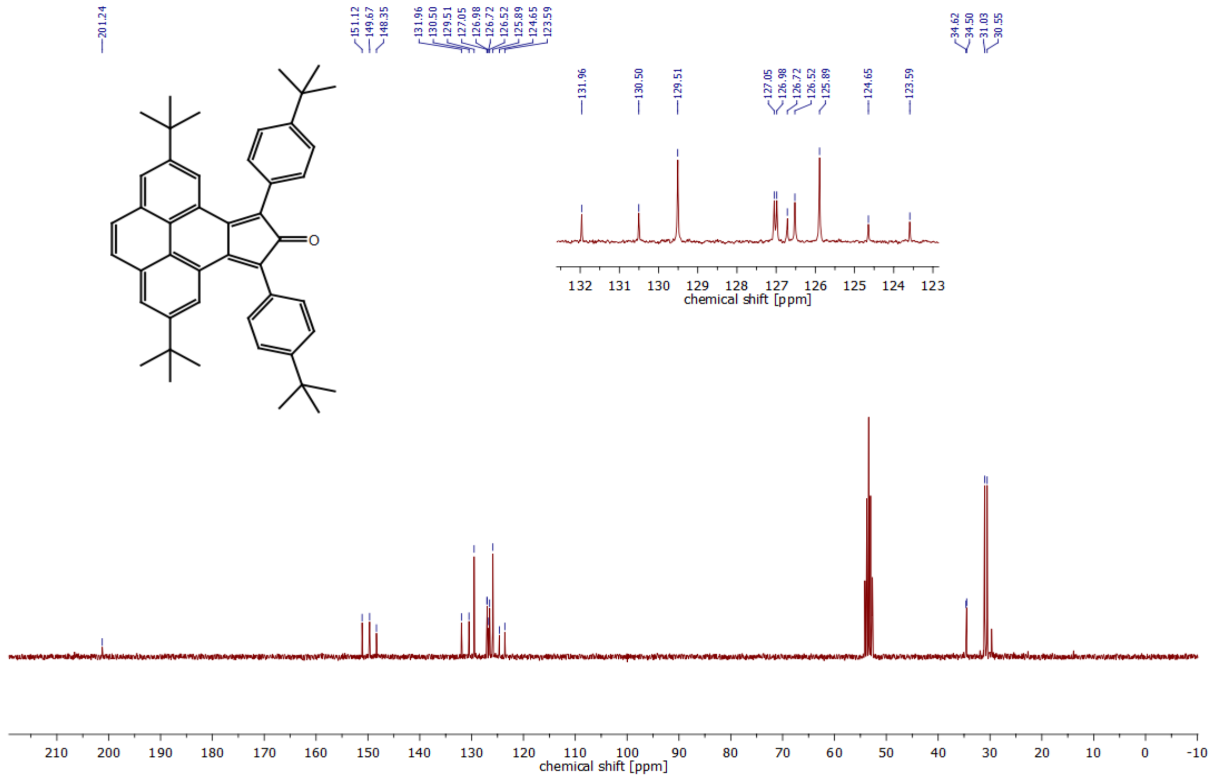

Figure S16. ^{13}C-NMR spectra of compound **5** (75 MHz, CD_2Cl_2).

2.8 Model compound precursor 7

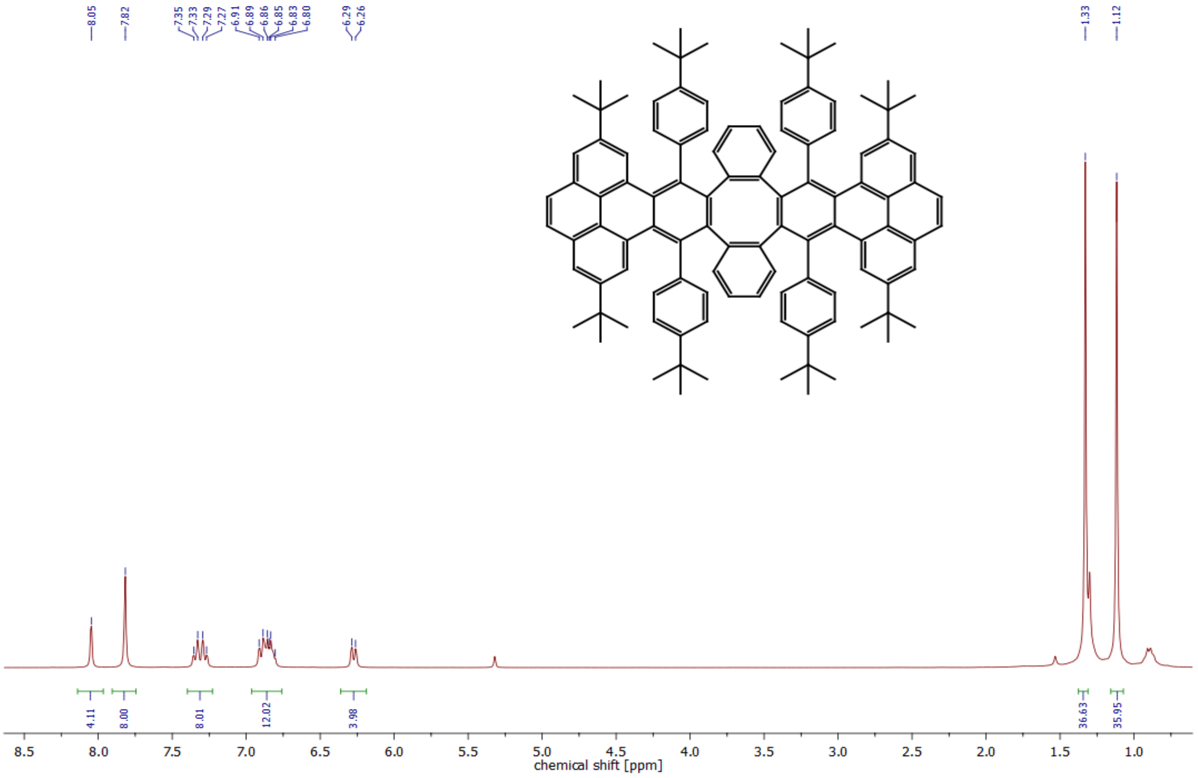

Figure S17. ^{1}H-NMR spectra of model compound precursor **7** (300 MHz, CD_2Cl_2).

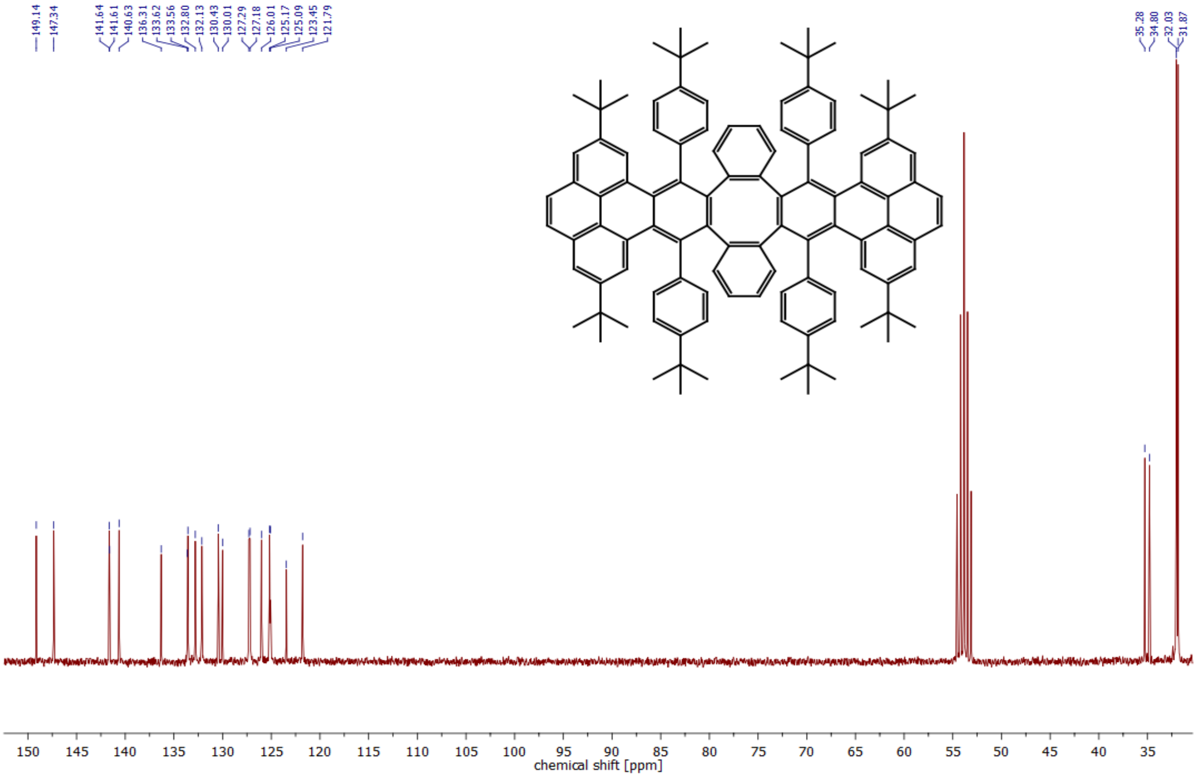

Figure S18. ^{13}C-NMR spectra of model compound precursor **7** (75 MHz, CD_2Cl_2).

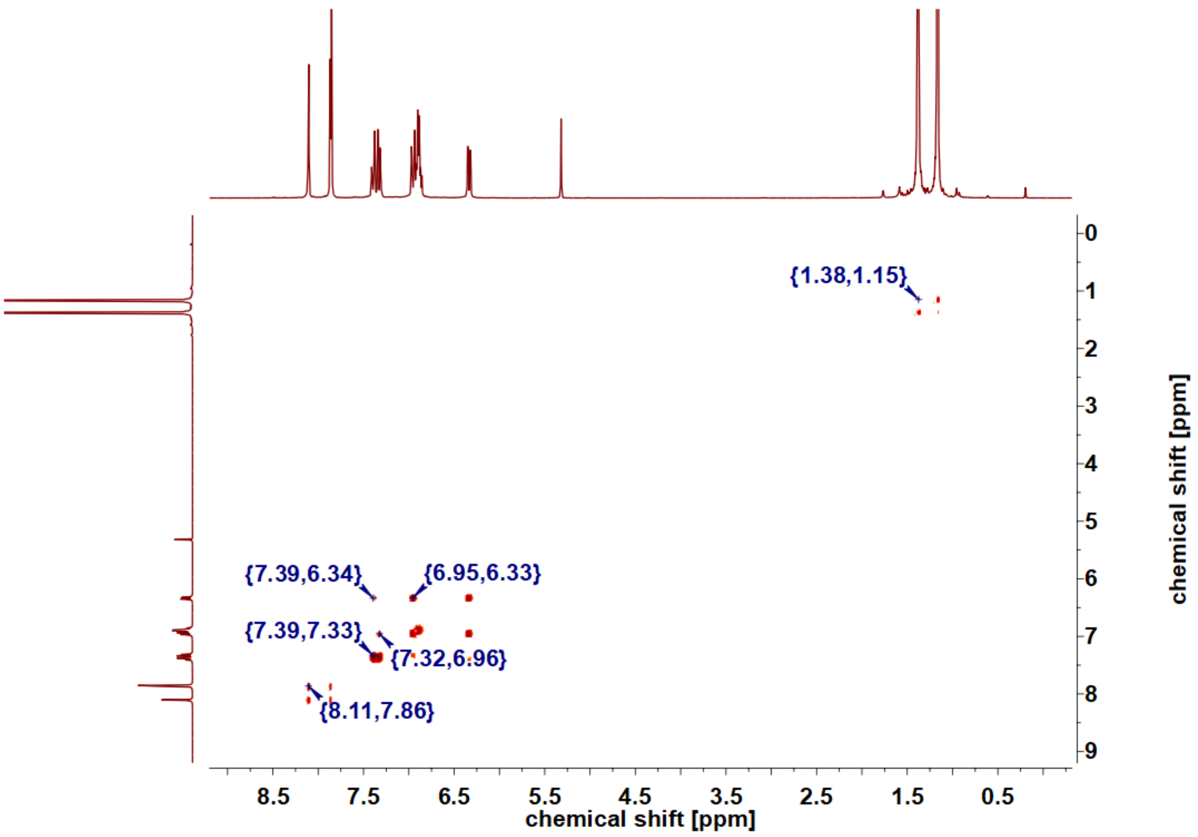

Figure S19. COSY-Experiment of compound **7** in CD_2Cl_2 (300 MHz).

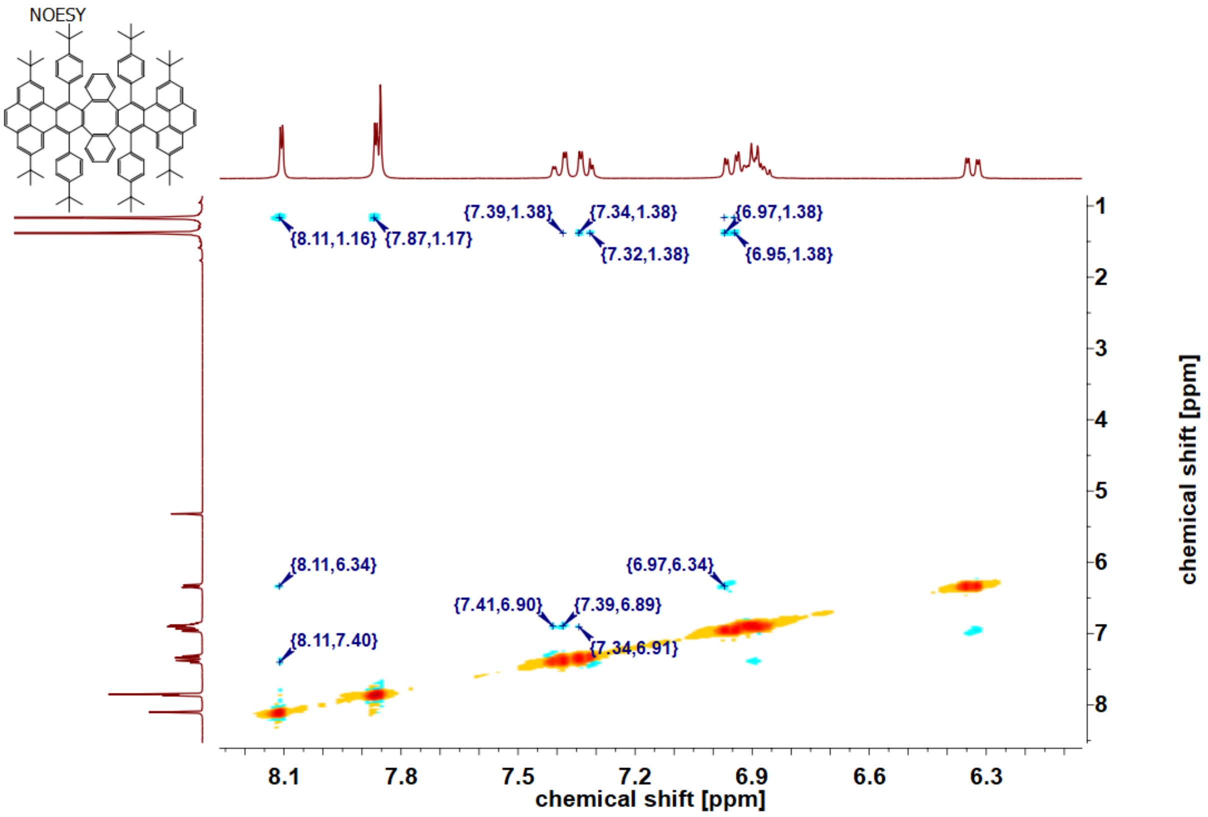

Figure S20. NOESY-Experiment of compound **7** in CD_2Cl_2 (300 MHz).

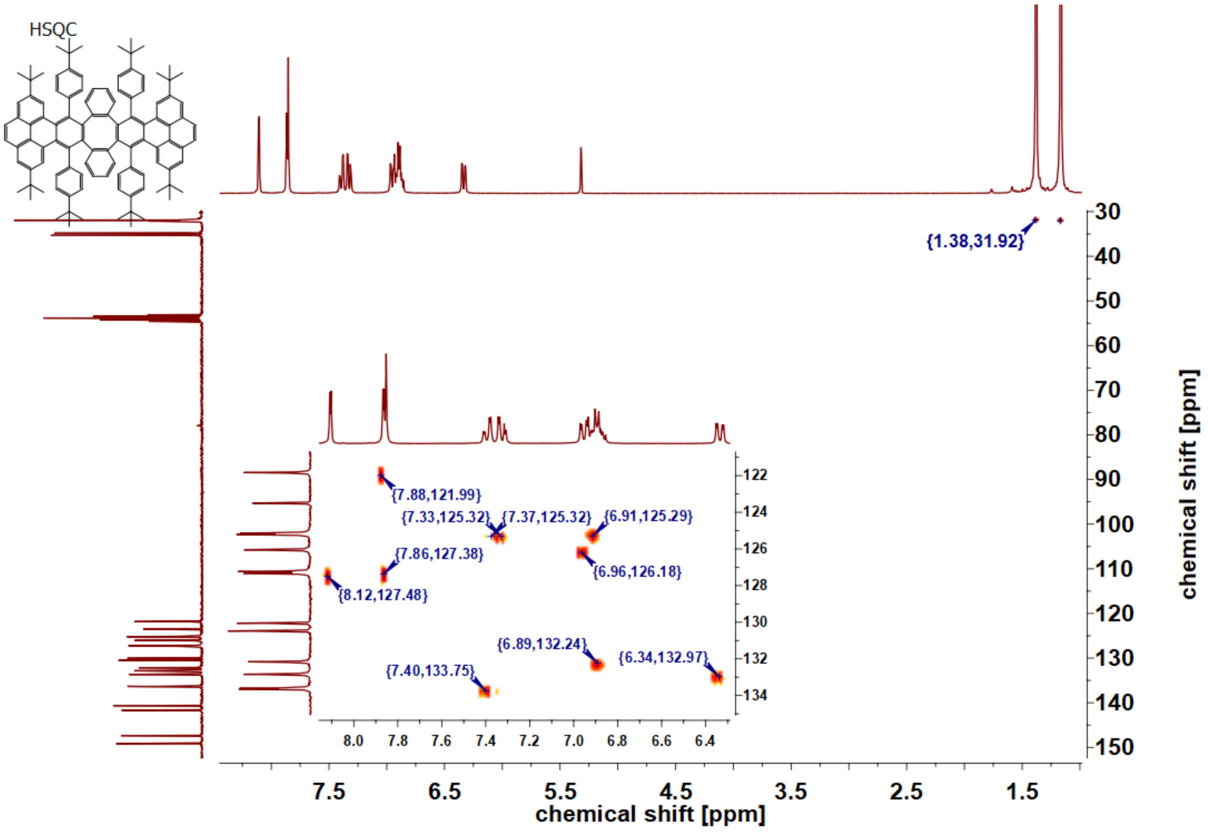

Figure S21. HSQC-Experiment of compound **7** in CD_2Cl_2 (300/75 MHz).

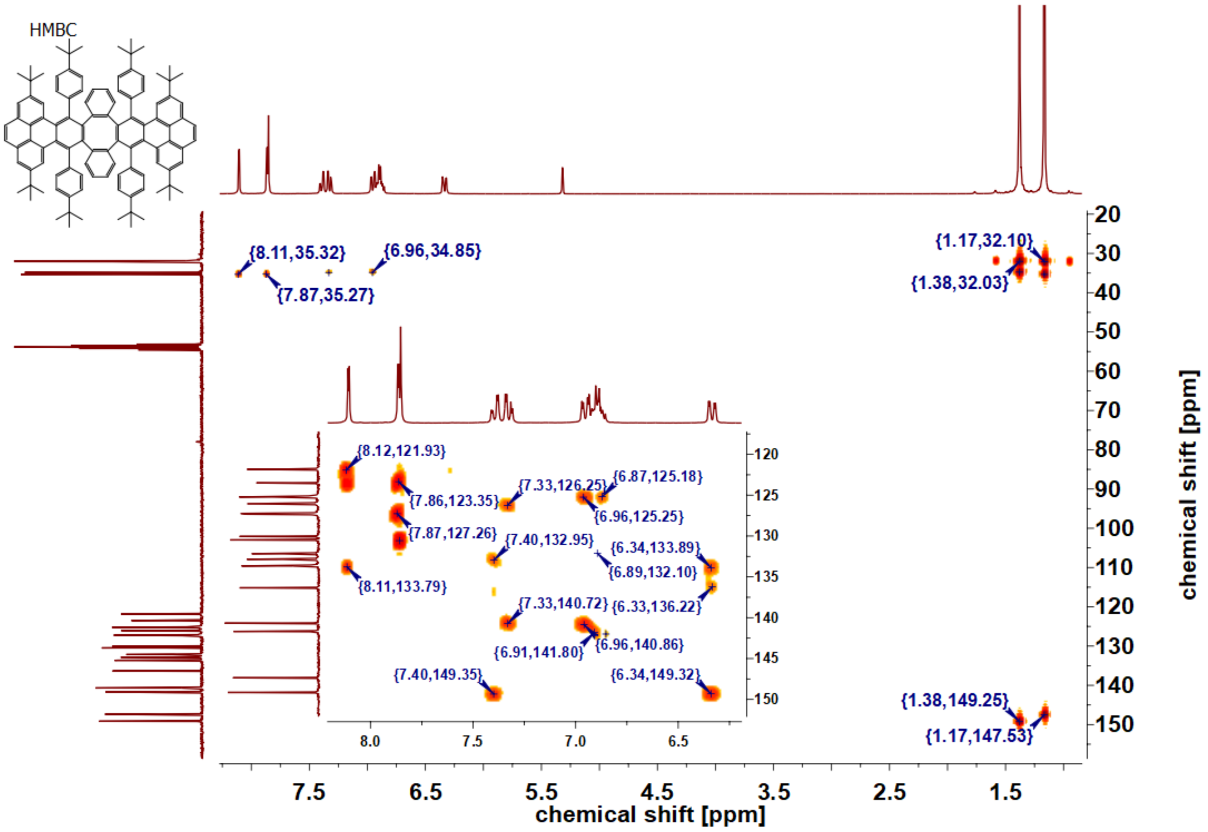

Figure S22. HMBC-Experiment of compound **7** in CD_2Cl_2 (300/75 MHz).

2.9 Model compound 1

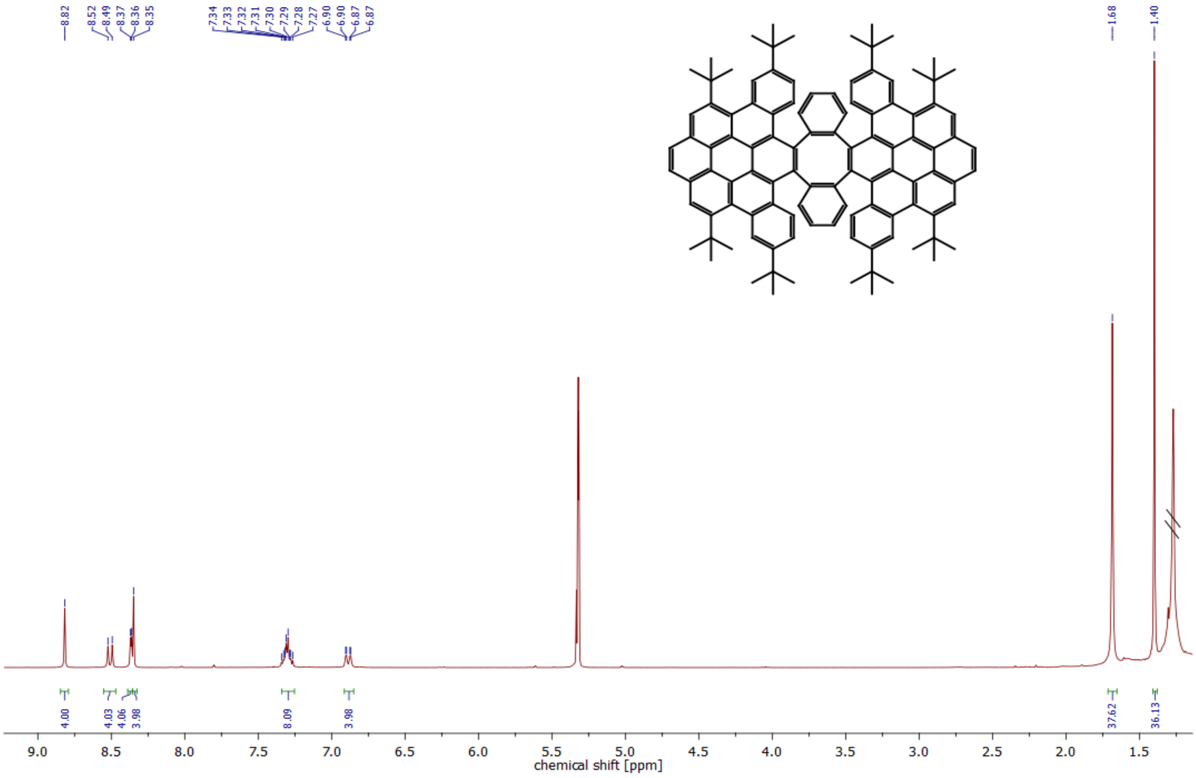

Figure S23. ^{1}H-NMR spectra of model compound **1** (300 MHz, CD_2Cl_2). 1.27/0.8 ppm: H-grease.[3]

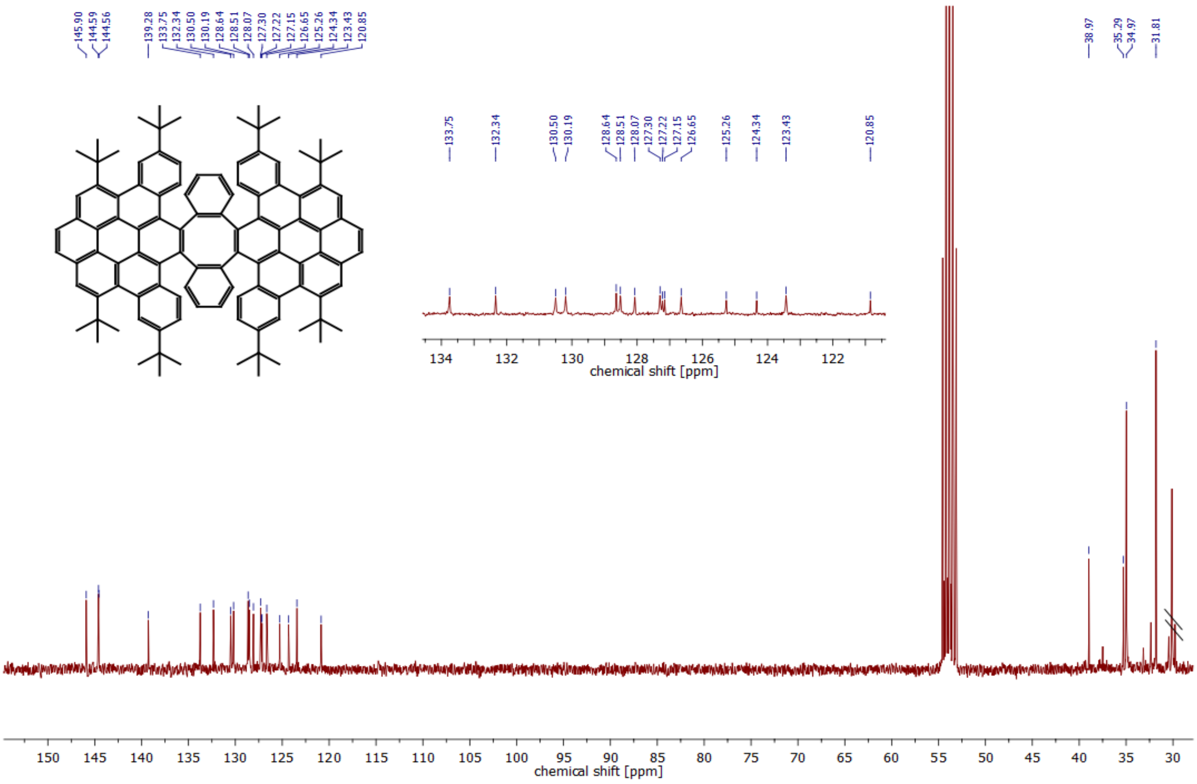

Figure S24. ^{13}C-NMR spectra of model compound **1** (75 MHz, CD_2Cl_2). Aliphatic impurities: H-grease.[3]

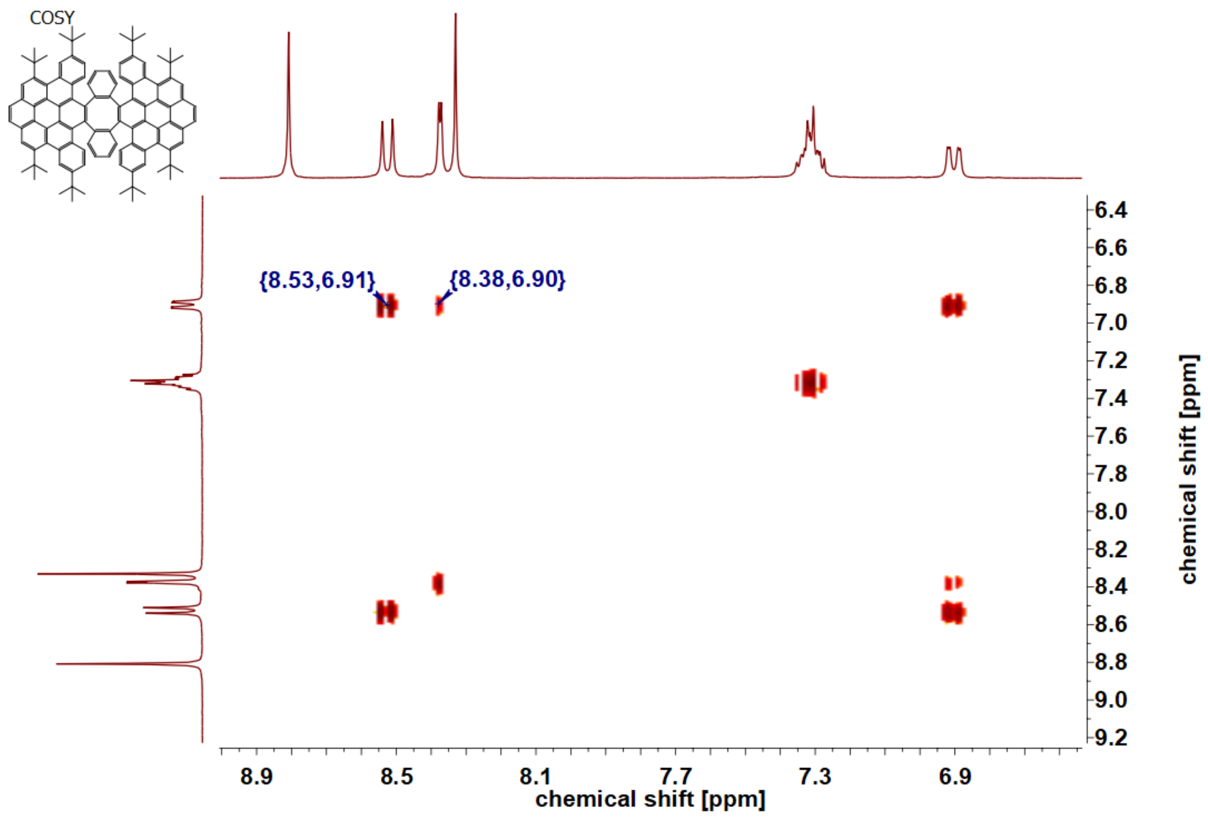

Figure S25. COSY-Experiment of compound **1** in CD_2Cl_2 (300 MHz).

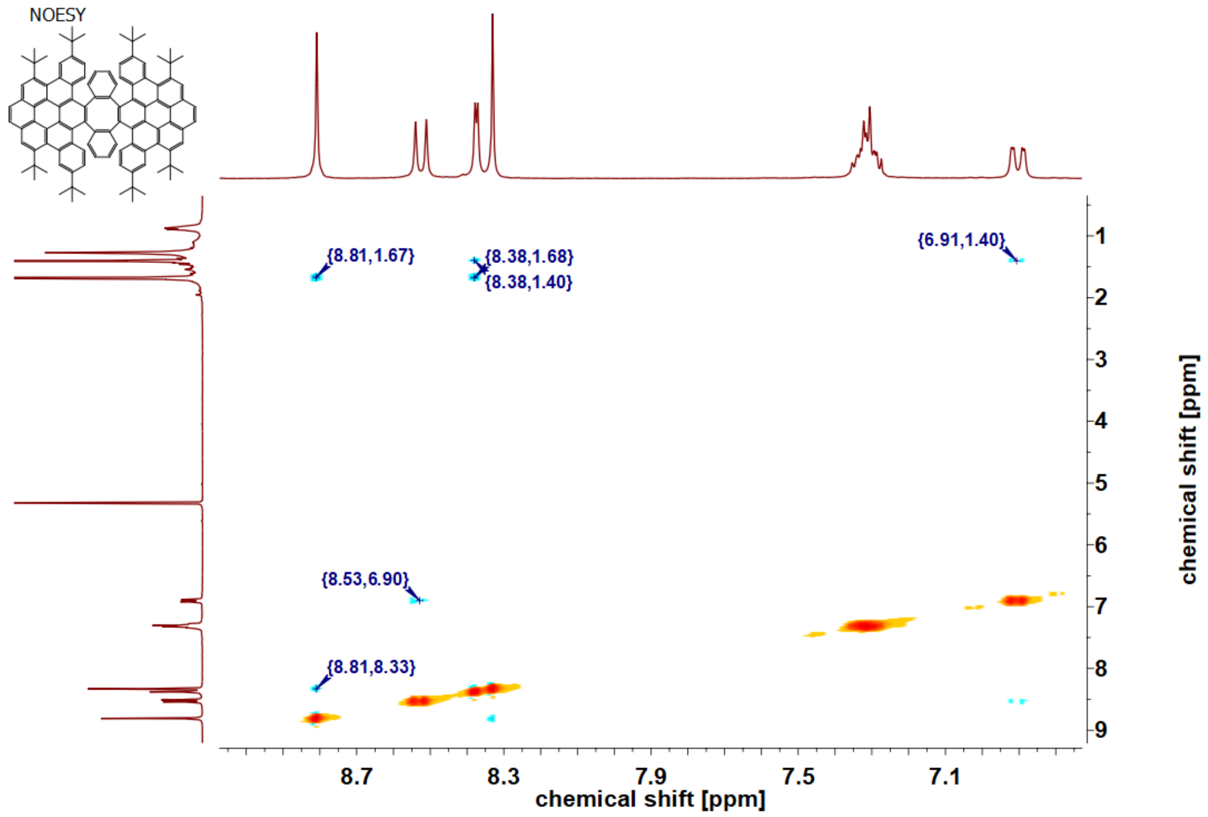

Figure S26. NOESY-Experiment of compound **1** in CD_2Cl_2 (300 MHz).

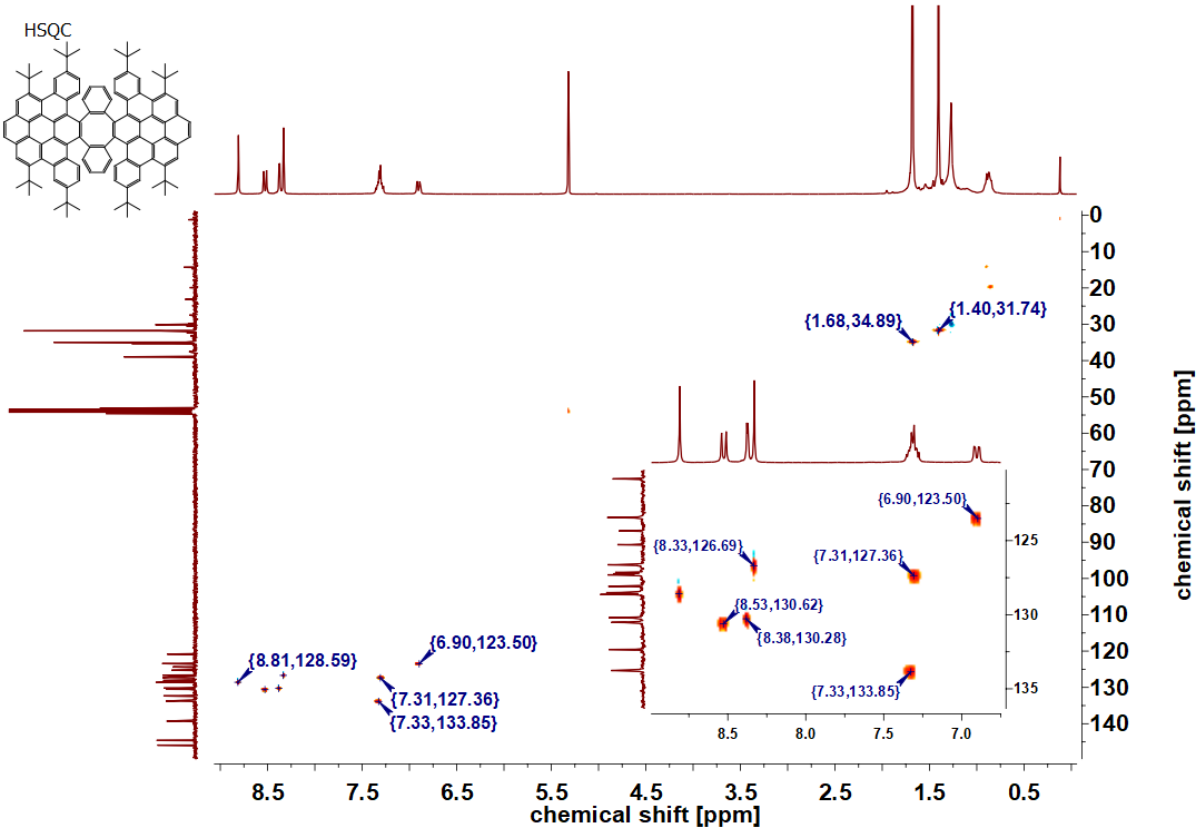

Figure S27. HSQC-Experiment of compound **1** in CD_2Cl_2 (300/75 MHz).

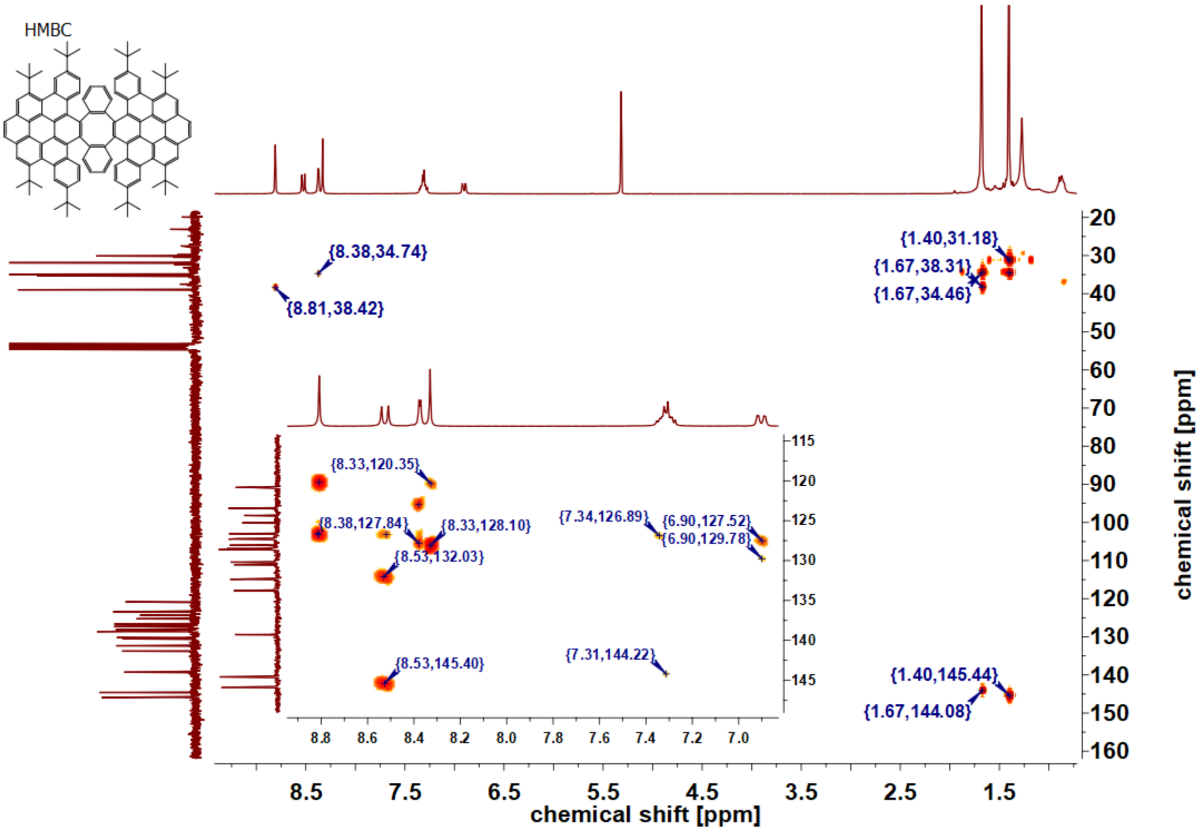

Figure S28. HMBC-Experiment of compound **1** in CD_2Cl_2 (300/75 MHz).

2.10 1,3-bis(4-bromophenyl)propan-2-one S4

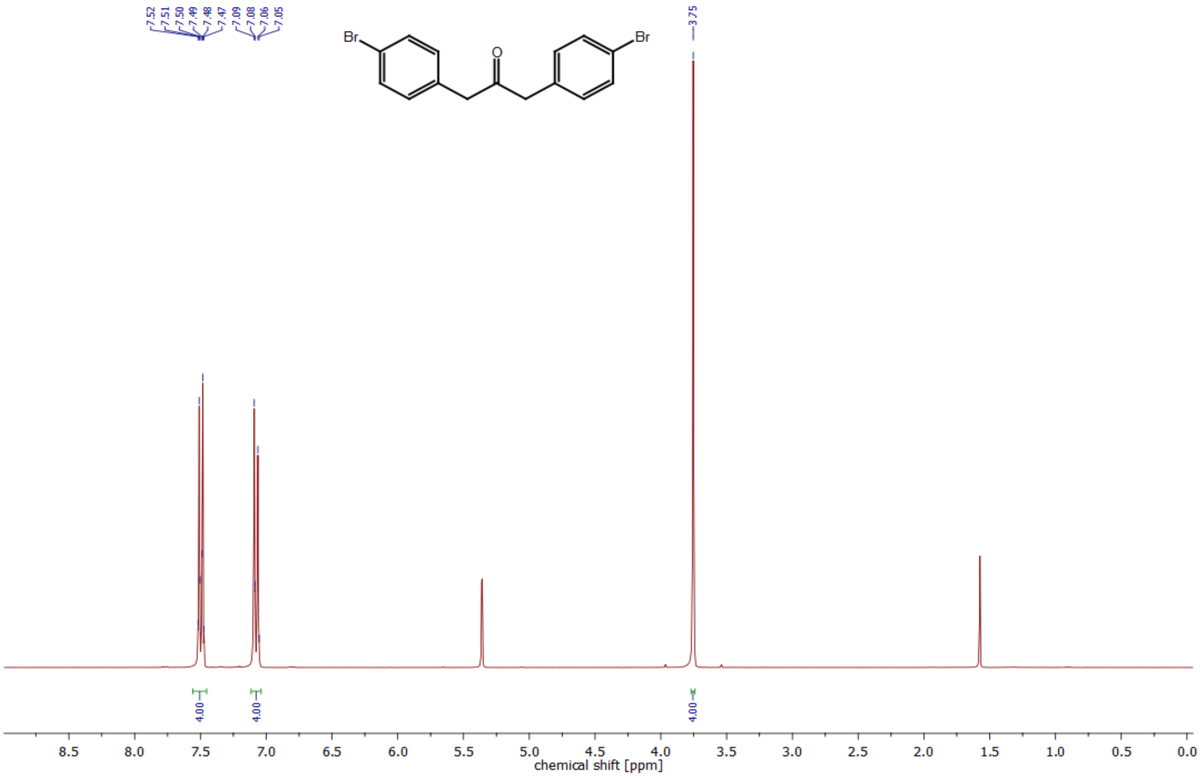

Figure S29. ^{1}H-NMR spectra of model compound **S4** (300 MHz, CD_2Cl_2). 1.55 ppm: H_2O.

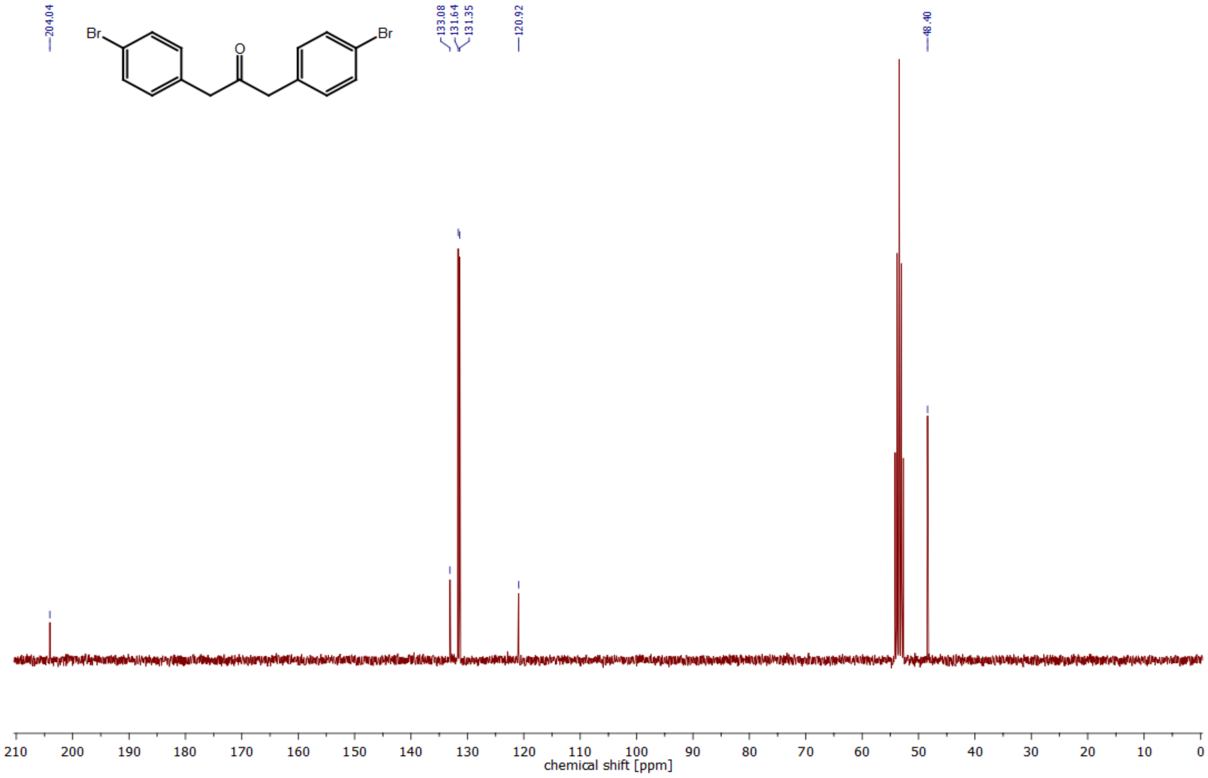

Figure S30. ^{13}C-NMR spectra of model compound **S4** (75 MHz, CD_2Cl_2).

2.11 1,3-bis(4-dodecylphenyl)propan-2-one S5

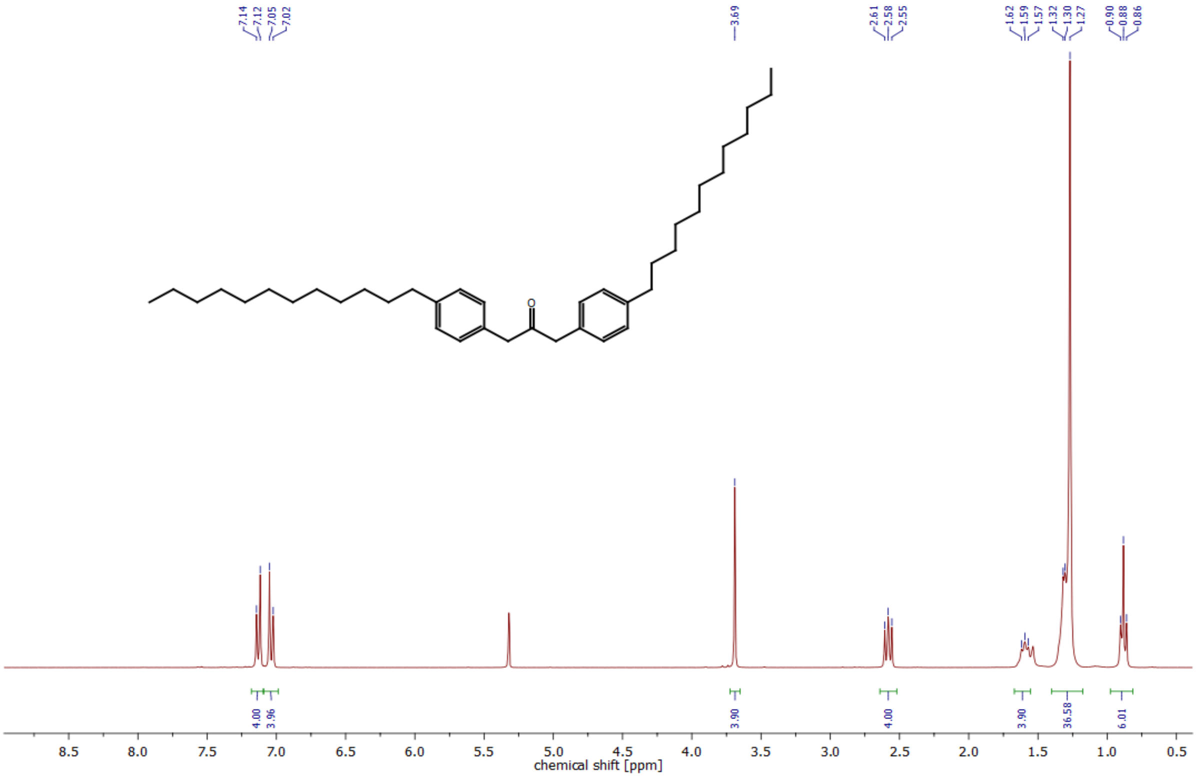

Figure S31. ^{1}H-NMR spectra of model compound **S5** (300 MHz, CD_2Cl_2).

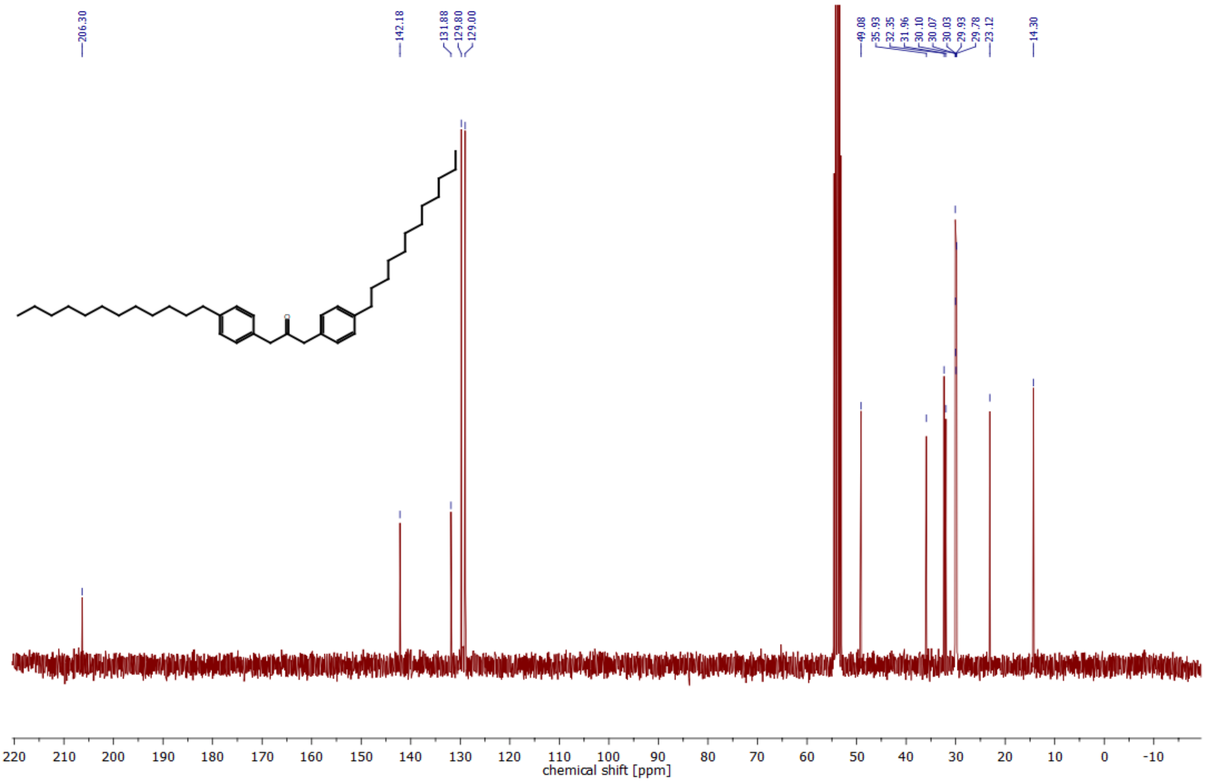

Figure S32. ^{13}C-NMR spectra of model compound **S5** (75 MHz, CD_2Cl_2).

2.12 2,7-di-*tert*-butylpyrene-4,5,9,10-tetraone (S6)

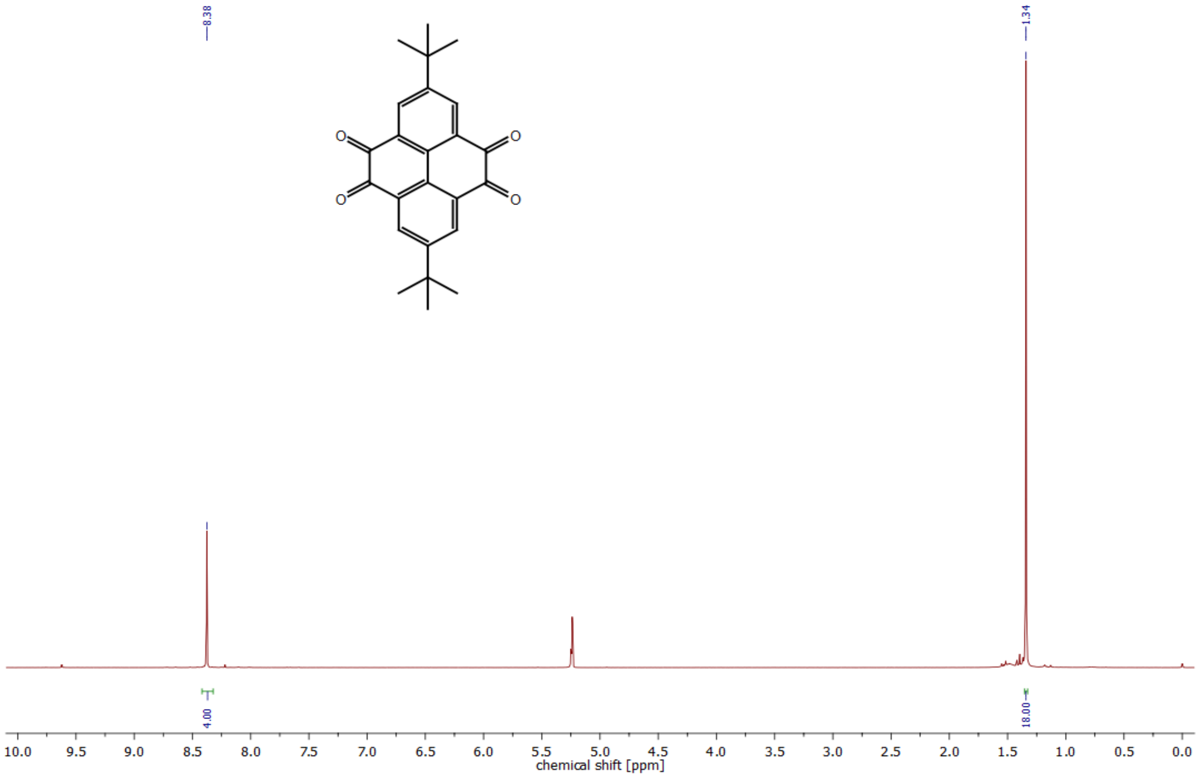

Figure S33. ^{1}H-NMR spectra of model compound **S6** (300 MHz, CD_2Cl_2).

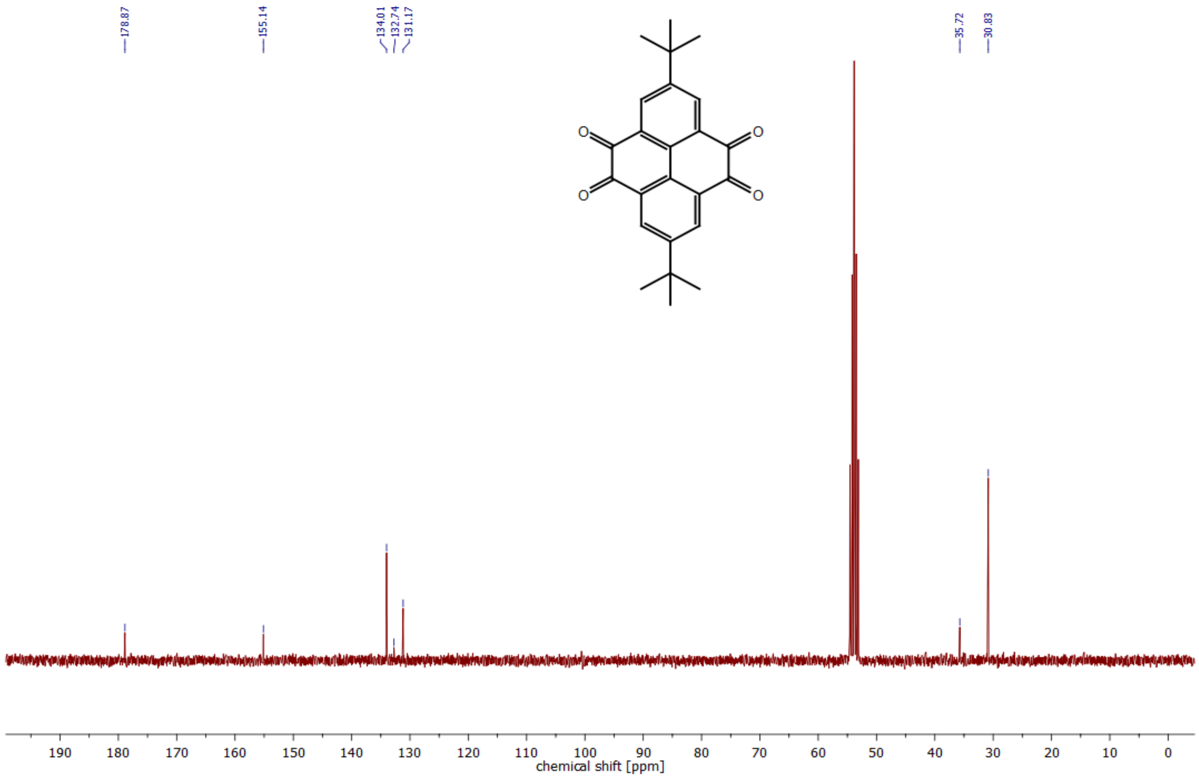

Figure S34. ^{13}C-NMR spectra of model compound **S6** (75 MHz, CD_2Cl_2).

2.13 Cyclopentadienone 8

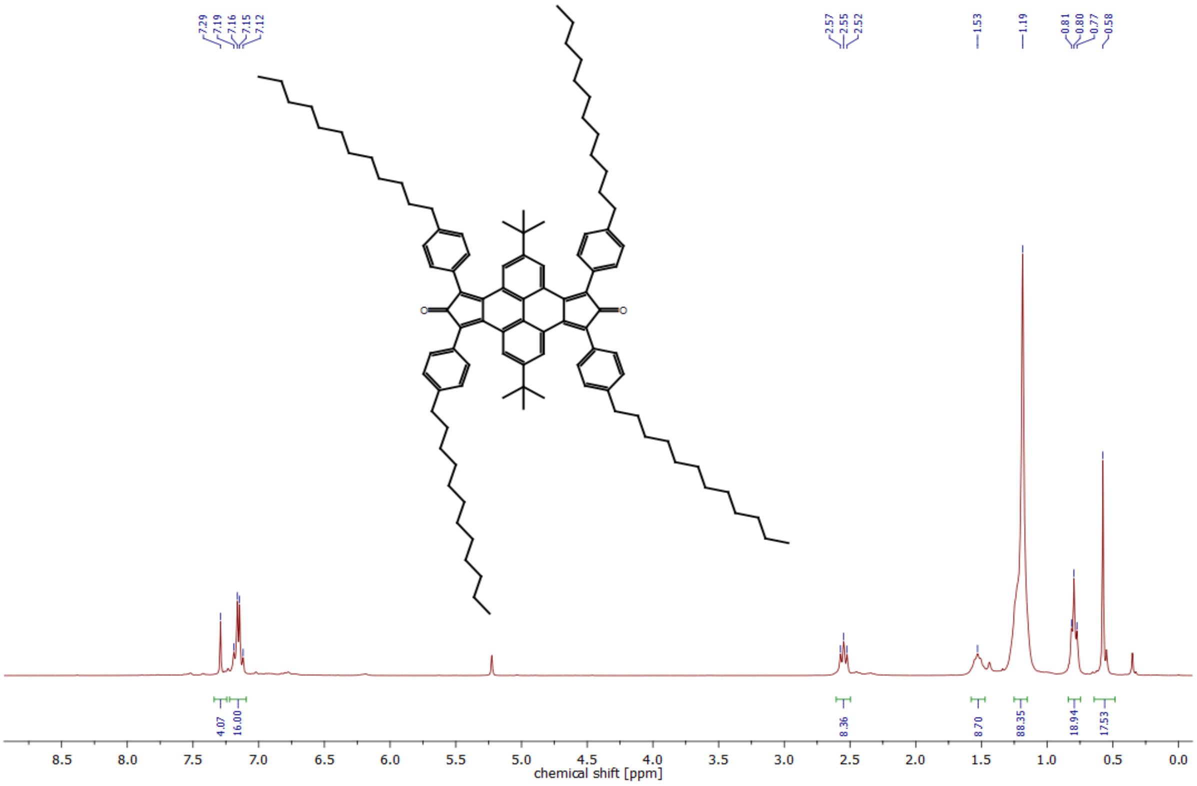

Figure S35. ^{1}H-NMR spectra of model compound **8** (300 MHz, CD_2Cl_2).

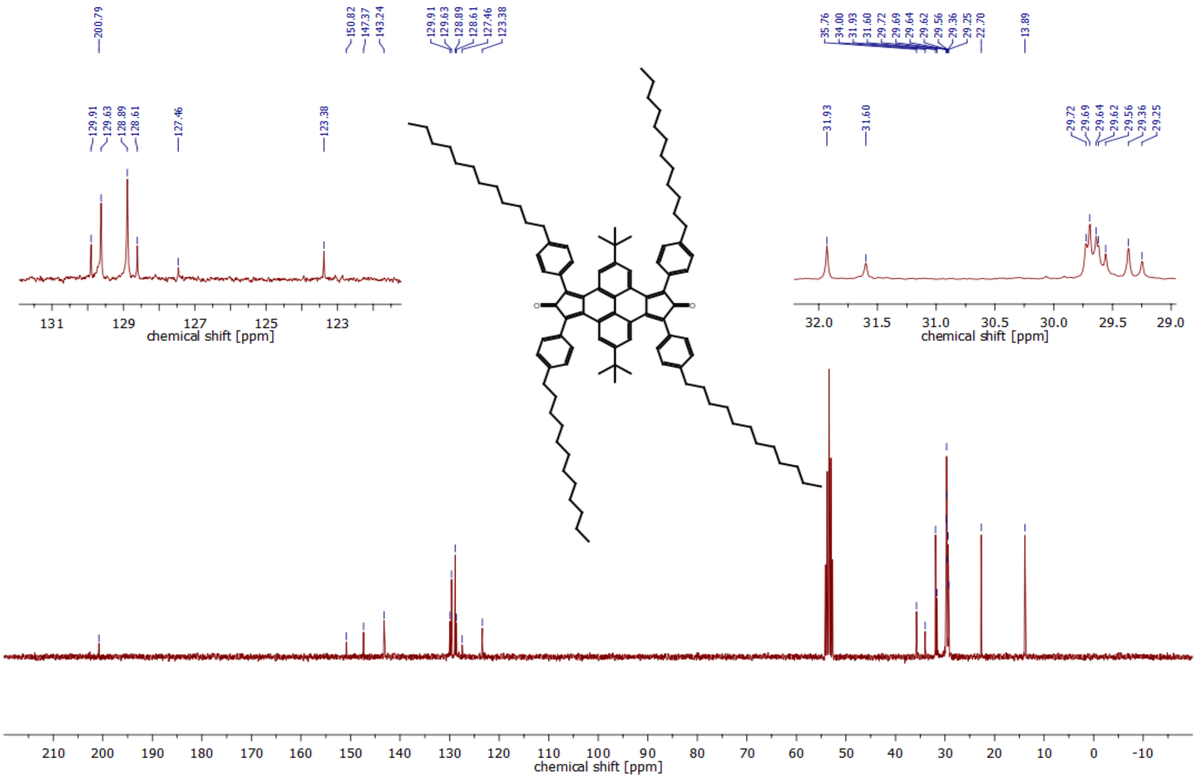

Figure S36. ^{13}C-NMR spectra of model compound **8** (75 MHz, CD_2Cl_2).

2.14 LTP and wGNR

Figure S37 shows an NMR stack of building block **8**, **LTP** and **wGNR**. **LTP** shows only broad signals for the aromatic backbone. However, the pattern of the alkyl chains matches building block **8**. In case of **wGNR**, a further broadening and reduction in aromatic ^{1}H-integrals can be observed, almost showing no aromatic signals. In addition, the CH_2-groups attached to the aromatic backbone (~2.6 ppm) are missing as well, which still speaks for some degree of π-π-aggregation in solution. The broadening in case of **wGNR** is too severe to assign integrals reliably.

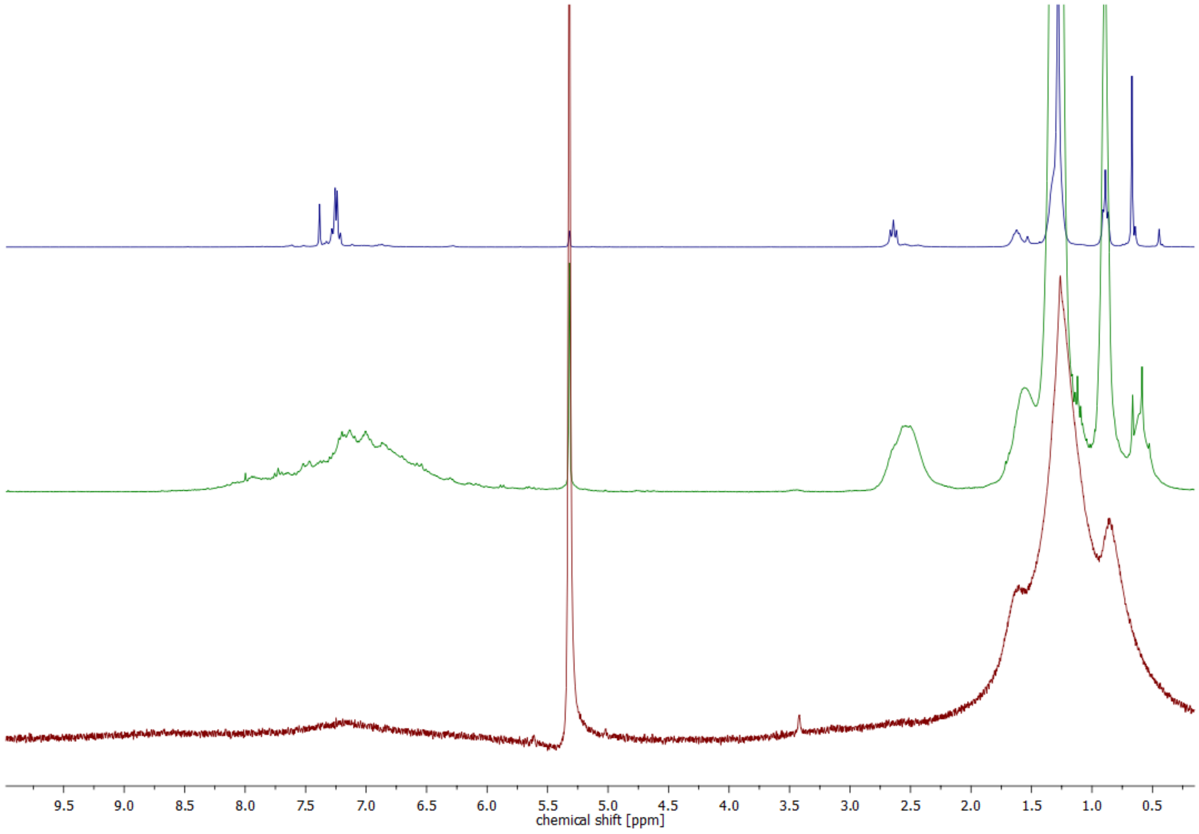

Figure S 37. ^{1}H-NMR comparison of building block **8** (top), **LTP** (middle) and **wGNR** (bottom) in CD_2Cl_2.

3 CV/SEC of compound 1

The cyclic voltammogram (CV) and square wave voltammetry (SWV) of **1** measured in dichlorometane shows two oxidations at the half-wave potentials of 0.59 and 0.71 V vs. Fc/Fc^+ (Figure S38a). The CVs measured at different scan rates show that the second oxidation process becomes irreversible at scan rate 10 mV/s and slower and an additional peak at ca.0.15 V appears in the backward potential scan (Figure S38b). This points that the follow-up chemical reaction take place after the second oxidation. The CV measured in tetrahydrofuran exhibits two reversible reductions at half-wave potentials of -2.48 and -2.54 V vs. Fc/Fc^+ (Figure S38a).

The absorption bands at 430, 480, 555, 698 and 1264 nm appear during the first oxidation (Figure S39a). Simultaneously, the EPR spectrum shows a single unresolved signal with a *g* value of 2.0028 and a line width of 4.5 G. The appearance of the EPR signal during the first oxidation confirms the formation of the radical cation. At the potentials of the second oxidation, a band at 838 nm is observed in the UV-Vis-NIR spectra and the EPR intensity starts to decrease. This indicates that an EPR-silent species, a dication, is formed. The evolution of the intensities of the spectroscopic features in dependence of the applied potential is shown in Figure S39b,c. In the backward potential scan, two reduction peaks at low potentials are obtained in the CV. Simultaneously, a weak EPR signal with a *g* value of 2.0026 is detected from 0.2 V indicating the formation of additional species formed after the second oxidation of **1**.

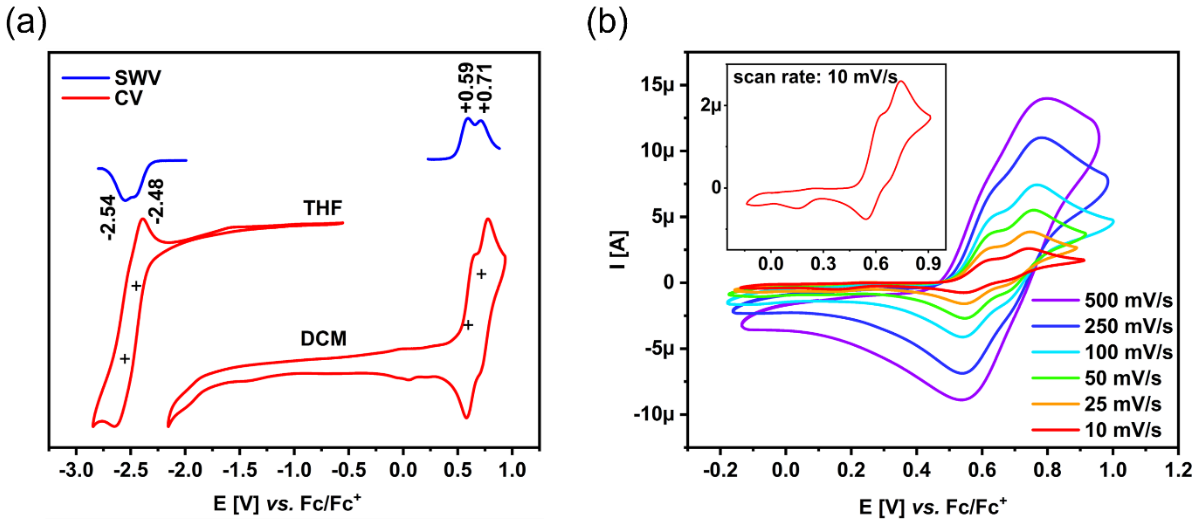

Figure S38. (a) Cyclic (black) and square wave (red) voltammogram of the compound **1** measured in dichloromethane and tetrahydrofuran for oxidation and reduction, respectively. Electrolyte salt: 0.1 M Bu_4NPF_6. (b) CVs of the compound **1** measured in dichloromethane at different scan rates.

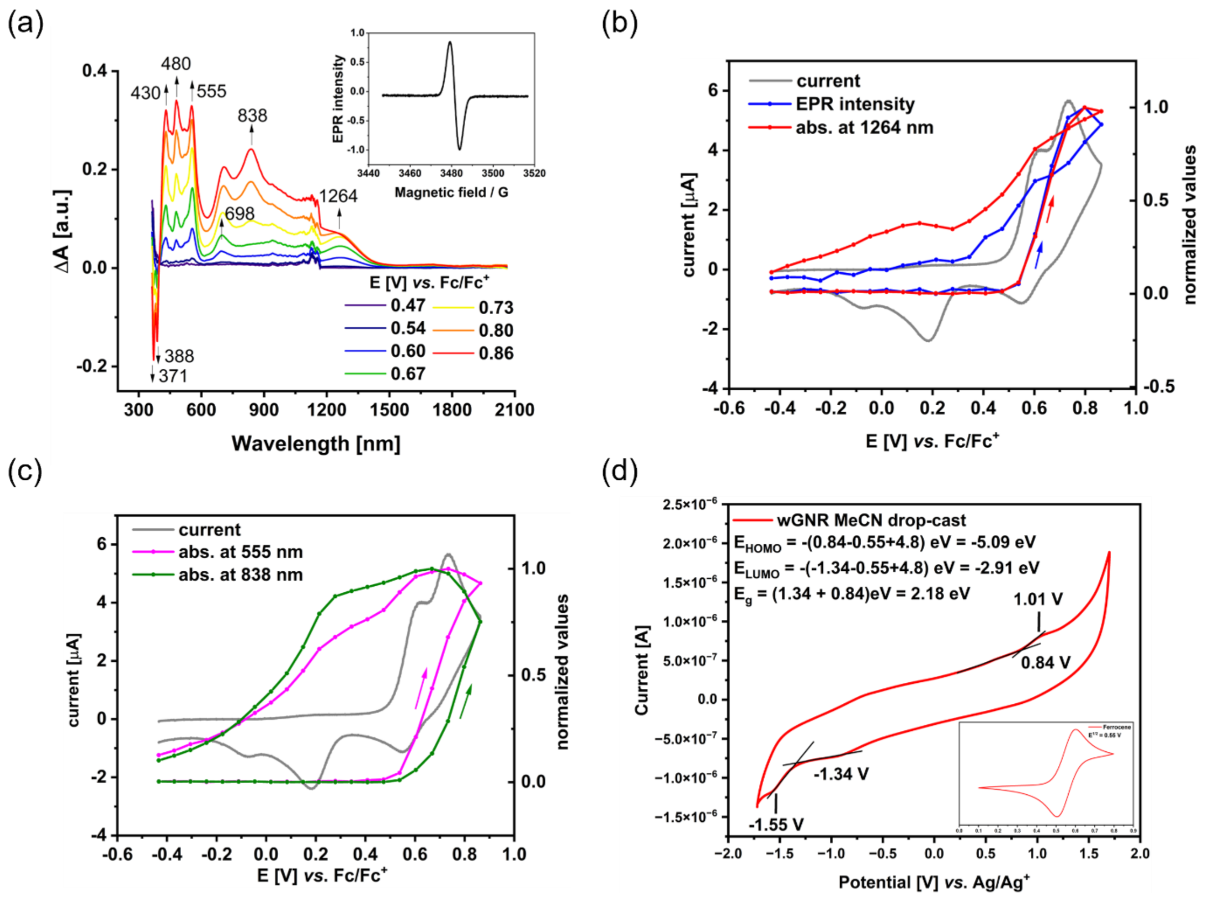

Figure S39. (a) In situ UV-vis-NIR spectra recorded during two oxidation steps. Inset: EPR spectrum of the radical cation. (b) Potential profiles of the intensities of the EPR signal. (c) Selected absorption bands obtained during the first and second oxidation. (d) CV of **wGNR** and the corresponding bandgap estimation.

4 Analytical Gel Permeation Chromatography (GPC) of LTP

After fractionating **LTP** in 4 fractions by preparative recycling GPC (Figure S40), the fractions were reinjected into an analytical GPC-device to determine their relative molecular mass distributions against polystyrene standards.

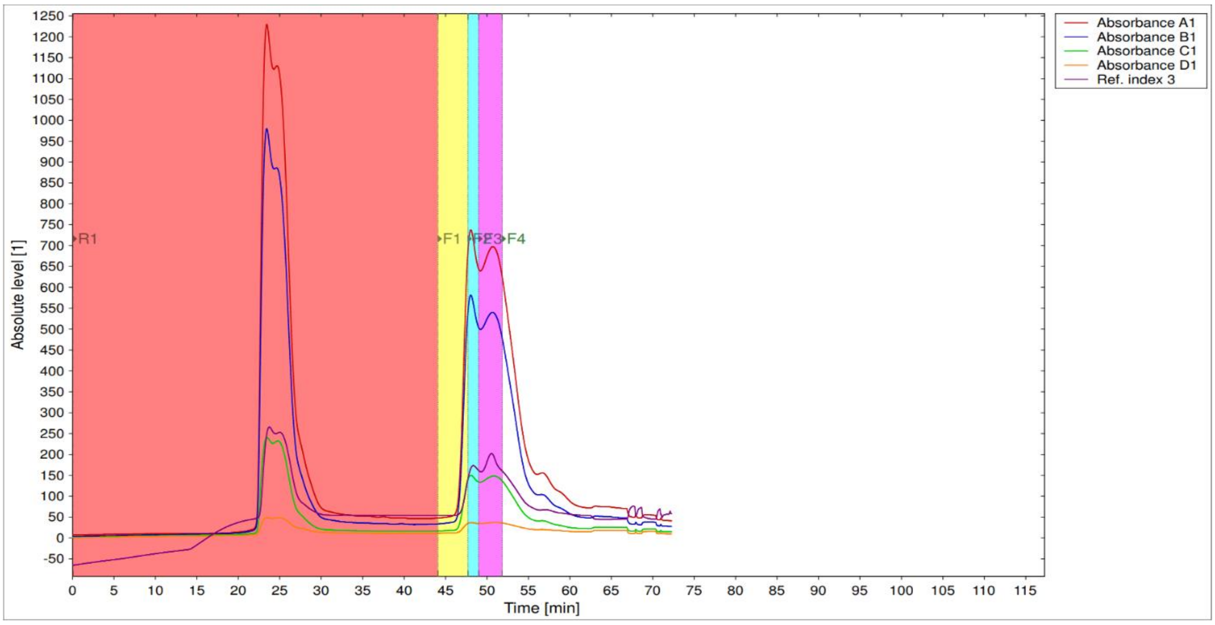

Figure S40. Recycling GPC chromatogram of **LTP** in chloroform. Collection during the second cycle.

Molecular Weight Averages

Peak	Mp	Mn	Mw	Mz	Mz+1	Mv	PD
Peak 1	23692	26436	30379	36764	46305	35574	1.149
Peak 2	785	710	747	784	820	779	1.052
Peak 3	339	339	348	357	366	356	1.027

Chromatogram

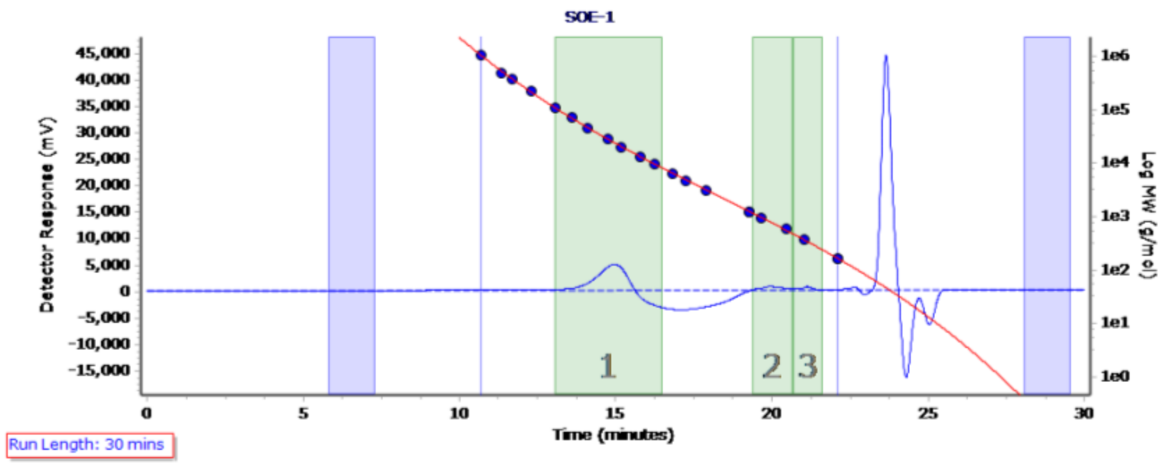

Figure S41. Analytical GPC results of **LTP**, fraction 1.

Molecular Weight Averages

Peak	Mp	Mn	Mw	Mz	Mz+1	Mv	PD
Peak 1	17370	18477	23265	37406	77727	33778	1.259
Peak 2	803	655	704	750	789	743	1.075

Chromatogram

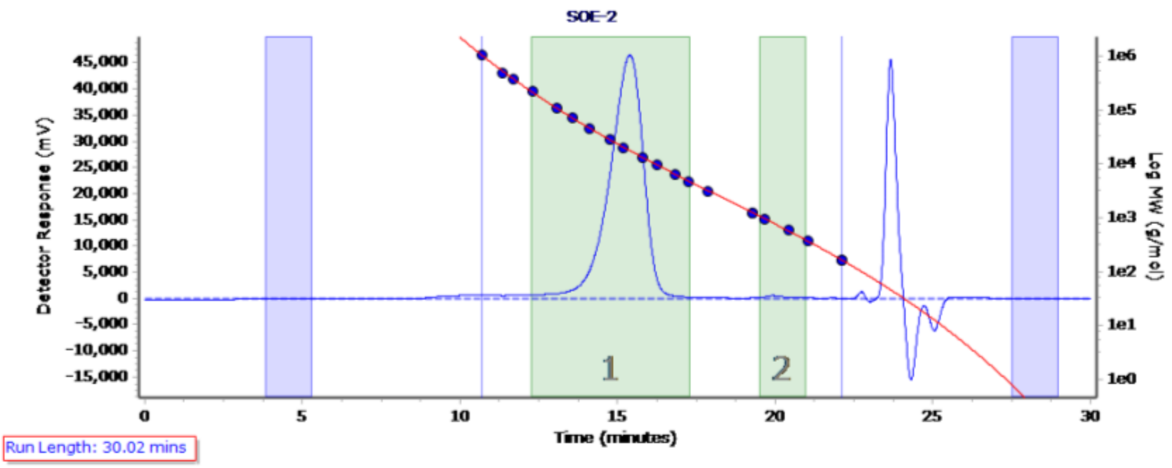

Figure S42. Analytical GPC results of **LTP**, fraction 2.

Molecular Weight Averages

Peak	Mp	Mn	Mw	Mz	Mz+1	Mv	PD
Peak 1	10215	10672	12298	15104	20137	14542	1.152

Chromatogram

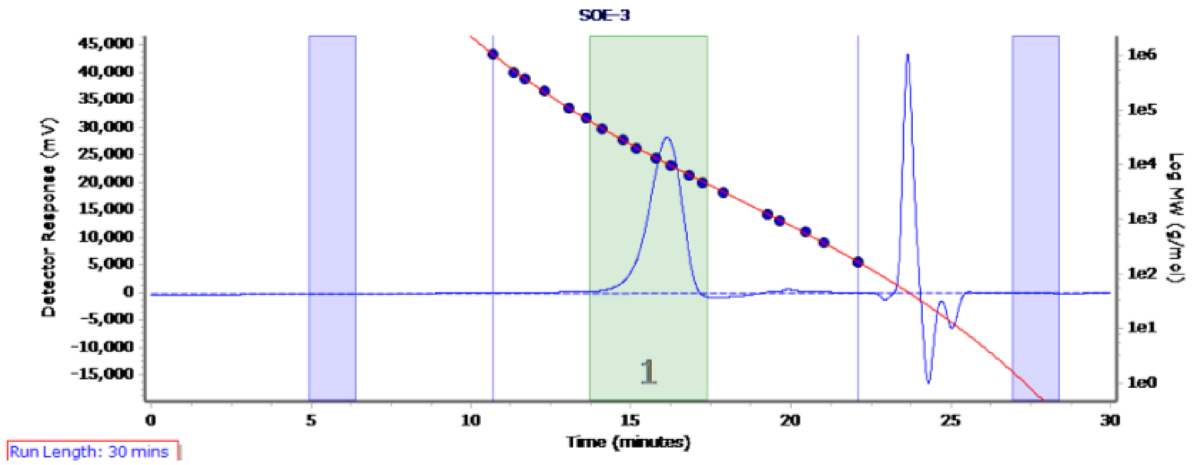

Figure S43. Analytical GPC results of **LTP**, fraction 3.

5 Raman spectroscopy of LTP and wGNR

Computational methods. To simulate the Raman spectra of **LTP** and **wGNR**, density functional theory (DFT) calculations were performed at the B3LYP/6-31G(d,p) level using the CRYSTAL17 software.[4] Molecular models of both **LTP** and **wGNR** were considered, with periodic boundary conditions applied in the longitudinal direction. To keep the computational burden under control, the long $C_{12}H_{25}$ alkyl chains were represented using methyl groups. These models were selected as those with the lowest energy after several conformers were carefully considered and are shown in Figure S44. Since DFT is known to overestimate normal frequencies of molecules, a scaling factor of 0.98 has been applied to the wavenumbers of the computed vibrational normal modes before plotting the Raman spectra. The minor discrepancies observed in the simulated Raman intensities of **wGNR** vs. the experimental spectrum are due to the resonance conditions reached by the 405 nm laser used in our setup (see Chapter 1) that are not considered by the CRYSTAL17 code.

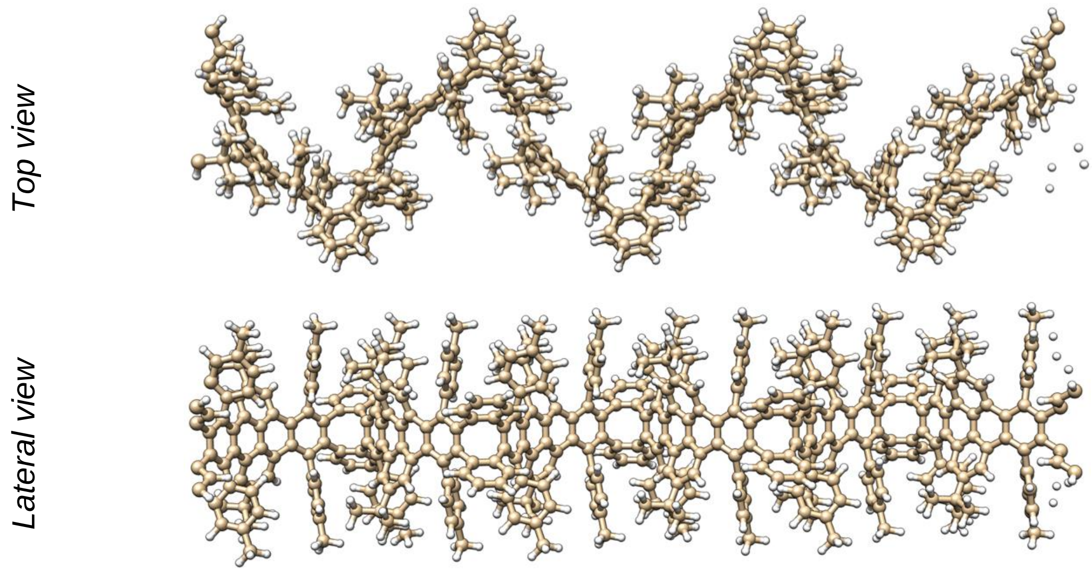

(a)

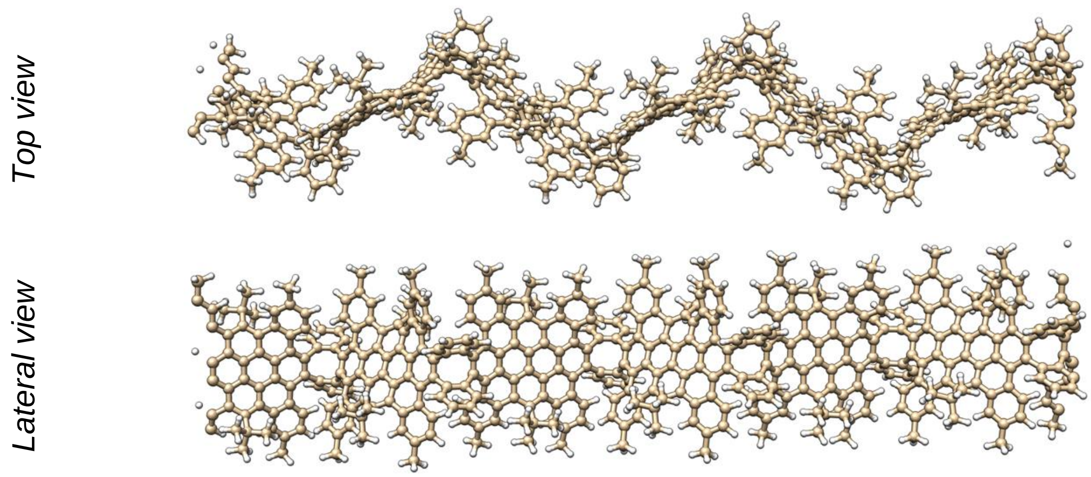

(b)

Figure S44. Lateral and top views of three repeat units of the periodic molecular models of (a) **LTP** and (b) **wGNR** used to simulate the Raman spectra shown in the main text.

Assignment of the Raman spectra of LTP and wGNR. Tables S1 and S2 report detailed assignments of selected bands of the Raman spectra of **LTP** and **wGNR** reported in Figures S45 and S47. For the sake of conciseness, we indicate with **O** the octagonal ring, with **B1** the aromatic ring condensed to the octagonal ring, and with **B2** the hexagonal ring bearing the $C_{12}H_{25}$ alkyl chains, as shown in Figures S45 and S47.

Table S1. Calculated, scaled, and experimental Raman frequencies along with their descriptions and Raman activity of **LTP**.

DFT wavenumber (cm^{-1})	Scaled wavenumber (cm^{-1})	Experimental wavenumber (cm^{-1})	Short description	Long description	Raman activity (Å^4/amu)
1252; 1253	1227; 1228	1222	**O**	CC stretching in **O** and in the two condensed rings	192; 65
1287; 1288; 1290	1261; 1262; 1264	1263	-	CC stretching in the methyl group, collective CH bending	354; 167; 167
1319; 1319	1293; 1293	1286	-	Collective CC stretching	302; 773
1336; 1336; 1336	1309; 1309; 1309	1307	-	CC stretching in the central CC bond of the pyrene moiety	561; 670; 204
1350; 1352; 1354	1323; 1325; 1327	1334	D peak	D-like deformation	2184; 5453; 1304
1422; 1423; 1423; 1423; 1424; 1424	1394; 1395; 1395; 1395; 1396; 1396	1394	Me	Me umbrella mode	12; 28; 8; 18; 74; 106
1452	1423	1421	-	in-plane CH bending in the graphenic region	371
1484	1454	1455	-	CC stretching in the center of the pyrene moiety and on sides of **O**	292
1527	1496	1488	-	transversal G-like deformation	604
1551; 1551	1520; 1520	1508	-	collective in-plane CH bending	94; 30
1619; 1621	1587; 1589	1567	**B1** and **B2**	G-like deformation in **B1** and **B2**	100; 239
1643	1610	1606	G peak	G-like deformation in the graphenic region (in the pyrene moiety)	1189
1650	1617	1606	**B1**	G-like deformation in **B1**	524

Table S2. Calculated, scaled, and experimental Raman frequencies along with their descriptions and Raman activity of **wGNR**.

**DFT wavenumber (cm^{-1})**	**Scaled wavenumber (cm^{-1})**	**Experimental wavenumber (cm^{-1})**	**Short description**	**Long description**	**Raman activity ($Å^4$/amu)**
1204; 1208	1180; 1184	1180	**B1** and **B2**	in-plane CH bending in **B1** and **B2**	710; 208
1285; 1288	1259; 1262	1251	**B1** and **O**	**O** deformation and CH bending in **B1**	3161; 5456
1303; 1306; 1308; 1310	1277; 1280; 1282; 1284	1289	**B2**	in-plane CH bending in **B2**	2855; 8923; 2389; 1738
1318; 1320; 1321; 1324; 1325	1292; 1294; 1295; 1298; 1299	1297	D peak	collective CC stretching and D mode	1827; 5571; 2763; 940; 1108
1357; 1361	1330; 1334	1335	-	CC stretching in the graphenic region	9145; 14360
1401	1373	1372	-	CC stretching in the graphenic region	556
1422; 1423	1394; 1395	1372	Me	Me umbrella	884; 1042
1476; 1477	1446; 1447	1445	G mode	transversal G-like deformation in the graphenic region	1852; 1526
1498; 1499	1468; 1469	1478	G mode	transversal G-like deformation in the graphenic region and Me deformation	271; 248
1515; 1517; 1519; 1519; 1519; 1522	1485; 1487; 1489; 1489; 1489; 1492	1478	tBu	tBu deformation and CC stretching between **B1** and **O**	682; 816; 515; 434; 406; 883

1545; 1546	1514; 1515	1515	**B2**	in-plane CC stretching and in-plane CH bending in **B2**	319; 279
1595; 1597	1563; 1565	1608	G peak	transversal G deformation in the graphenic region	7704; 2480
1615; 1616	1583; 1584	1608	G peak	transversal G deformation in the graphenic region	929; 3204
1642; 1644; 1645; 1645	1609; 1611; 1612; 1612	1608	G peak	G-like deformation in **B1**	195; 153; 203; 592
1662; 1667	1629; 1634	1608	G peak	G-like deformation in **B2**	1127; 1263

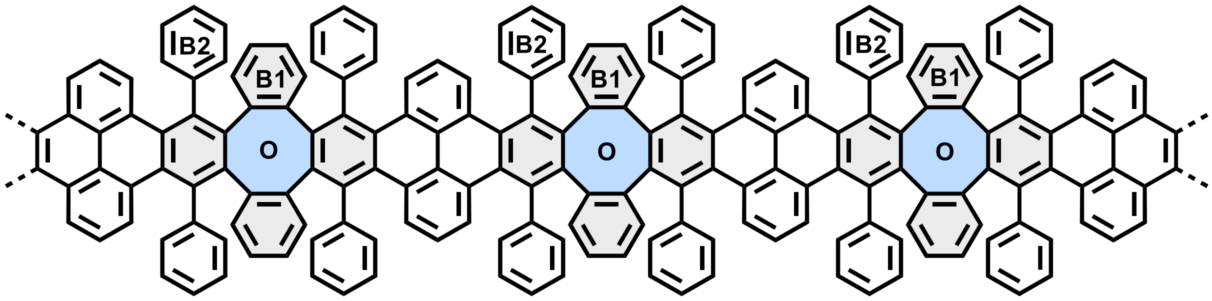

Figure S45. Labels for **LTP**, used in the assignment of Table S1.

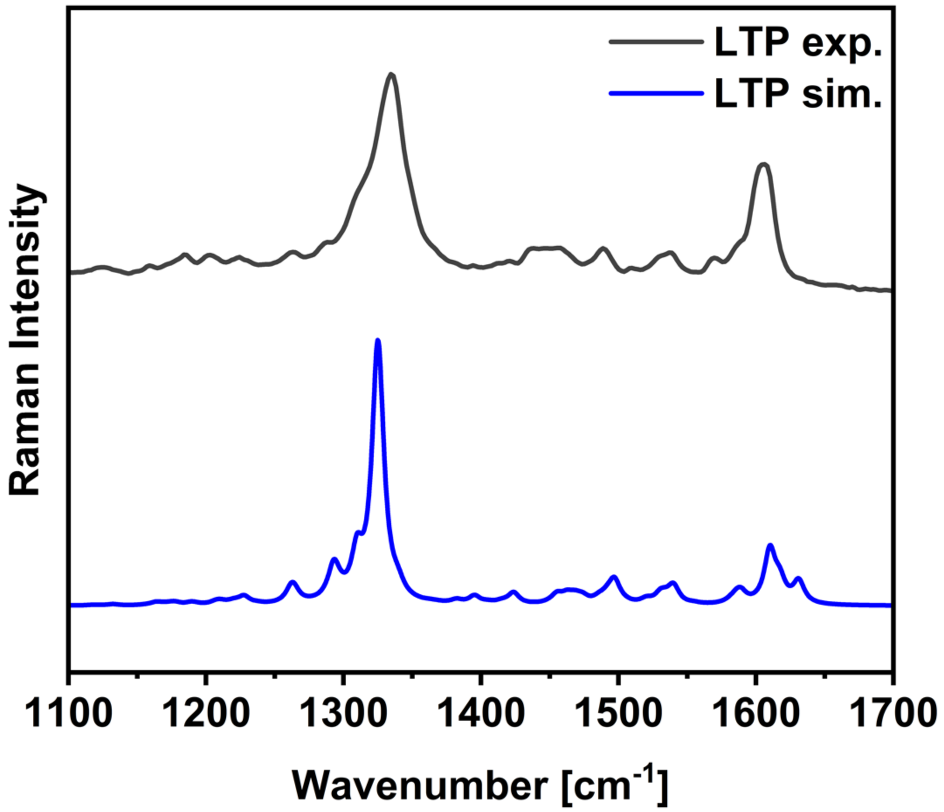

Figure S46. Experimental and simulated Raman spectra of **LTP**.

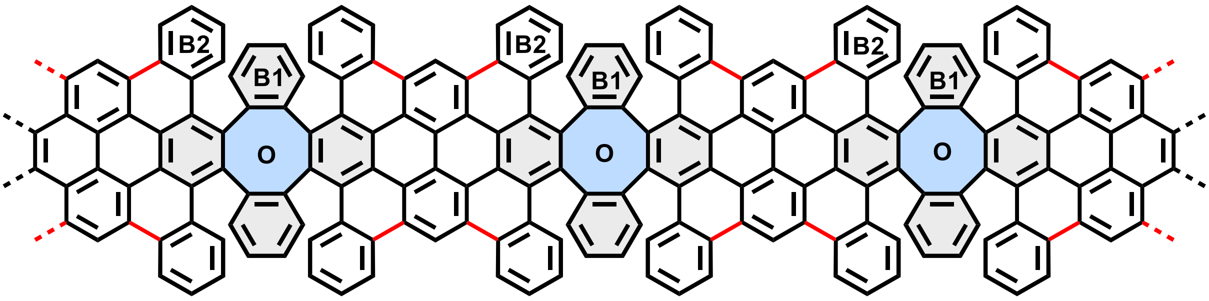

Figure S47. Labels for **wGNR**, used in the assignment of Table S2.

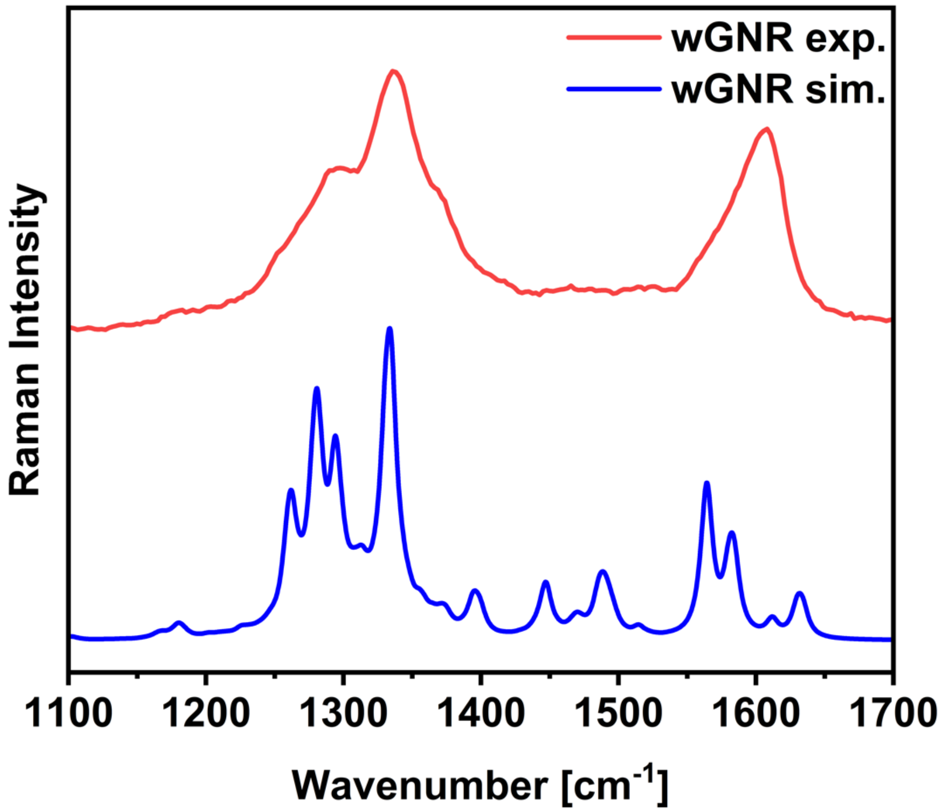

Figure S48. Experimental and simulated Raman spectra of **wGNR**.

6 FT-IR spectroscopy of 7, 1, LTP and wGNR

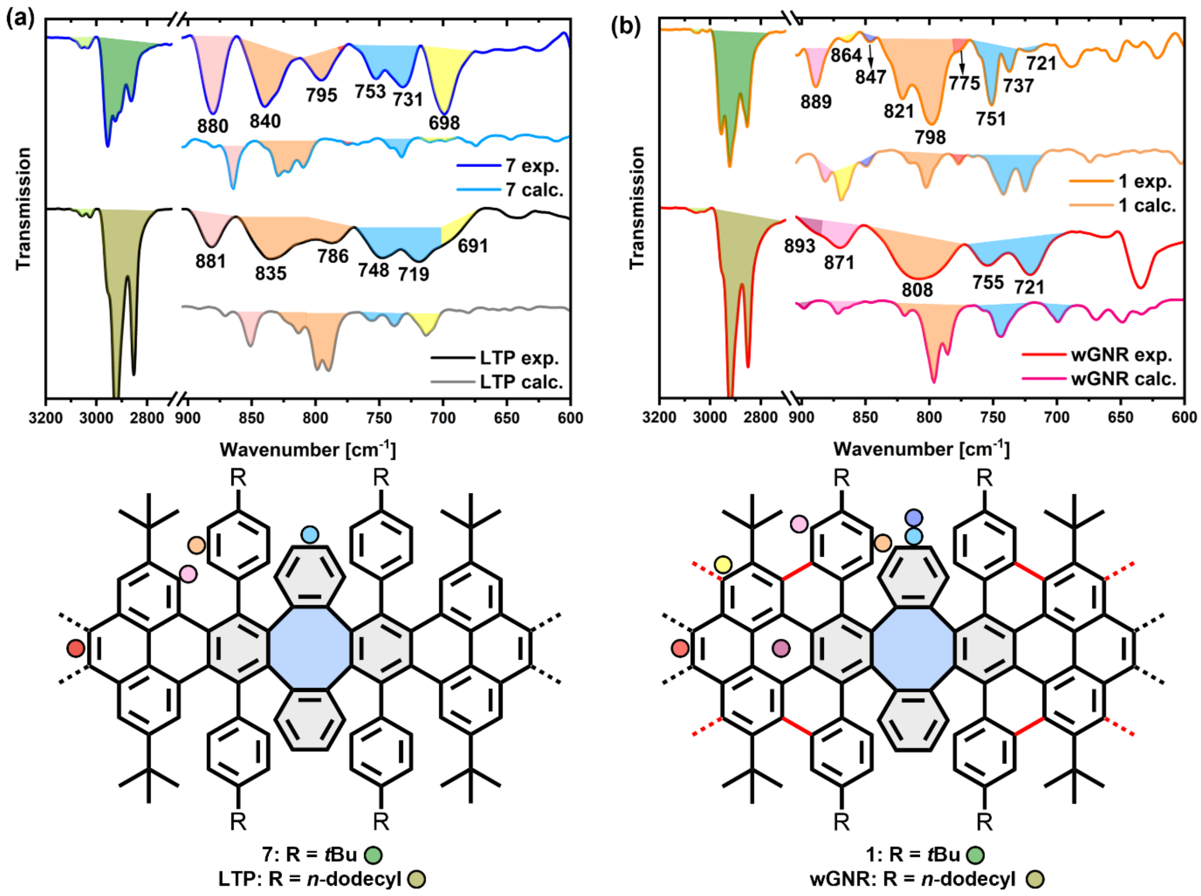

Figure S 49. Experimental and simulated IR spectra of **7**, **1**, **LTP** and **wGNR**.

Table S3. Simulated and experimental vibrations in the fingerprint region of compound **7**.

DFT frequency [cm^{-1}]	DFT frequency scaled [cm^{-1}]	Experimental frequency [cm^{-1}]	description
909	864	880	SOLO mode pyrene
873	828	840	DUO mode B2
816	776	795	wagging pyrene K-region
779	743	753	wagging B1
770	732	731	wagging B1 + out of plane vibration terphenyl (B2)

709	673	698	wagging B1 + in plane scaffold vibration pyrene
605	576	588	out of plane scaffold vibrations

Table S4. Simulated and experimental vibrations in the fingerprint region of compound **1**.

DFT frequency [cm^{-1}]	DFT frequency scaled [cm^{-1}]	Experimental frequency [cm^{-1}]	description
926	880	889	SOLO mode B2
914	866	864	SOLO mode pyrene
891	848	847	antisymmetric wagging of B1
858	816	821	DUO mode B2 + in plane scaffold vibration
845	804	798	DUO mode B2
817	777	775	DUO mode pyrene K-region
779	740	751, 737	wagging modes B1
762	725	721	wagging B1 + in plane scaffold vibrations
707	672	689	in plane scaffold vibrations

Table S5. Simulated and experimental vibrations in the fingerprint region of **LTP**.

DFT frequency [cm^{-1}]	DFT frequency scaled [cm^{-1}]	Experimental frequency [cm^{-1}]	description
937	891	881	antisymmetric pyrene SOLO mode
915	870	-	artifact from the calculation, ring breathing of the terminal benzenes
894	850	881	symmetric pyrene SOLO mode
864	822	835	asymmetric DUO mode B2
855	813	835-786	symmetric DUO modes B2 coupled with scaffold vibrations
839	798		
829	789		
794	756	748	wagging B1 and scaffold vibration
777	739	719	wagging B1
749	713	691	scaffold vibration of the pyrene + wagging B1

Table S6. Simulated and experimental vibrations in the fingerprint region of **wGNR**.

DFT frequency [cm^{-1}]	DFT frequency scaled [cm^{-1}]	Experimental frequency [cm^{-1}]	description
944	898	893	ring breathing mode tetrabenzoovalene
916	871	871	SOLO mode B2
860	818	808	ring breathing mode tetrabenzoovalene
837	796	808	DUO mode B2
825	785	808	DUO mode B2
797	758	755	Wagging B1
782	744	721	Wagging B1 + vibration octagon
736	700	689	Wagging B1 + out of plane scaffold vibration

8 X-Ray crystallographic analysis

8.1 Compound 7

X-ray diffraction data collection was carried out at the BESSY storage ring (BL14.2, Berlin-Adlershof, Germany).[5] XDSAPP2.0 suite was employed for data processing.[6,7] The structure was solved by direct methods and refined by SHELXL-2018.[8] Hydrogen atoms were added geometrically and refined with a riding model. The structure has been deposited under the CCDC reference 2284578.

```
Bond precision:   C-C = 0.0021 A          Wavelength=0.79990

Cell:              a=35.020(7)      b=15.760(3)        c=52.03(1)
                   alpha=90         beta=95.41(3)      gamma=90
Temperature:       100 K

                 Calculated                 Reported
Volume           28588(10)                  28588(10)
Space group      C 2/c                      C 2/c
Hall group       -C 2yc                     -C 2yc
Moiety formula   C108 H108                  ;C162H162;
Sum formula      C108 H108                  C162 H162
Mr               1405.95                    2108.91
Dx,g cm-3        0.980                      0.980
Z                12                         8
Mu (mm-1)        0.069                      0.071
F000             9072.0                     9072.0
F000'            9076.52
h,k,lmax         52,23,77                   49,22,62
Nref             49748                      40702
Tmin,Tmax        0.993,0.994
Tmin'            0.993

Correction method= Not given

Data completeness= 0.818          Theta(max)= 36.640

R(reflections)= 0.0638( 30631)    wR2(reflections)= 0.1982( 40702)

S = 1.073                 Npar= 1588
```

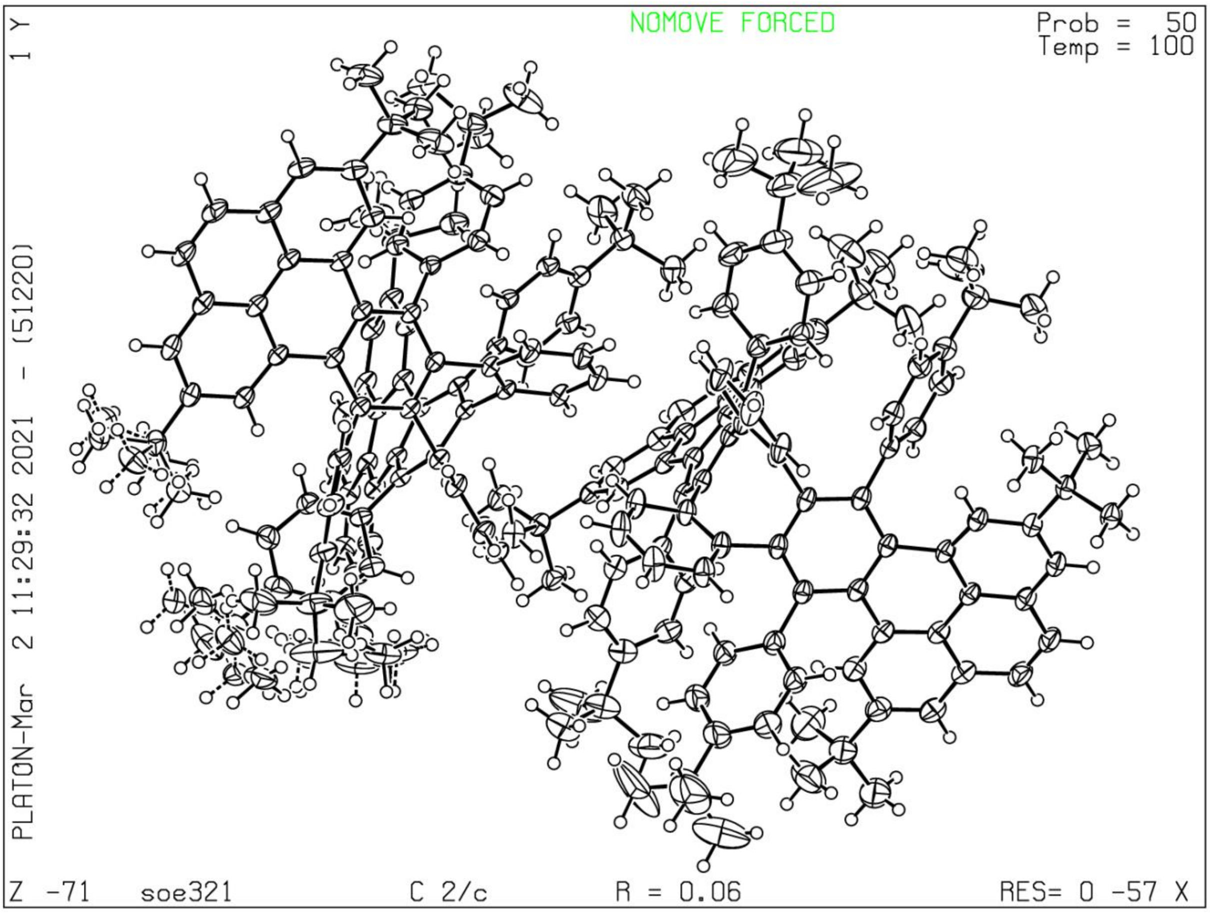

Figure S50. Crystal structure of compound **7**.

8.2 Compound 1

X-ray diffraction data collection was carried out at the BESSY storage ring (BL14.2, Berlin-Adlershof, Germany).[5] XDSAPP2.0 suite was employed for data processing.[6,7] The structure was solved by direct methods and refined by SHELXL-2018.[8] Hydrogen atoms were added geometrically and refined with a riding model. The structure has been deposited under the CCDC reference 2284577.

```
Bond precision:    C-C = 0.0032 A           Wavelength=0.79990

Cell:              a=15.040(3)        b=15.790(3)        c=19.740(4)
                   alpha=108.28(3)    beta=102.34(3)     gamma=102.35(3)
Temperature:       100 K

                    Calculated                   Reported
Volume              4144(2)                      4144.3(17)
Space group         P -1                         P -1
Hall group          -P 1                         -P 1
Moiety formula      C108 H100                    ?
Sum formula         C108 H100                    C108 H100
Mr                  1397.89                      1397.87
Dx,g cm-3           1.120                        1.120
Z                   2                            2
Mu (mm-1)           0.079                        0.081
F000                1496.0                       1496.0
F000'               1496.75
h,k,lmax            22,23,29                     20,22,27
Nref                28897                        22771
Tmin,Tmax           0.994,0.996
Tmin'               0.994

Correction method= Not given

Data completeness= 0.788            Theta(max)= 36.662

R(reflections)= 0.0767( 17029)                     wR2(reflections)=
                                                   0.2434( 22771)
S = 1.036                   Npar= 997
```

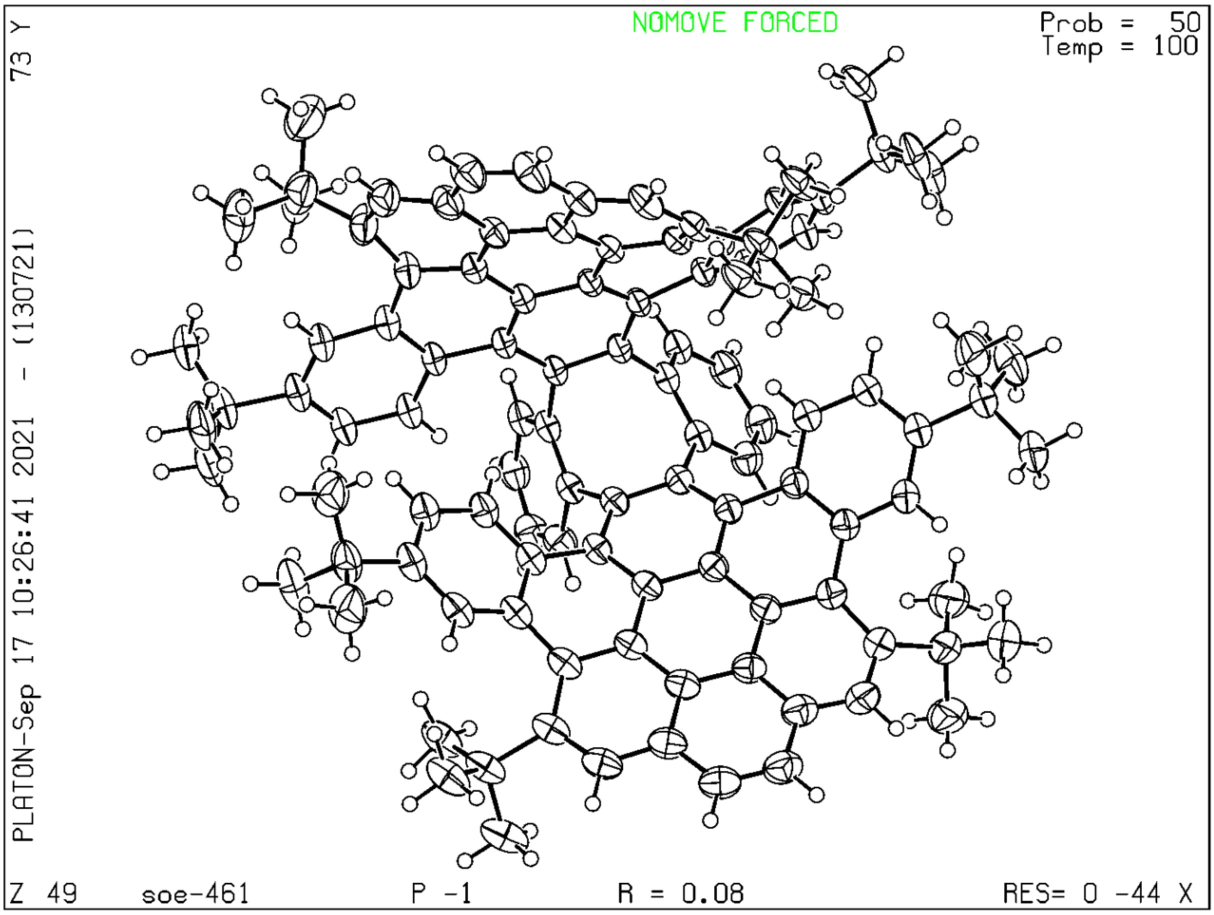

Figure S51. Crystal structure of compound **1**.

9 DFT Calculation Details

9.1 Simulation of the infrared spectra

For the simulation of the IR spectra of compound **7**, **1**, **LTP** and **wGNR**, the corresponding structures (Figure S52) were optimized on an HSEh1PBE/6-31G(d) level of theory with the Gaussian 16 software package.[9] Subsequently, frequency analysis (# freq hseh1pbe/6-31g(d)) was run on the same level of theory. **LTP** and **wGNR** were represented by fragments of two repetition units (Figure S53). To save calculation time while retaining a realistic substitution pattern, the aliphatic side chains of **LTP** and **wGNR** were replaced by methyl groups. The obtained spectra were read from the .log file by the Multiwfn console application[10] and scaled by factor 0.951. XYZ-files of the optimized structures can be found attached.

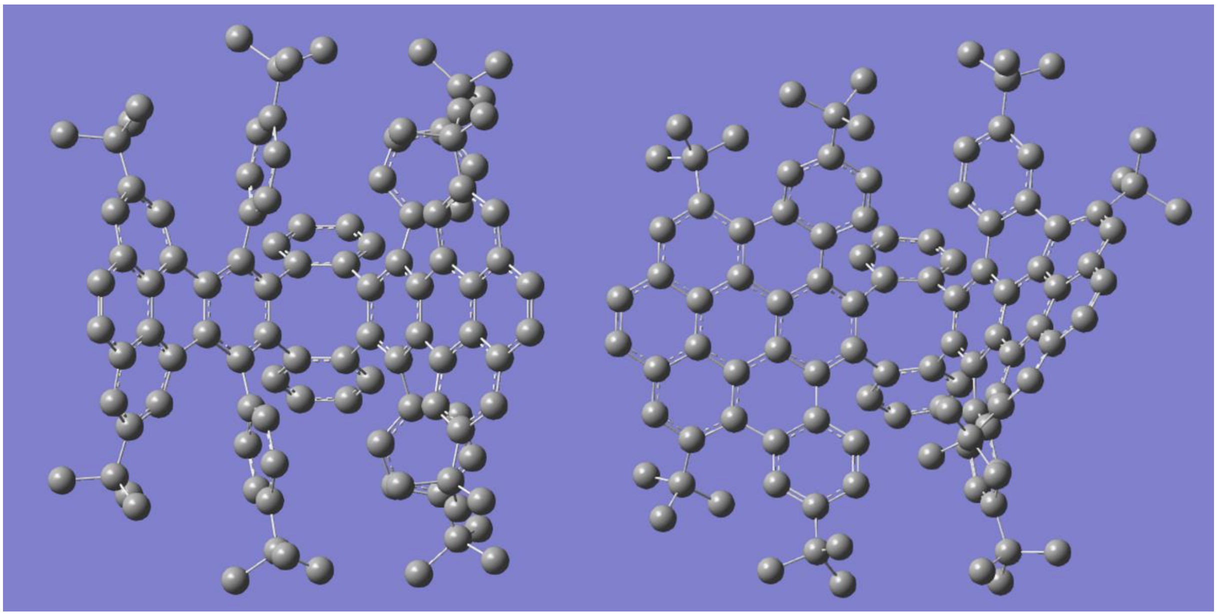

Figure S52. DFT-optimized structures of compound **7** (left) and **1** (right).

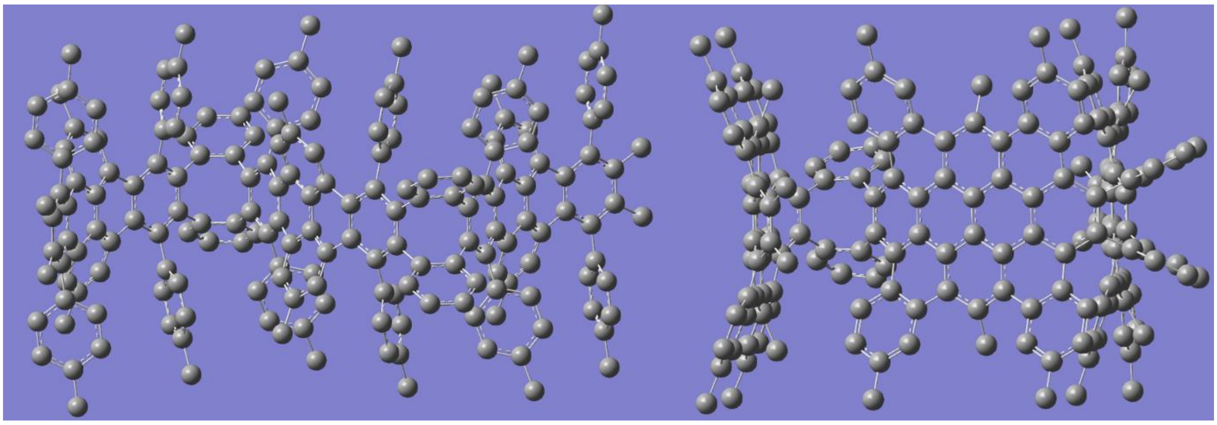

Figure S53. Optimized geometries of an **LTP**-fragment (left) and **wGNR**-fragment (right). Aliphatic side chains are represented by methyl groups to save calculation time.

9.2 Band gap and UV-Vis calculation

The density functional theory (DFT) calculation for **wGNR** were performed by using the Quantum ESPRESSO 6.7 software. The Generalized gradient approximation (GGA) based on the Perdew–Burke–Ernzerhof (PBE) functional was used for the exchange-correlation energy. The projector augmented wave (PAW) method was used to describe the ionic core pseudopotential. A plane wave energy cutoff of 60 Ry was used, and a Monkhorst k-mesh was chosen as 3 × 1 × 1. All structures were fully relaxed until the total force got smaller than 0.0013 eV/Å in the bulk of the GNRs. Figure S54 shows the calculated band structure of **wGNR**. For the UV-Vis simulations, the B3LYP functional with Grimme's D3 correction (Becke–Johnson damping) was used for geometry optimization in the ground state. The 6-31G(d) basis set was used. All geometry optimization was done in the gas phase and based on the single crystal structures. To simulate the UV-Vis spectra of the molecules TD-DFT calculations were carried out using the B3LYP functional and the 6-31G(d) basis set. For better comparison to the experimental absorption spectra, the polarity of the solvent dichloromethane was added.

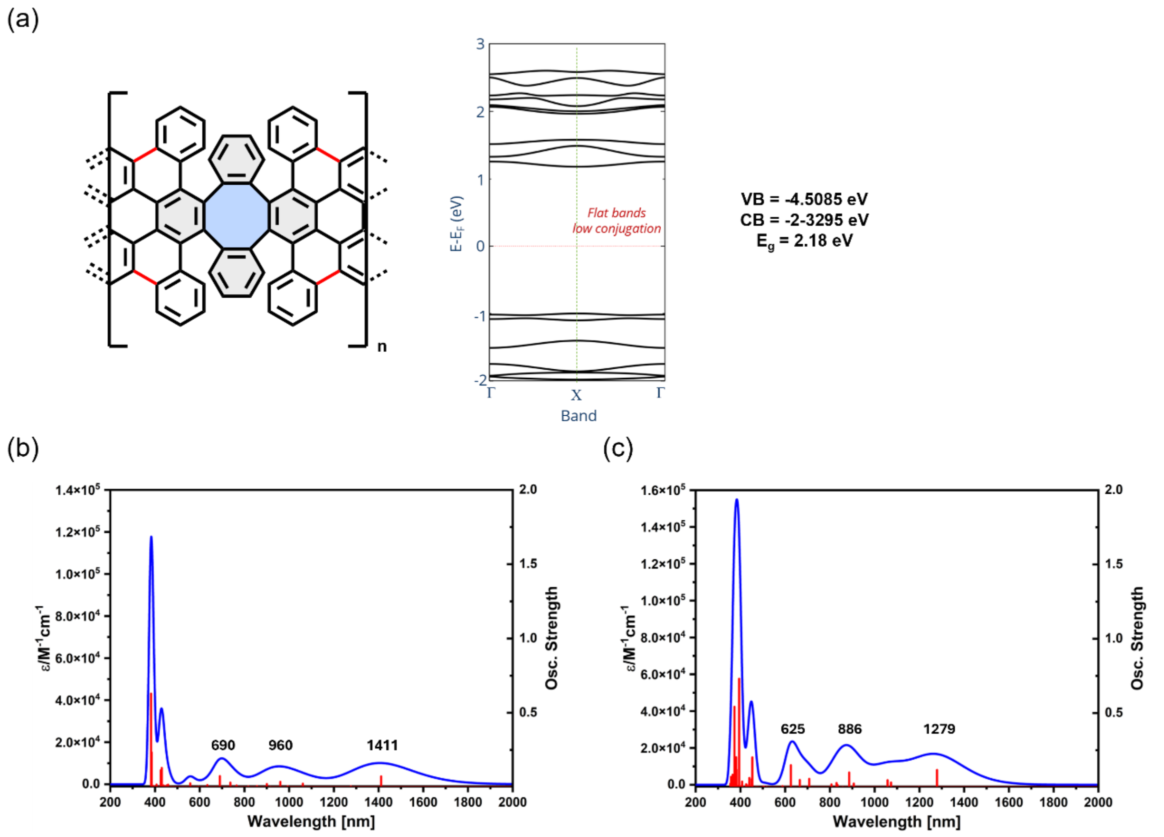

Figure S54. (a) chemical repetition unit, band structure and energy values for wGNR. (b) TD-DFT-simulated UV-Vis for the radical cation of model compound **1**, as well as (c) the UV-Vis simulation of the dication of **1** on a B3LYP/6-31G(d) level of theory.

10 GOLEC fabrication

Host and guest solution preparation:

The host solution was prepared by separately dissolving the materials below in chlorobenzene in a concentration of 30 mg/mL by stirring at (50 ˚C) for 4 h. The guest solution was prepared by dissolving **wGNR** in tetrahydrofuran with a concentration of 10 mg ml^{-1}. The reference SYPPV emitter was prepared by dissolving in chrolobenzene with a concentration of 7 mg ml^{-1} in a nitrogen filled glovebox, heated at 70˚C by stirring overnight. A blend of the high-molecular weight polymer poly (*N*-vinylcarbazole) (PVK, M_w: 40 000, Polysciences) and 1,3-bis[2-(4-tert-butylphenyl)-1,3,4-oxa- diazo-5-yl] benzene (OXD-7, BLD Pharmatech) in a 1:1 mass ratio was used as the host. Tetrahexylammonium tetrafluoroborate ($THABF_4$, Sigma-Aldrich) was dissolved into the host-guest solution as the conductive electrolyte.

Device fabrication:

Glass substrates with pre-patterned indium-tin oxide were firstly cleaned by detergent solution. Substrates were then soaked in de-ionized water for about 5 min, followed by ultrasonic treatments within acetone and isopropanol for 5 min, respectively. These substrates were then blown dry by nitrogen and heated for 5 min at 140 °C in an oven. Afterwards, ITO substrates were moved to a UV–ozone reactor for another 20 min of treatment. A layer of 45 nm PEDOT:PSS (AI 4083) was spin-coated and thermally treated at 140 °C for 10 min under ambient atmosphere. The emissive films were spin-coated on top of PEDOT:PSS in a nitrogen-filled glove box. The layer of barium and aluminum were deposited in a vacuum chamber with a pressure of 10^{-7} mbar.

Device characterization:

The current density-voltage characteristic of these OLECs was measured with a Keithley 2400 source meter, while the photocurrent was recorded simultaneously with a calibrated Si photodiode, which is close to the emissive pixel, but not touching it. Photocurrent-voltage characteristics were measured in a dark environment. The EL spectra were detected with a USB4000-UV-VISES spectrometer.

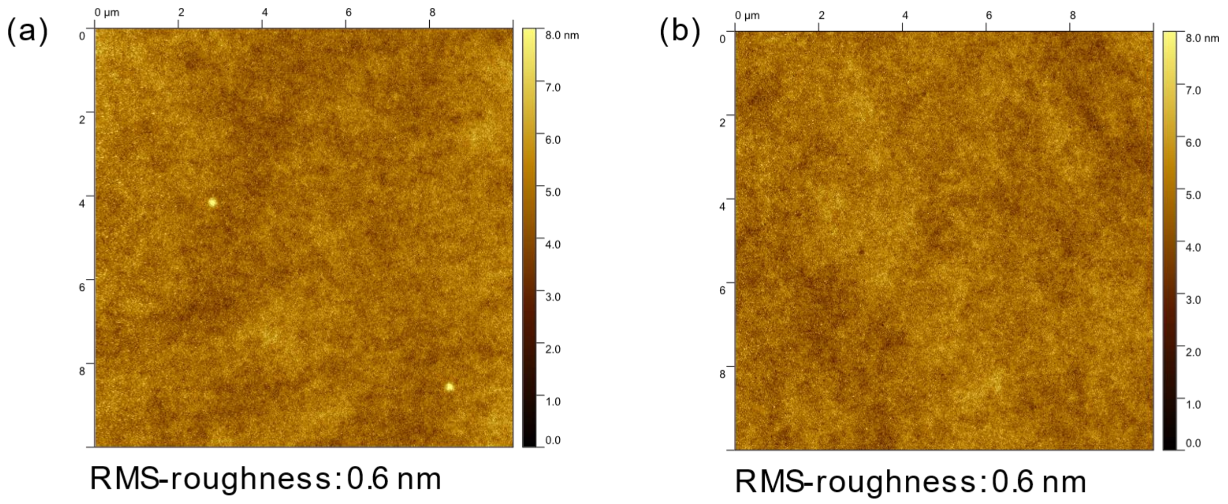

Figure S55. Atomic force microscopy images of (a) PVK: OXD-7: $THABF_4$ host film, (b) PVK: OXD-7: $THABF_4$:**wGNR** host-guest film.

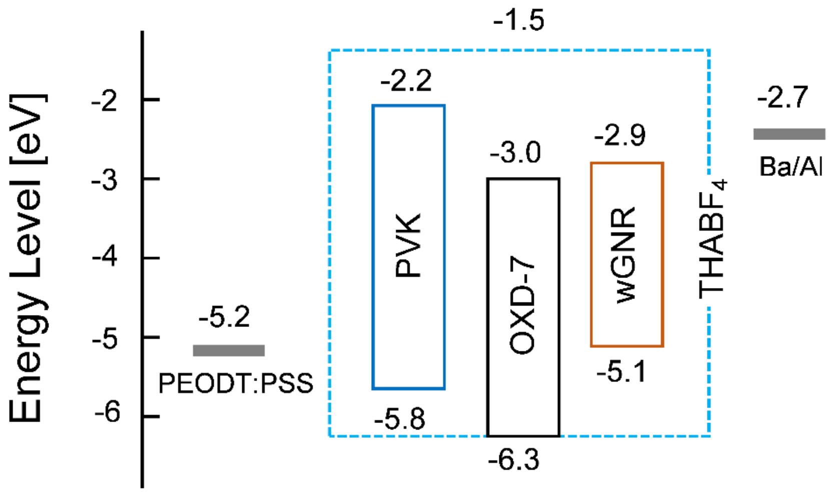

Figure S56. Energy diagram for different OLEC devices.

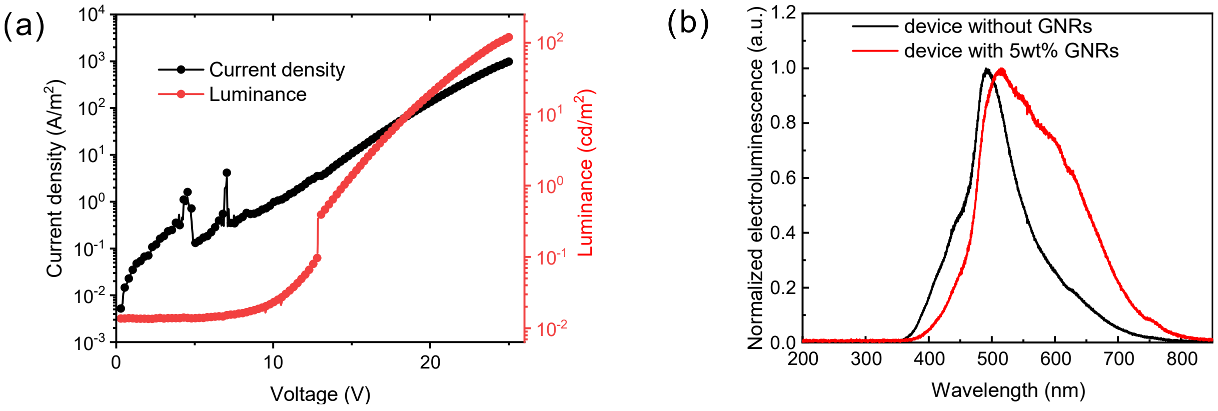

Figure S57. (a) Current density and luminance vs voltage for the reference device without **wGNR**, (b) EL spectra for reference device and GOLECs with 5 wt% **wGNR**.

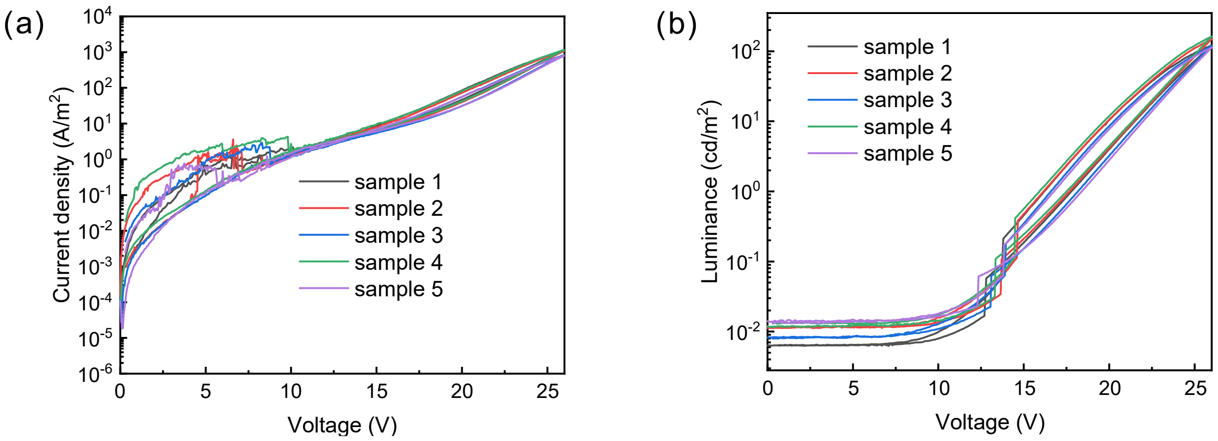

Figure S58. Device reproducibility experiments. (a) Current density and (b) luminance vs voltage for different GOLEC devices with 5wt% **wGNR**.

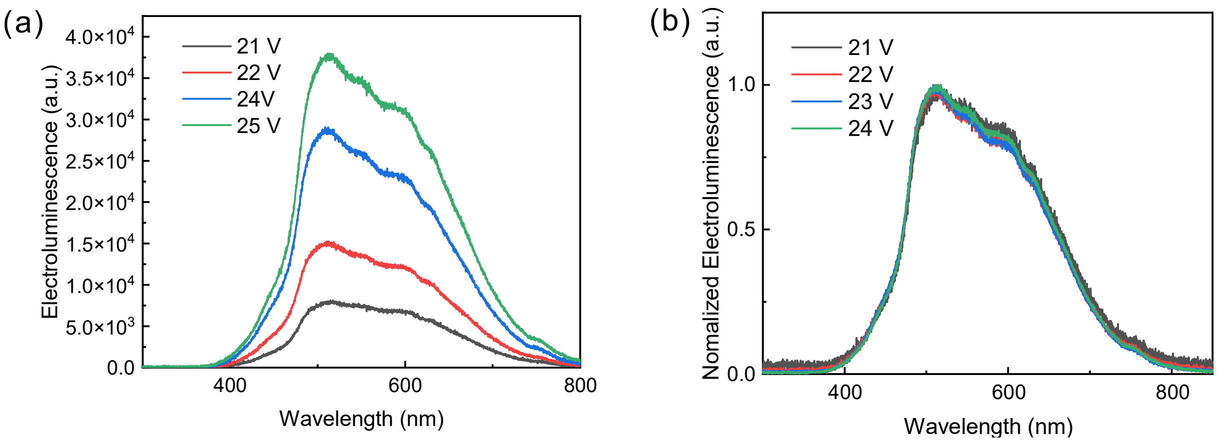

Figure S59. (a) EL spectra and (b) Normalized EL spectra of GOLEC with 5wt% **wGNR** under different voltages.

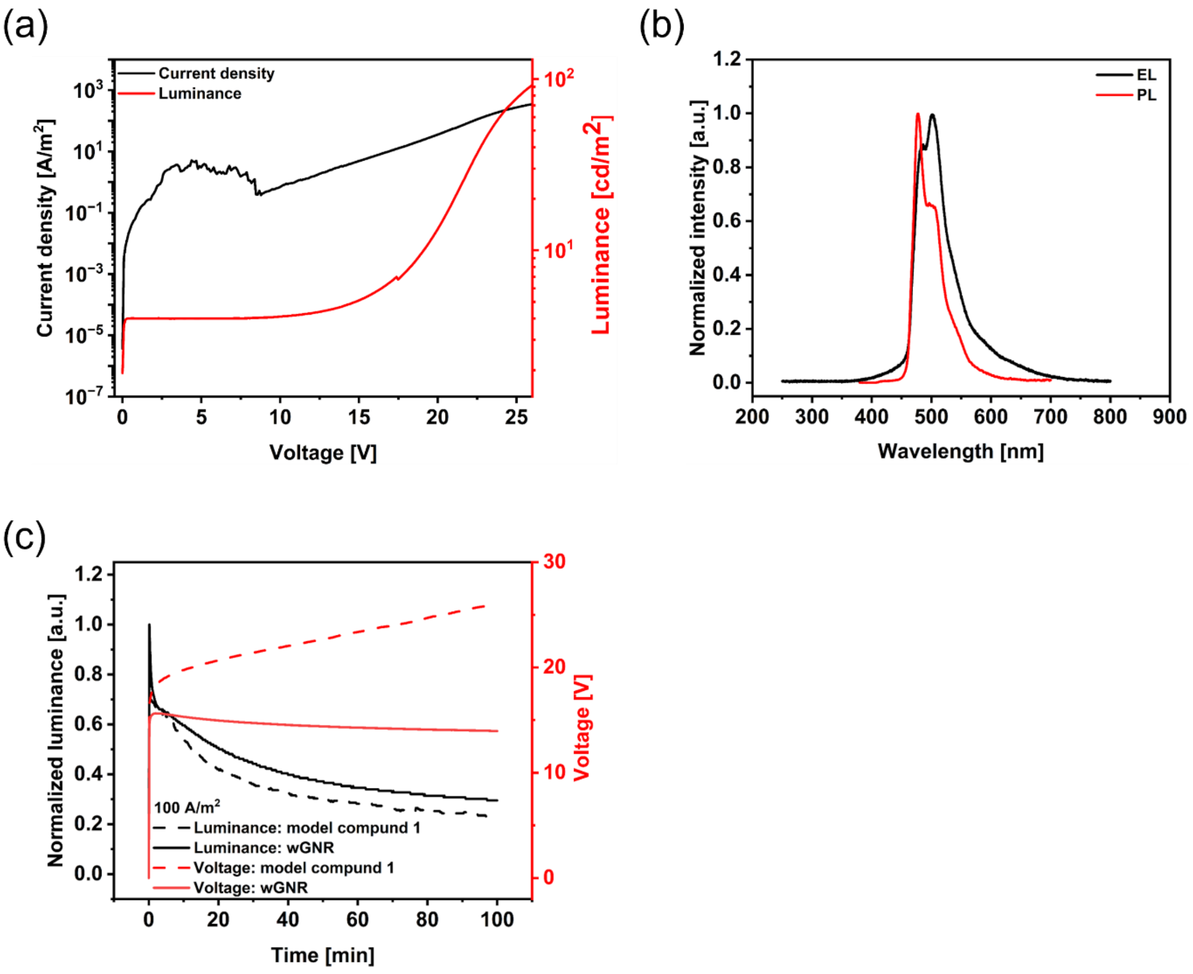

Figure S60. Device data of OLECs with model compound **1**. (a) Current density and luminance against driving voltages for OLECs with 5 wt% of model compund **1**. (b) Comparision of EL and PL spectra of model compound **1**. The EL spectrum was obtained from OLECs with 5 wt% of model compund **1**, while the PL spectrum was measured in toluene. (c) Luminance and voltage versus aging time for OLECs with 5 wt% **wGNR** and model compund **1** under a current density at 100 A/m^2.

11 Solid state NMR

The precursor **LTP** and **wGNR** were further analysed by a combination of solid-state ^{1}H and ^{13}C{^{1}H} MAS NMR. High spinning speeds of 62.5 kHz at a field of 11.76 T were chosen for high resolution. The quantitative ^{1}H MAS NMR single pulse spectrum with a repetition delay of 16 s of **LTP** is depicted in Figure S61. Here Figure S61a shows well separated aromatic and aliphatic regions. Each of the regions consists of several broader signals suffering from stronger overlap. Deconvolution reveals aliphatic signals located around −0.6 ppm, 0.8 ppm, 1.4 ppm and 2.4 ppm and aromatic signals around 5.0 ppm, 6.7 ppm and 7.2 ppm. It is noted that deconvolution of strongly overlapping signals is often ambiguous to some extent. Analytical integration of the deconvoluted signals (including the spinning sidebands) results in a ratio of aliphatic to aromatic protons of 82.2:17.8, which matches the theoretical value of 80.8:19.2

calculated from the structure within the expected error margin. Figure S61a also includes the ^{1}H Hahn-Echo MAS NMR spectrum with an echo time (excitation to acquisition) of 32 µs. The Hahn echo is free of baseline distortions and probe background signal. Intensities, however, might suffer from distortions due to T_2 relaxation. Nevertheless, deconvolution of the echo spectrum reveals an aliphatic to aromatic proton ratio of 80.8:19.2 matching the theoretical value exactly. Upon graphitization the ^{1}H linewidth increases as is seen by the ^{1}H MAS NMR spectrum of **wGNR** in Figure S61 (b). The line broadening is attributed to the effects of the extended π-system and stronger effective dipolar coupling because of decreased mobility. This spectrum can be deconvoluted with peaks corresponding to aliphatic proton signals at 0.0 ppm, 0.9 ppm and 2.9 ppm and those corresponding to aromatic proton signals at 6.8 ppm and 8.6 ppm. Integration results in a ratio of 84.4:15.6 for aliphatic to aromatic protons, again close to the theoretical ratio of 85.5:14.4. Similarly, a ratio of 82.8:17.2 is found from the Hahn-echo NMR experiment. The larger error here is reasonable as more pronounced relaxation effects are expected for the more strongly dipolar coupled system present in **wGNR**. The higher fraction of aliphatic protons compared to the precursor **LTP** is a consequence of graphitization.

The spatial connectivity of the ^{1}H spin network can be examined using 2D double quantum / single quantum (DQ/SQ) NMR and the corresponding 2D NMR spectra for **LTP** and **wGNR** are shown in Figure S62. The 2D DQ/SQ spectra of **LTP** show well-resolved correlation signals even for very short recoupling times that probe for short-distance connectivity. This is to be expected as aliphatic and aromatic protons are spatially close. For **wGNR**, the 2D DQ/SQ NMR spectra show broad autocorrelation signals for the aliphatic and aromatic protons that are broadened along the diagonal spanned from approx. 2.7 ppm/5.4 ppm (δ_{SQ}/δ_{DQ}) to −3.0 ppm/−6.0 ppm and 10.7 ppm/21.4 ppm to 5.4 ppm/10.8 ppm, respectively. Also, the cross-correlation signals at approx. 7.1 ppm/8.0 ppm and 0.9 ppm/8.0 ppm are visible even for the shortest DQ recoupling times, again because of the close spatial proximity of at least some aliphatic and aromatic proton species. The broadening along the diagonal suggests a distribution of chemical shifts as a result packing effects present in the solid-state.

The same trend already observed in the 1D ^{1}H MAS NMR spectra can be seen in the ^{13}C{^{1}H} CP/MAS NMR spectra which are shown in Figure S63. A short relaxation delay

of 3.5 s was used for both samples. Upon graphitization, the ^{13}C linewidth increases because of extended π-conjugation and the more rigid structure. In this case, however, 3.5 s is not enough to allow for full relaxation neither for the precursor or GNR as experiments with a longer relaxation delay show, contrary what to what has been observed for other GNRs.[11] Hence, quantitative $^{13}C\{^1H\}$ CP/MAS NMR spectra could not be acquired in reasonable experimental time.

Experimental Details Solid-state NMR spectra were acquired with a Bruker AVANCE NEO console equipped with a Bruker Ascend Magnet operating at 11.76 T, resulting in resonance frequencies of 500.39 MHz for ^1H and 125.84 MHz for ^{13}C, and a commercial Bruker 1.3 mm H/F/X/Y MAS DVT probe. The samples were packed in 1.3 mm o.d. ZrO_2 rotors with Vespel caps using dry $MgSO_4$ powder as spacer material to confine the sample. The magic angle was calibrated using the spinning sidebands in the ^{23}Na MAS NMR spectrum of $NaNO_3$ spinning at 15 kHz prior to acquisition. The chemical shift was referenced using solid adamantane with $\delta(^1H) = 1.85$ ppm and $\delta(^{13}C) = 29.47$ ppm (highfield signal) spinning at 50.0 kHz.[12] The pulse lengths were calibrated using adamantane by finding the respective 180° pulse resulting in 90° pulses of 2 µs ($\nu_{nut}(^1H) = 125$ kHz) and 5 µs ($\nu_{nut}(^{13}C) = 50$ kHz). $^{13}C\{^1H\}$ spectra were acquired using ^1H CW decoupling with a power corresponding to 30 kHz. The relaxation delay for quantitative ^1H spectra was determined by increasing the relaxation delay from 0.5 s to 64 s until the signal no longer increased showing a recovery of >99 % after 4 s, so 16 s were chosen for the quantitative acquisition. ^1H double quanta were excited and reconverted with the BaBa-XY16 scheme,[13] using an increment for the indirect dimension evolution delay of 16 µs (rotor synchronous indirect detection). The FID was acquired after a z-filter period of 1.6 ms after DQ reconversion.

All spectra were processed using TopSpin 4.0.7 and dmfit[14] and plotted using home-written Python scripts using the matplotlib package. FIDs were cut at the point where the signal intensity fell below the noise, zero-filled and Fourier transformed. An exponential window (50 Hz) was applied for 1D ^1H MAS NMR spectra and a shifted squared sine (QSINE with SSB = 3) for the indirect dimension in ^1H-^1H double quantum spectra with no window function in the direct dimension. The baseline was corrected in all 1D spectra using a spline in dmfit. For the ^1H single pulse NMR spectra, the probe background was fitted with a Gaussian peak and subtracted.

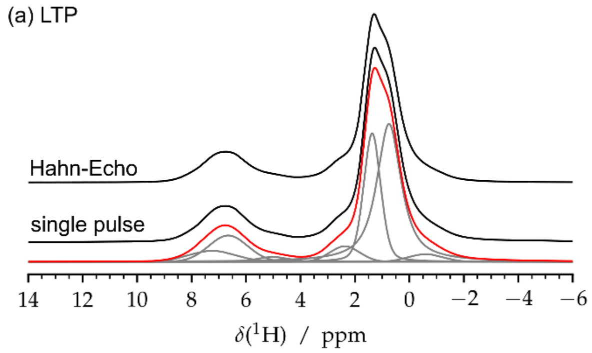

Figure S61. ^{1}H MAS NMR single pulse and Hahn-Echo spectra of (a) **LTP** and (b) **wGNR** spinning at 62.5 kHz with B_0 = 11.76 T (v(^{1}H) = 500.39 MHz) and ambient temperature. The signals used for deconvolution of the single pulse spectra are shown below in grey and their sum in red. The spectra are acquired under quantitative conditions using a relaxation delay of 16 s and are the average of 16 scans. For the Hahn-Echo the shortest possible echo time of 32 µs was chosen. Spinning sidebands are not shown because of their small intensity.

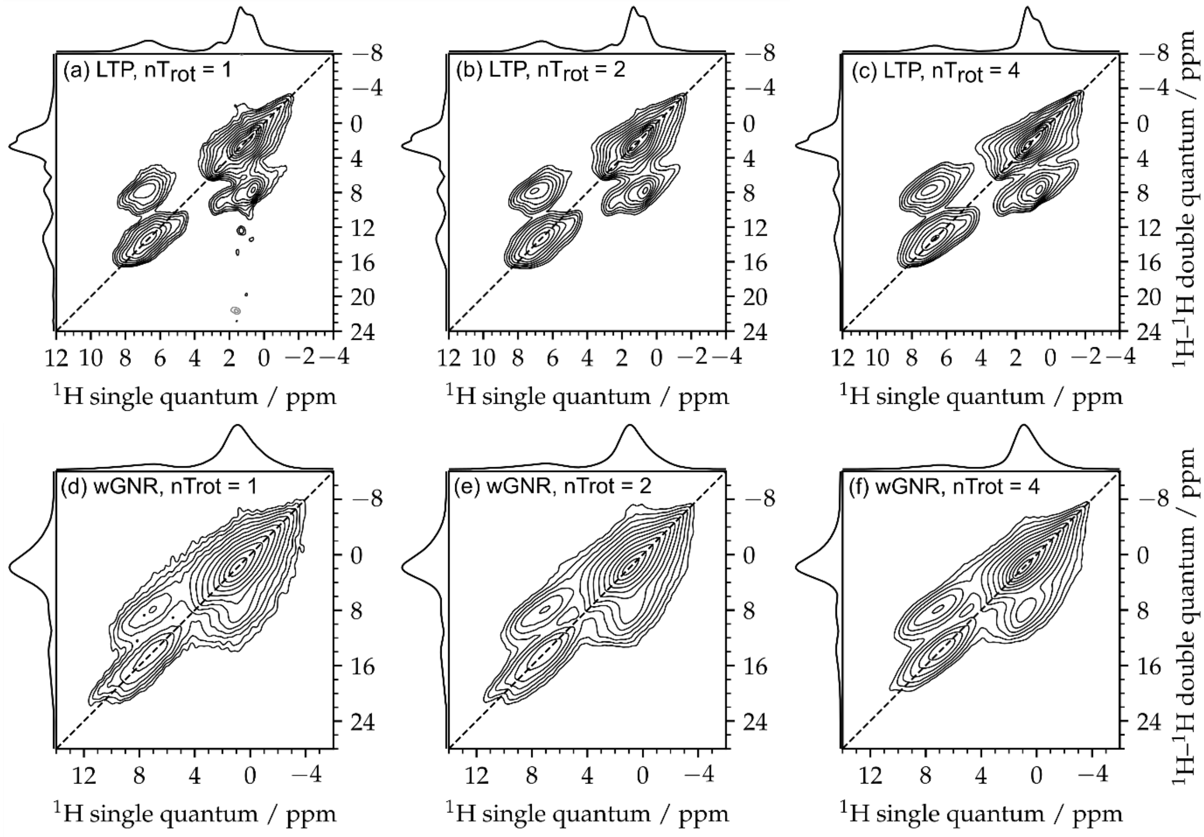

Figure S62. ^{1}H-^{1}H DQ/SQ NMR correlation spectra of (a, b, c) **LTP** and (d, e, f) **wGNR** spinning at 62.5 kHz and B_0 = 11.76 T (v(^{1}H) = 500.39 MHz) and ambient temperature. Different DQ excitation times of (a, d) 1, (b, e) 2 and (c, f) 4 rotor periods (i.e. 16 µs, 32 µs and 64 µs) were used. The diagonal dashed lines show the auto-correlation line where $\delta_{DQ} = 2 \cdot \delta_{SQ}$. The BaBa-XY16 scheme was used for DQ excitation and reconversion followed by a 1.6 ms z-filter prior to acquisition. 32 scans were averaged for every indirect increment.

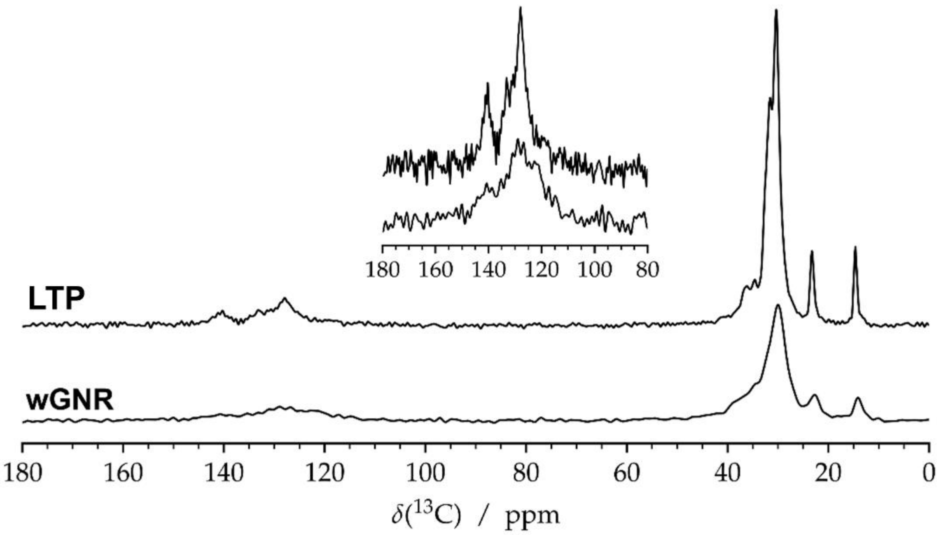

Figure S63. $^{13}C\{^{1}H\}$ MAS NMR spectra of **LTP** and **wGNR** spinning at 62.5 kHz and B_0 = 11.76 T ($\nu(^{13}C)$ = 125.84 MHz, $\nu(^{1}H)$ = 500.39 MHz) at ambient temperature. The inset shows a zoom of the aromatic region. Both spectra are the average of 20480 scans with 3.5 s relaxation delay. ^{1}H CW decoupling with 30 kHz power was used during acquisition.

12 References

[1] H. N. C. Wong, P. J. Garratt, F. Sondheimer, *J. Am. Chem. Soc.* **1974**, *96*, 5604–5605.

[2] D.-M. Du, W.-T. Hua, L.-P. Zhou, J.-W. Wang, X.-L. Jin, *J. Mol. Struct.* **2001**, *560*, 23–28.

[3] G. R. Fulmer, A. J. M. Miller, N. H. Sherden, H. E. Gottlieb, A. Nudelman, B. M. Stoltz, J. E. Bercaw, K. I. Goldberg, *Organometallics* **2010**, *29*, 2176–2179.

[4] R. Dovesi, A. Erba, R. Orlando, C. M. Zicovich-Wilson, B. Civalleri, L. Maschio, M. Rérat, S. Casassa, J. Baima, S. Salustro, B. Kirtman, *WIREs Comput. Mol. Sci.* **2018**, *8*, e1360.

[5] U. Mueller, R. Förster, M. Hellmig, F. U. Huschmann, A. Kastner, P. Malecki, S. Pühringer, M. Röwer, K. Sparta, M. Steffien, M. Ühlein, P. Wilk, M. S. Weiss, *Eur. Phys. J. Plus* **2015**, *130*, 141.

[6] K. M. Sparta, M. Krug, U. Heinemann, U. Mueller, M. S. Weiss, *J. Appl. Crystallogr.* **2016**, *49*, 1085–1092.

[7] W. Kabsch, *Acta Crystallogr. D Biol. Crystallogr.* **2010**, *66*, 133–144.

[8] G. M. Sheldrick, *Acta Crystallogr. Sect. C Struct. Chem.* **2015**, *71*, 3–8.

[9] M. J. Frisch, G. W. Trucks, H. B. Schlegel, G. E. Scuseria, M. A. Robb, J. R. Cheeseman, G. Scalmani, V. Barone, G. A. Petersson, H. Nakatsuji, X. Li, M. Caricato, A. V. Marenich, J. Bloino, B. G. Janesko, R. Gomperts, B. Mennucci, H. P. Hratchian, J. V. Ortiz, A. F. Izmaylov, J. L. Sonnenberg, D. Williams-Young, F. Ding, F. Lipparini, F. Egidi, J. Goings, B. Peng, A. Petrone, T. Henderson, D. Ranasinghe, V. G. Zakrzewski, J. Gao, N. Rega, G. Zheng, W. Liang, M. Hada, M. Ehara, K. Toyota, R. Fukuda, J. Hasegawa, M. Ishida, T. Nakajima, Y. Honda, O. Kitao, H. Nakai, T. Vreven, K. Throssell, J. A. Montgomery, Jr., J. E. Peralta, F. Ogliaro, M. J. Bearpark, J. J. Heyd, E. N. Brothers, K. N. Kudin, V. N. Staroverov, T. A. Keith, R. Kobayashi, J. Normand, K. Raghavachari, A. P. Rendell, J. C. Burant, S. S. Iyengar, J. Tomasi, M. Cossi, J. M. Millam, M. Klene, C. Adamo, R. Cammi, J. W. Ochterski, R. L. Martin, K. Morokuma, O. Farkas, J. B. Foresman, D. J. Fox, *Gaussian, Inc., Wallingford CT*, **2016**.

[10] T. Lu, F. Chen, *J. Comput. Chem.* **2012**, *33*, 580–592.

[11] F. Xu, C. Yu, A. Tries, H. Zhang, M. Kläui, K. Basse, M. R. Hansen, N. Bilbao, M. Bonn, H. I. Wang, Y. Mai, *J. Am. Chem. Soc.* **2019**, *141*, 10972–10977.

[12] S. Hayashi, K. Hayamizu, *Bull. Chem. Soc. Jpn.* **1991**, *64*, 685–687.

[13] K. Saalwächter, F. Lange, K. Matyjaszewski, C. F. Huang, R. Graf, *J. Magn. Reson.* **2011**, *212*, 204–215.

[14] D. Massiot, F. Fayon, M. Capron, I. King, S. Le Calvé, B. Alonso, J.-O. Durand, B. Bujoli, Z. Gan, G. Hoatson, *Magn. Reson. Chem.* **2002**, *40*, 70–76.
